# Anti-glioblastoma effects of phenolic variants of benzoylphenoxyacetamide (BPA) with high potential for blood brain barrier penetration

**DOI:** 10.1038/s41598-022-07247-8

**Published:** 2022-03-01

**Authors:** Joanna Stalinska, Cecilia Vittori, Charles H. Ingraham IV, Sean C. Carson, Karlie Plaisance-Bonstaff, Adam Lassak, Celeste Faia, Susan B. Colley, Francesca Peruzzi, Krzysztof Reiss, Branko S. Jursic

**Affiliations:** 1 Department of Chemistry, University of New Orleans, New Orleans, LA 70148 USA; 2 Stepharm LLC., PO Box 24220, New Orleans, LA 70184 USA; 3 Neurological Cancer Research, Stanley S. Scott Cancer Center, Department of Interdisciplinary Oncology, Louisiana State University Health Sciences Center, New Orleans, LA 70112 USA; 4 Department of Cell Biology, Faculty of Biochemistry, Biophysics and Biotechnology, Jagiellonian University, Cracow, Poland; 5 Department of Biomedical and Clinical Sciences L. Sacco, University of Milan, Milan, Italy

**Keywords:** Cancer, Drug discovery, Oncology, Chemistry

## Abstract

Glioblastomas are the most aggressive brain tumors for which therapeutic options are limited. Current therapies against glioblastoma include surgical resection, followed by radiotherapy plus concomitant treatment and maintenance with temozolomide (TMZ), however, these standard therapies are often ineffective, and average survival time for glioblastoma patients is between 12 and 18 months. We have previously reported a strong anti-glioblastoma activity of several metabolic compounds, which were synthetized based compounds, which were synthetized based on the chemical structure of a common lipid-lowering drug, fenofibrate, and share a general molecular skeleton of benzoylphenoxyacetamide (BPA). Extensive computational analyses of phenol and naphthol moieties added to the BPA skeleton were performed in this study with the objective of selecting new BPA variants for subsequent compound preparation and anti-glioblastoma testing. Initially, 81 structural variations were considered and their physical properties such as solubility (logS), blood–brain partitioning (logBB), and probability of entering the CNS calculated by the Central Nervous System—Multiparameter Optimization (MPO-CNS) algorithm were evaluated. From this initial list, 18 compounds were further evaluated for anti-glioblastoma activity in vitro. Nine compounds demonstrated desirable glioblastoma cell toxicity in cell culture, and two of them, HR51, and HR59 demonstrated significantly improved capability of crossing the model blood–brain-barrier (BBB) composed of endothelial cells, astrocytes and pericytes.

## Introduction

Glioblastomas are the most aggressive brain tumors for which therapeutic options are very limited^1,2^. Current standard of care therapies include maximal surgical resection, followed by radiotherapy plus concomitant treatment and maintenance with temozolomide (TMZ), however, these standard therapies are often ineffective, contributing to the dismal glioblastoma patient survival time of 12–18 months^3^. Multiple genetic and epigenetic abnormalities have been found in glioblastomas, among which p53, EGFR, PTEN, and IDH mutations are the most common^4–6^. In spite of these validated therapeutic targets, molecular, gene-therapy, and immunotherapy approaches are still ineffective^7,8^. Therefore, new and more effective therapies for glioblastoma patients are desperately needed.

There are several reasons why it is difficult to treat glioblastoma. First, glioblastomas are characterized by many dysregulated pathways that cannot be blocked simultaneously with a single therapy^9^; Second, glioblastomas are highly infiltrating and create heterogenous tumors that are very difficult to be removed by surgery without compromising the function of the surrounding brain areas^10^; Third, within the heterogenous tumor tissue, glioma initiating cells (GICs) have been identified as a primary mechanism involved in the development of drug resistance^11–15^. GIC’s display an embryonic stem cell phenotype^11,16–18^ and express stem-cell markers including CD133, CD44, SOX2, L1CAM, CD15, integrin α6, and BMI1^18–24^. In addition, GICs express ATP binding cassette (ABC) transporter proteins, which mediate extensive drug efflux contributing to radio- and chemo-resistance^25^, neovascularization^26^, and invasiveness^27^; Fourth, early diagnosis of glioblastoma is rare, therefore, large highly infiltrating and vascularized tumors are often present at the time of diagnosis^28^; Fifth, the optimization of clinical protocols for glioblastoma treatment requires the use of a reliable preclinical model/s. Unfortunately, commonly used rodent syngeneic and xenograft models have one major problem—the experimental tumors are typically ~ 10^3^–10^4^ times smaller than human tumors, and therefore, drug delivery, drug retention, and effective tissue penetration by the drug, cannot be tested in a reliable manner in small animal models^29^; and finally Six, the blood brain barrier (BBB) prevents the majority of anticancer drugs from reaching the tumor, and current methods that enhance the BBB penetration are still not effective for glioblastoma patients^30^.

**Figure 1:**
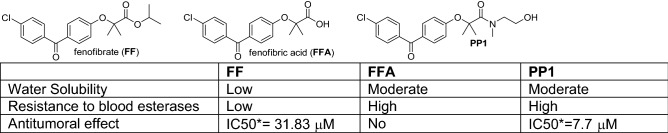
Comparison between FF, FFA, and PP1 structural and anti-cancer properties. The information regarding the compounds water solubility, stability in human blood, and in vitro cytotoxicity were previously reported^48^. *Determined using LN229 monolayer cultures and MTT assay.

One of the drugs that readily crosses the BBB is temozolomide (TMZ). Upon oral administration, TMZ maximum plasma concentration can be reached in about one hour, and the elimination half-life is approximately 2.1 h. Importantly, penetration efficiency of TMZ into the CNS is experimentally estimated to be about 20% of the plasma levels^31^. Applying this estimate to calculate the logBB (Brain-Blood Distribution) for TMZ, this equation produces a value of − 0.7, which indicates sufficient capability of the compound to cross the BBB^32^. In spite of these positive features, TMZ-treated glioblastoma patients develop TMZ-resistance and recurrent tumors are practically incurable^33^. There are also several studies of the use of TMZ in combination with other drugs, which show beneficial therapeutic effects^34,35^. One interesting example is a combination of TMZ with lipid lowering drugs, including statins^36^. In addition, another class of lipid-lowering compounds, fibrates, have also attracted attention as a possible anticancer drugs^37–40^. We have previously reported that 50 µM fenofibrate (FF) has a strong anti-glioblastoma activity in cell culture, and in glioblastoma mouse models following intratumoral injection^41^ (Fig. 1). However, FF does not cross the BBB, and is quickly processed by the blood and tissue esterases to form fenofibric acid (FFA), which is no longer effective in triggering tumor cell death^41,42^.

We have previously made several chemical modifications to the FF molecular skeleton, to address the FF low stability in human blood, low water solubility, and inability of penetrating the BBB. Indeed, one of the initial compounds, PP1, demonstrated improved water solubility and stability in human blood. In addition, PP1 was capable of triggering extensive glioblastoma cell death in vitro at concentrations over fourfold lower than FF^43^ (Fig. 1). To further improve anti-glioblastoma efficacy, we created other FF derivatives, which share the benzoyl-phenoxy-acetamide (BPA) molecular skeleton, and decided to test the addition of phenol and naphthol residues to the BPA structure due to the potential anti-cancer effects of these moieties^44–47^*.* As a result, 18 new compounds were generated and were analyzed during this study.

## Results and discussion

### Overall chemical design

**Figure 2:**
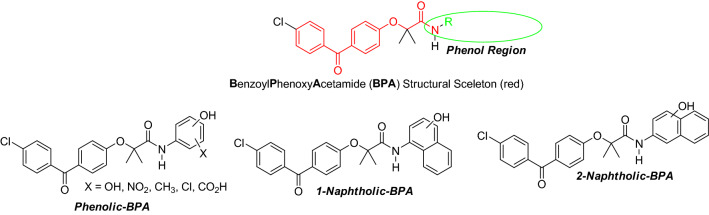
Phenol region of BPA skeleton selected for modification (circle) in search of the optimal anti-glioblastoma drug.

In our previous studies we have explored the importance of a basic BPA skeleton^48^, and concluded that BPA could serve as a “pharmacophore”, necessary to retain anti-glioblastoma activity^49,50^. The amide part of the BPA skeleton can be specifically modelled to obtain a more desirable anti-tumor activity. This includes, among other properties, chemical and physical parameters (described below) that contribute to the increased BBB penetration, and possibly drug retention within the tumor tissue. In this regard, we have selected phenol and naphthol residues due to mounting evidence supporting the role of different derivatives of these compounds in health benefits^51^, including anti-cancer activities^45,46^. In this paper, three variants of BPA are discussed: a substituted phenol (*Phenolic-BPA*), and two naphtholic BPAs (*1-Naphtholic-BPA* and *2-Naphtholic-BPA*) (Fig. 2) that serve as prototype molecules for further modifications.

**Figure 3:**
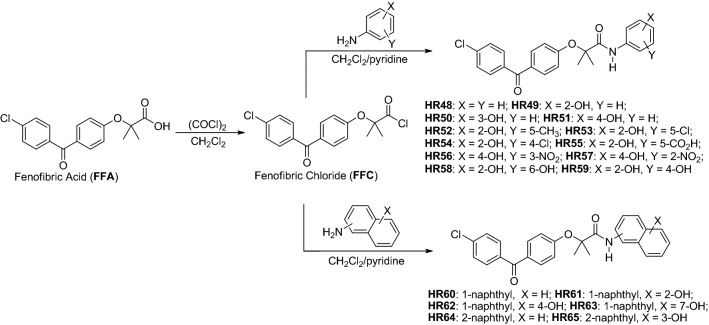
Schematic illustration of the procedure for preparation of hydroxylated phenyl and naphthyl derivatives of BPA.

The starting point for the preparation of all phenolic BPAs is fenofibric acid (FFA), and the corresponding aminophenol or aminonaphthol residues (Fig. 3) are added through amide (peptide) coupling reactions^52,53^. As previously reported^48^, due to the steric hindrance of the carboxylic group of FFA, which includes two methyl groups in the alpha position of carboxylic acid, combined with the lower amine nucleophilicity of anilines in DCC- or EDC-coupling, these reactions do not produce acceptable isolated yields. This occurs even with more reactive aminophenols and EDC or DCC, which are stronger nucleophiles compared to nonactivated anilines, which is expected to produce corresponding BPA compounds in acceptable yields^54^. However, we were able to detect only traces of the desirable products with these methods, and instead, decided to convert FFA into the more reactive fenofibrate chloride (FFC), followed by coupling with aminophenols or aminonaphthols (Fig. 3). We have explored several variations of this procedure and finally selected one that is very simple and can be applied to multigram and even multikilogram production scales. In particular, the FFC was prepared fresh and immediately used, for the next step of aminophenol addition (Fig. 3). The most common method of preparation of an acid chloride is by using thionyl chloride. This requires heating of thionyl chloride with the corresponding acid (in this case FFA) with appropriate traps for hydrochloric acid and sulfur dioxide, which are undesirable byproducts of the reaction^55^.

### Biological, chemical and computational testing of the compounds

Previously, we have reported that some amide derivatives of FF, including PP1, are more potent than others in eliminating glioblastoma cells^43^ and belong to the large family of BPA^56^. Further modifications of the BPA structure were designed to produce compounds with the improved ability to penetrate BBB, and superior glioblastoma cytotoxicity. This is highly relevant for glioblastoma patients, since one fundamental challenge for new drug development is the need for effective BBB penetration. So far, known BBB-permeable compounds form a very small subset of current oral drugs, and experimental models for testing BBB penetration by new drugs are complex and expensive. Therefore, an independent indicator of the BBB penetration is necessary for faster and more cost effective analyses of new anti-glioblastoma drug candidates. This is critical for the initial screening of a large number of compounds, including BPA variants, and for selection of best candidates for subsequent measurements of intracranial tumor drug penetration and anti-tumoral efficacy.

Therefore, we performed extensive molecular modeling prior to preparation of new BPA variants in order to evaluate their specific physicochemical properties considered relevant for the BBB penetration. We have applied first a weighted scoring approach named “Central Nervous System-Multiparameter Optimization” (CNS-MPO)^57,58^. The CNS-MPO algorithm uses 6 key physicochemical properties (clogP, clogD, MW, TPSA, HBD, and pKa) with the scores ranging between 0 and 6.0. Importantly, scores ≥ 4.0 are widely accepted for selecting compounds with high potential for CNS penetration^58^. The validation of this approach was based on a library of 616 compounds for which experimental distribution of the drug in CNS was determined and the corresponding parameters of the compound incorporated into CNS-MPO scores^57,58^. It was found that CNS-MPO scores of 1–2 (0%), 2–3 (11.6%), 3–4 (40.8%), 4–5 (53.8%) and 5–6 (81.1%) correlated with the increased probability of drugs to be found in the brain^59^.

**Figure 4:**
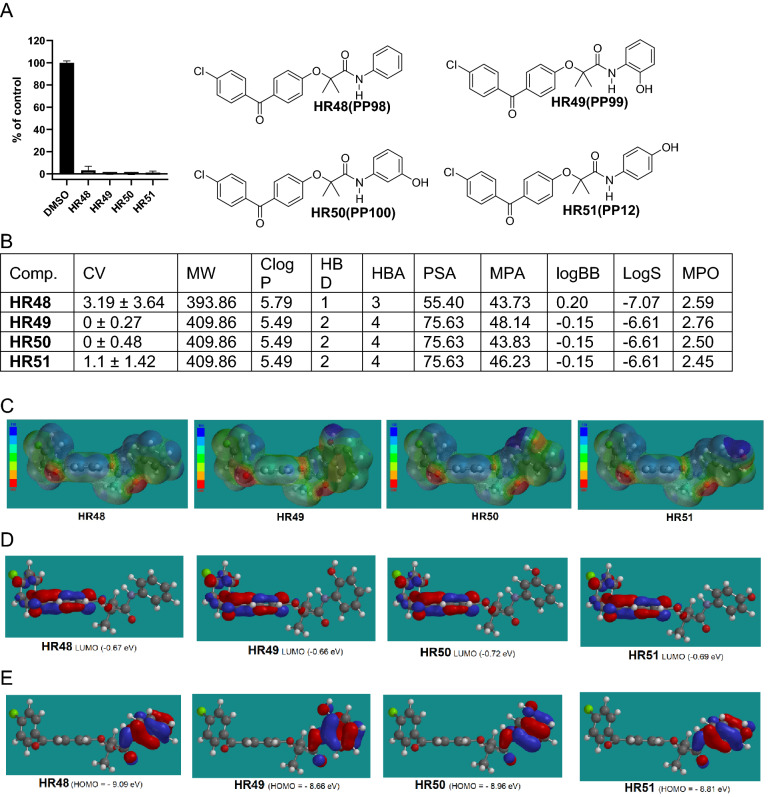
Drug candidates with hydroxy substituted phenylamide moiety. (**A**) Cell viability (MTT assay) following exposure to modified variants of HR48 with one hydroxy groups in different positions in the phenylamide moiety (25 μM, for 72 h). (**B**) *CV* Cell viability (% of control) mean ± SD at 25 μM; *ClogP* calculated partitioning; *HBD* hydrogen bond donor at pH 7; *HBA* hydrogen bond acceptor at pH 7; *logBB* calculated blood–brain partition; *PSA* Polar surface area (Å^2^); *MPA* Minimal projection area (Å^2^); *LogS* Aqueous solubility (mg/mL); *MPO* central nervous system multiparameter optimization (CNS MPO). (**C**) Electrostatic potential map for H48-HR51. (**D**) Computed LUMO orbitals contribution with their energies. (**E**) Computed HOMO orbitals contribution with their energies generated by semi-empirical method PM3 as implemented in Spartan ’18 version 1.1.0.

**Figure 5:**
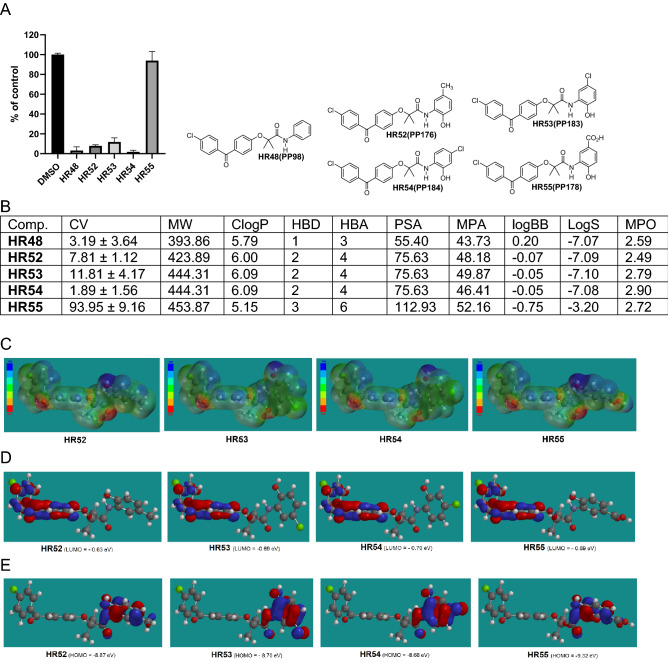
Drug candidates with substituted 2-hydroxyphenylamide moiety. (**A**) Cell viability (MTT assay) following exposure to modified variants of HR48 with ortho hydroxy and ether methyl, chloro, or carboxy group in the phenylamide moiety (25 μM, for 72 h). (**B**) *CV* Cell viability (% of control) mean ± SD at 25 μM; *ClogP* calculated partitioning; *HBD* hydrogen bond donor at pH 7; *HBA* hydrogen bond acceptor at pH 7; *logBB* calculated blood–brain partition; *PSA* polar surface area (Å^2^); *MPA* minimal projection area (Å^2^); *LogS* aqueous solubility (mg/ml); *MPO* Central nervous system multiparameter optimization (CNS MPO). (**C**) Electrostatic potential map for H52-HR55. (**D**) Computed LUMO orbitals contribution with their energies. (**E**) Computed HOMO orbitals contribution with their energies generated by semi-empirical method PM3 as implemented in Spartan ’18 version 1.1.0.

**Figure 6:**
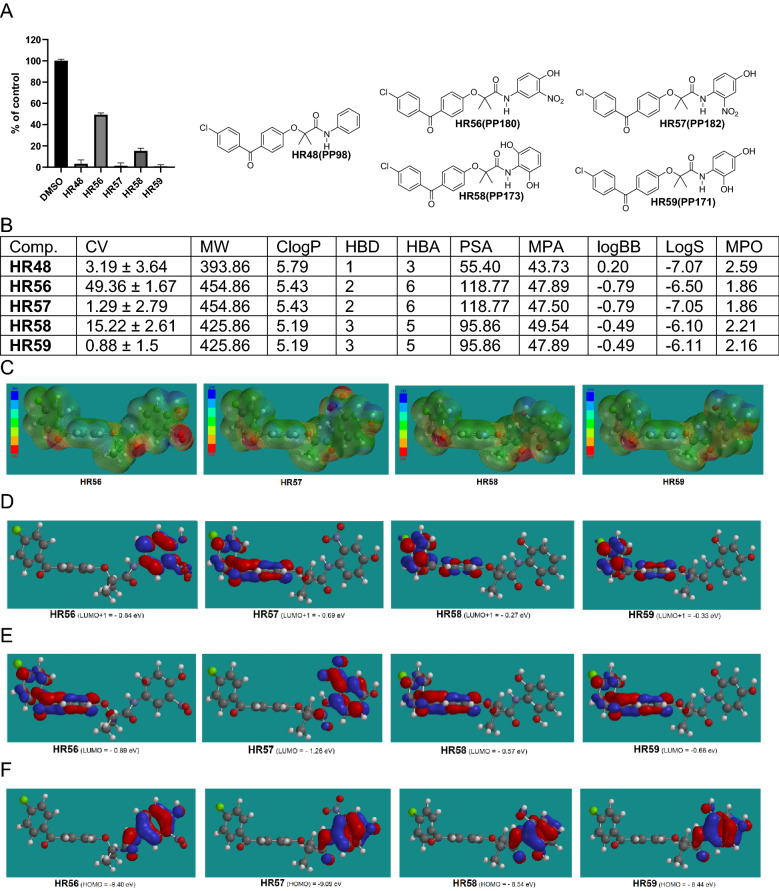
Drug candidates with nitro-hydroxy and two hydroxy substituted phenylamide moiety. (**A**) Cell viability (MTT assay) following exposure to modified variants of HR48 with one hydroxy and one nitro group or with two hydroxy groups in the phenylamide moiety (25 μM, for 72 h). (**B**) *CV* cell viability (% of control) mean ± SD at 25 μM; *ClogP* calculated partitioning; *HBD* hydrogen bond donor at pH 7; *HBA* hydrogen bond acceptor at pH 7; *logBB* calculated blood–brain partition; *PSA* polar surface area (Å^2^); *MPA* minimal projection area (Å^2^); *logS* aqueous solubility (mg/mL); *MPO* Central nervous system multiparameter optimization (CNS MPO). (**C**) Electrostatic potential map for H56-HR59. (**D**) Computed LUMO + 1 orbitals contribution with their energies. (**E**) Computed LUMO orbitals contribution with their energies. (**F**) Computed HOMO orbitals contribution with their energies generated by semi-empirical method PM3 as implemented in Spartan ’18 version 1.1.0.

**Figure 7:**
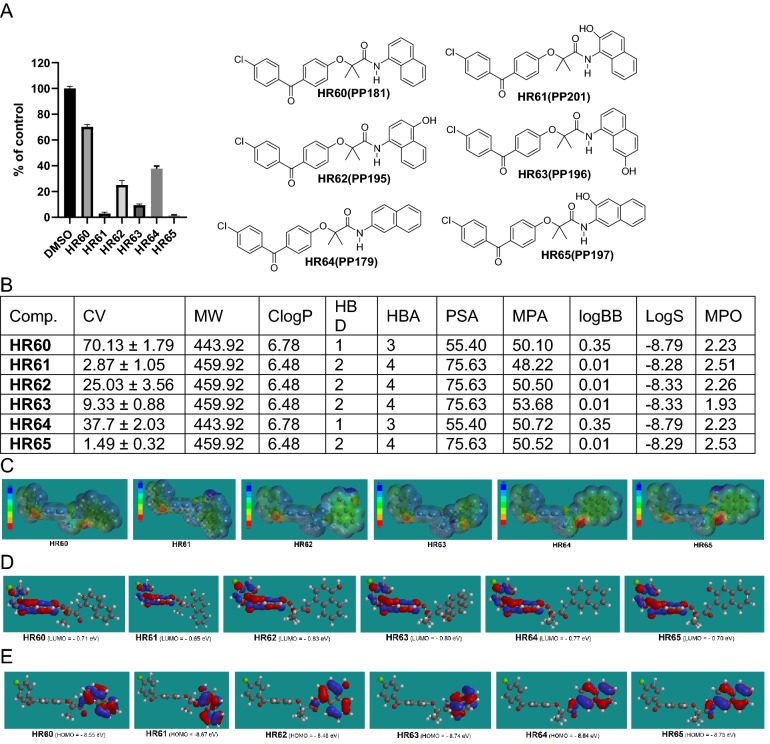
Drug candidates with hydroxy substituted naphthylamide moiety. (**A**) Cell viability (MTT assay) following exposure to modified variants of 1- and 2-naphthylamide of HR60 and HR64 with one hydroxy group in the naphthylamide moiety (25 μM, for 72 h). (**B**) *CV* Cell viability (% of control) mean ± SD at 25 μM; *ClogP* calculated partitioning; *HBD* hydrogen bond donor at pH 7; *HBA* hydrogen bond acceptor at pH 7; *logBB* calculated blood–brain partition; *PSA* polar surface area (Å^2^); *MPA* minimal projection area (Å^2^); *logS* aqueous solubility (mg/ml); *MPO* central nervous system multiparameter optimization (CNS MPO). (**C**) Electrostatic potential map for H60-HR65. (**D**) Computed LUMO orbitals contribution with their energies. (**E**) Computed HOMO orbitals contribution with their energies generated by semi-empirical method PM3 as implemented in Spartan ’18 version 1.1.0.

**Figure 8:**
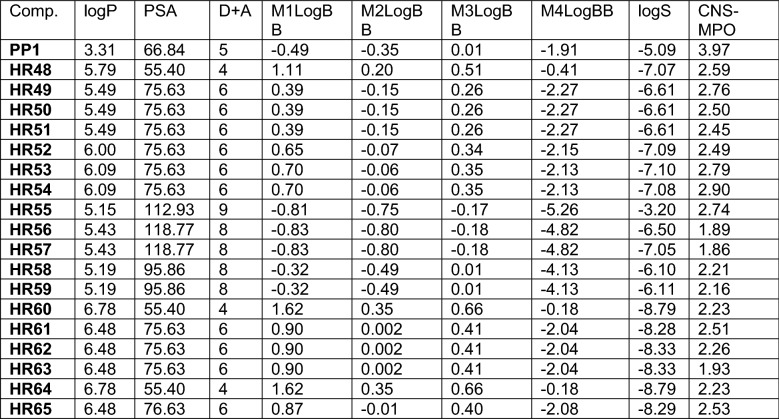
Compiled three variation of computing logBB and estimated MPO-CNS values for HR48-HR65. logP computed partition, *PSA* computed polar surface area; *ClogP* calculated partitioning; *PSA* polar surface area (Å^2^); D = hydrogen bond donor at pH 7; A = hydrogen bond acceptor at pH 7; *MPO* Central nervous system multiparameter optimization (CNS MPO). M1LogBB = 0.5159 × log P − 0.0277 × PSA − 0.3462. For log BB ≥ 0.3^32^. M2logBB = 0.152logP − 0.0148PSA + 0.139. (Clark’s model). M3logBB = 0.155 × logP − 0.01 × PSA + 0.164. (Rishton’s model). M4log BB = 0.2289 × log P − 0.0326 × PSA − 0.5671 × (D + A) + 2.3420. For log BB ≥  − 1^32^.

**Figure 9:**
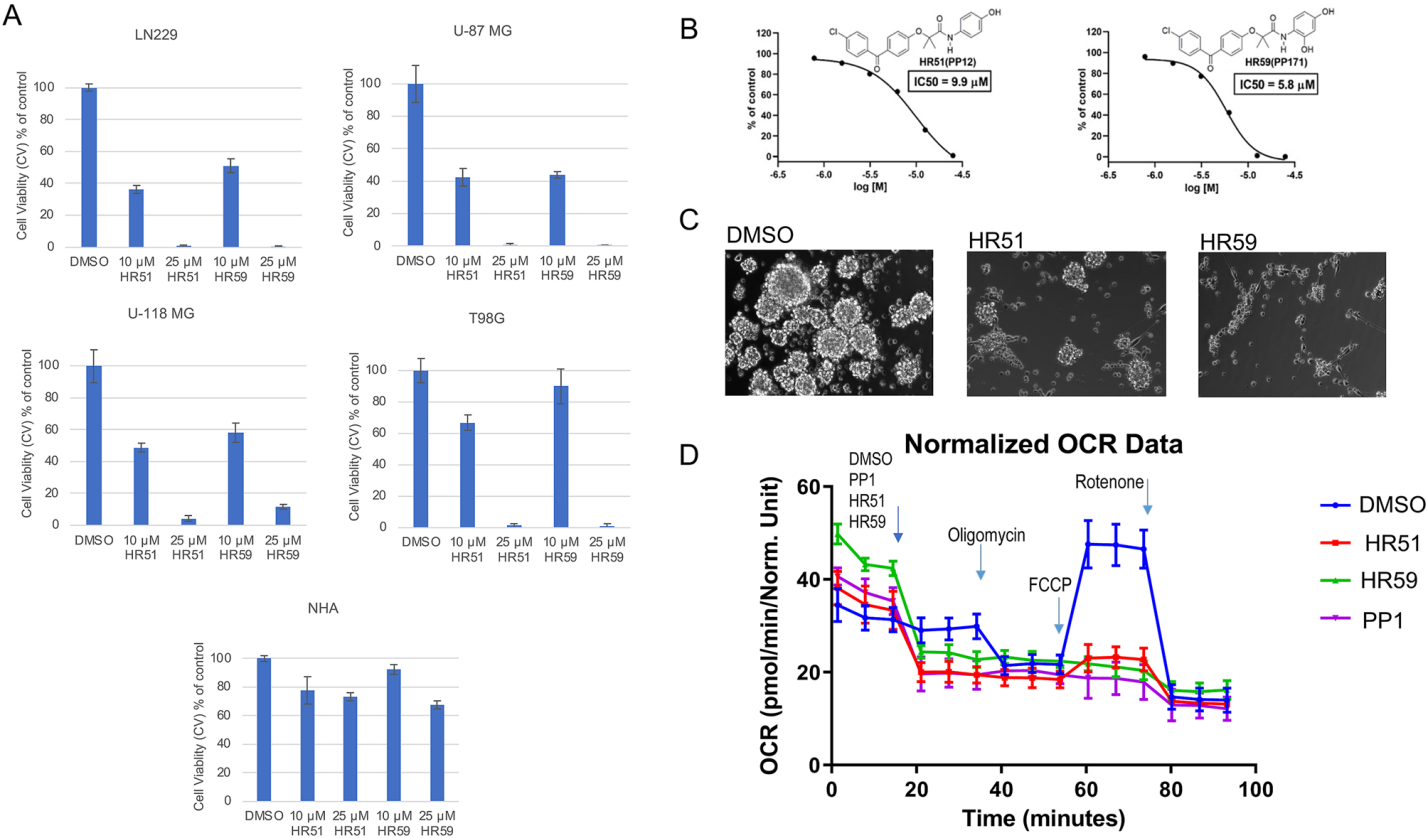
Cytotoxic effects of selected HR compounds. (**A**) Effects of 10 and 25 μM of HR51 and HR59 on the survival of human glioblastoma cell lines, LN229, U-87MG, U-118 MG, and T98G, compared to the effects of these two compounds on the survival of normal human astrocytes (NHA; LONZA/Clonetics™). Data were collected after 72 h of a continuous cell exposure to a single dose of HR51 or HR59 in the low glucose medium (1 g/L). Cells treated with DMSO (vehicle) were used as control. Data are expressed as cell viability (MTT, % of control) and represent average values with standard deviation (n = 3). (**B**) Dose response curves and IC50 values calculated for two most promising HR compounds, HR51 and HR59. Cell viability was evaluated by the MTT assay performed after exposure of LN229 to HR51 and HR59 for 72 h. Data represent mean values ± SD (n = 3). (**C**) Effects of HR51 and HR59 (25 μM each) on the sphere formation by patient-derived glioblastoma isolates (GBM12). In the sphere formation assay, low density single cell suspension of GBM12 cells was exposed to the serum-free stem-cell supporting medium (StemPro NSC SFM; Gibco: A1050901) supplemented with Recombinant Human FGF Basic and EGF (10 μg each), as previously reported^66,72^. The cells were allowed to form spheres for 5 days in the presence or absence of HR51 and HR59. DMSO-treated cultures (vehicle) were used as positive control for the sphere formation. In this condition, GBM12 cells grow in suspension and display stem-like phenotype—formation of multicellular gliospheres in low density cultures^66^. (**D**) Metabolic effects of HR51 and HR59 compared to the prototype drug, PP1. Metabolic responses to the drugs were evaluated in LN229 using Extracellular Flux Analyzer XF96 (Seahorse/Agilent). The oxygen consumption rate (OCR; indicative of mitochondrial respiration) was evaluated after injecting DMSO, (negative control), PP1 (positive control) and two experimental drugs, HR51 and HR59, followed by sequential injections of oilgomycin, FCCP, and rotenone (mitochondrial stress assay). Average OCR data were calculated from three independent experiments. Data represent average values ± SD. Compared to negative control (DMSO), all tested metabolic compounds (PP1, HR51 and HR59) triggered an immediate drop in OCR. In addition, the cells treated with these three compounds did not respond to FCCP injection, indicating loss of the proton gradient across the mitochondrial membrane.

In addition to CNS-MPO, other chemical and physical parameters of the Quantitative Structure Activity Relationship (QSAR) studies were also used in our calculations to further increase the probability of BBB penetration by the compound. These include: molecular polarizability (MP), minimal molecular projection area (MPA), water solubility (LogS), and lipophilicity (cLogP), to name a few. These parameters are not incorporated in the CNS-MPO score, however, they also play a role in the estimation of compound ability to penetrate the BBB^60^. Therefore, we have calculated and incorporated all these additional parameters when evaluating and selecting new HR compounds as potential anti-glioblastoma drugs (Figs. 4, 5, 6, 7, 8, 9); and Supplementary materials). In particular, MP is a response of electron distribution to an externally-applied static electrical field. Comparing MP values helps in understanding how different substrates may change polarization- and dispersion-type during interactions with the active sites of their interacting proteins^61^. It was postulated that MP values between 30 and 40 (see Supplementary data) are optimal for a molecule to bind to a biotarget^62^. Minimal projected area (MPA) represents another parameter that is important for drug transport and ultimately for drug activity. For instance, a distinct phenotypical pattern of drug recognition and transport for the G616N variant was reported, indicating that drug substrates with MPA over 70 Å^2^ are less likely to be transported compared to drugs with smaller MPA^63^. In addition, LogS of − 4.5 and greater are indicators of acceptable water solubility^64^, and the rate of passive diffusion is inversely proportional to the square root of molecular size (Graham’s law^65^), which are also included in our compound analysis.

Finally, the ability of the compound to penetrate the BBB can also be expressed as a decimal logarithm of brain to plasma concentration ratio (logBB), which is derived from the modified Clark’s equation: logBB = 0.152 ClogP-0.0148PSA + 0.139 30. It has been shown that compounds with logBB > 0.3 readily cross the BBB, while the compounds with logBB < − 1.0 have a problem with the CNS penetration^32^. Therefore, LogBB values for all HR compounds were also calculated and included (Figs. 4, 5, 6, 7, 8).

Once all above calculations were performed, initial cell viability (CV) tests were performed for all HR compounds using LN229 human glioblastoma cell line. The cells were treated with proposed HR compounds at 25 μM, and cell viability was evaluated using MTT assay, following a continuous cell exposure to a single dose of the drugs for 72 h. (Figs. 4, 5, 6, 7, 8, 9).

As a result of this initial screening, we have identified two lead drug candidates, HR51 and HR59, with phenolic moieties that contain BPA structural skeleton similar to our prototype anti-glioblastoma compound PP1^43^. This is in addition to our recently reported BPA-based compounds (HR28, HR32, HR37, and HR46), which also demonstrated high potential as anti-glioblastoma drugs^48^. Anti-glioblastoma effects of HR51 and HR59 were subsequently confirmed using four different human glioblastoma cell lines, LN229, U-87 MG, U-118 MG, T98G, and the cytotoxicity data were compared to normal human astrocytes (NHA). Results in Fig. 9A demonstrate that all tested glioblastoma cells were partially responsive to 10 µM HR51 and HR59, but were almost completely eliminated following 72 h exposure to 25 µM HR51 or HR59. In contrast, these two compounds were significantly less cytotoxic to normal human astrocytes (NHA), indicating that these two new compounds may have low CNS toxicity. In addition, results in Fig. 9B show that IC_50_ concentrations for HR51 and HR59 are below 10 μM, which is an acceptable therapeutic concentration for clinically relevant anticancer drugs.

Another important feature shared by HR51 and HR59 is the compounds ability to interfere with the sphere formation and proliferation of GBM12—patient-derived glioblastoma cells in low density 3-D cultures designed for propagation of glioma initiating cells (GICs)^66^. In this sphere formation assay, low density single cell suspension cultures of GBM12 were exposed to serum-free, growth factor supplemented medium (see “Methods”). As shown in Fig. 9C, only DMSO-treated GBM12 cells (vehicle control) were able to proliferate and formed large multicellular gliosphere structures. In the presence of 25 μM HR51 or 25 μM HR59 the cells did not proliferate (not shown), and their ability to form 3-D gliospheres was severely attenuated, indicating that not only bulk tumor cells but also tumor stem-like cells are the targets for HR51 and HR59.

We have also tested if the mechanism of action of HR51 and HR59 is similar to our prototype drugs, PP1 and fenofibrate, which have been previously shown to inhibit mitochondrial respiration at the level of Complex 1 of the electron transport chain (ETC)^41,43^. Indeed, results in Fig. 9C confirmed that both HR51 and HR59 inhibit mitochondrial respiration by the magnitude similar to PP1.

In addition to a strong in vitro anti-glioblastoma activity (Fig. 9), HR51 and HR59 have physical properties that may contribute to the improved brain tumor penetration. Specifically, HR51 and HR59, have a minimal projection area (MPA) of 46.23 Å^2^ and 43.73 Å^2^^63^, respectively; water solubility (LogS) of − 6.61 and − 6.11^64^; and brain to plasma concentration ratio (LogBB) of − 0.15 and − 0.49^32^, which are all considered as highly promising for compounds suspected of being capable of penetrating the brain tumor tissue.

**Figure 10:**
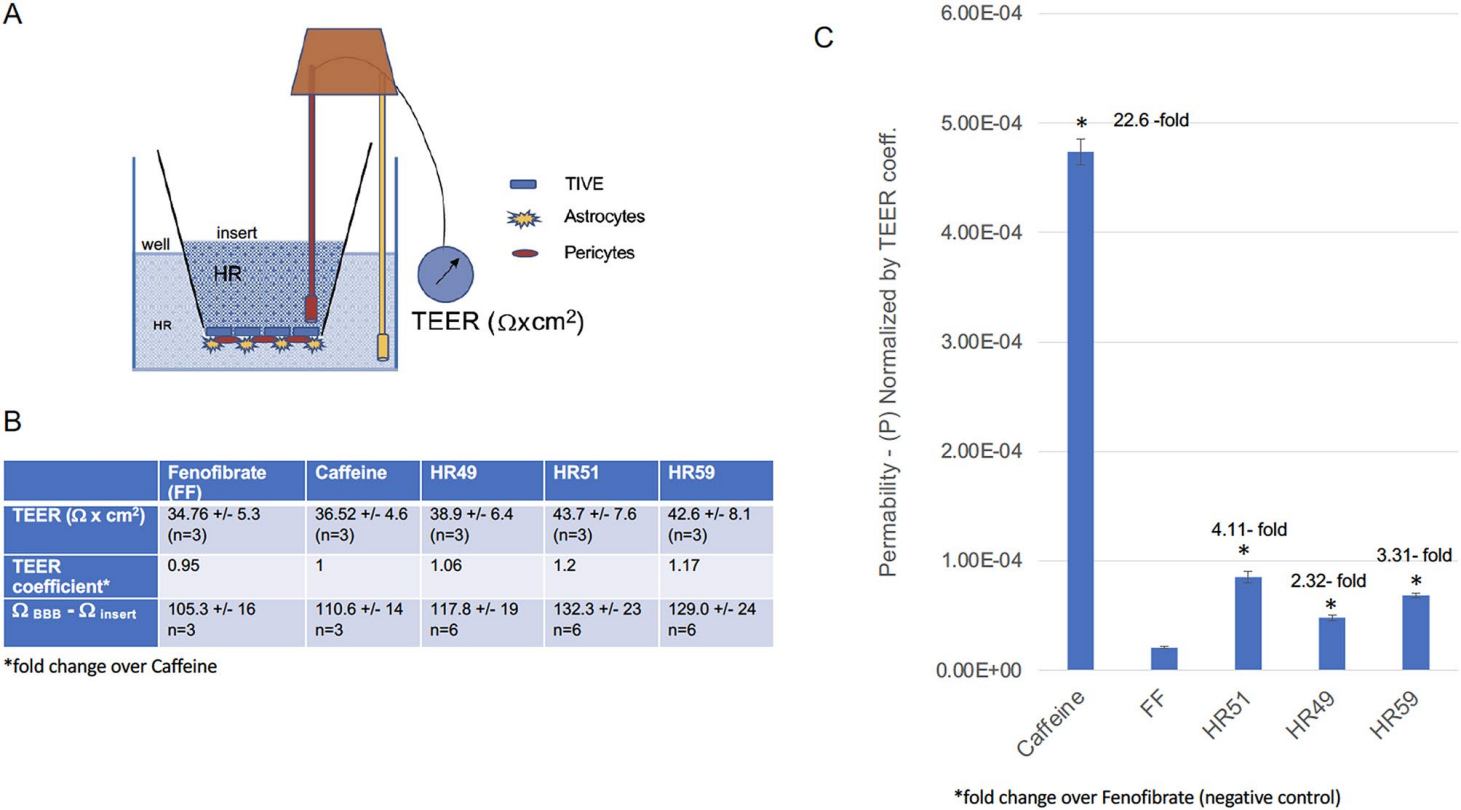
Penetration of the selected HR compounds through in vitro BBB model membrane: (**A**) Schematic representation of a triple-coculture model of the BBB, which consists of astrocytes, pericytes and epithelial cells cultured on 24-well transwell membranes with 3 μm pores. Trans-endothelial electric resistance (TEER) was measured using a EVOM^2^ meter with a STX3 electrode (World Precision Instruments). (**B**) Measurements of the electric resistance of inserts used for specific compounds (Ω); Difference between resistance of the insert with established BBB versus empty insert (Ω_BBB_ − Ω_insert_); and trans-endothelial electric resistance (TEER; Ω_BBB_ − Ω_insert_/cm^2^). * TEER coefficient = TEER_compound_/TEER_caffeine_. TEER coefficients were used to normalize BBB permeability (P) values to compensate for differences in TEER values between inserts selected for each compound. (**C**) Differences in BBB permeability (P) calculated using P = V_A_·C_A_/(t·S·C_L_) eqution^70^ and normalized by TEER coefficient. Data represent average values from 2 independent experiments in triplicates (n = 6) with standard deviation SD. *Indicates values significantly different from fenofibrate (internal negative control).

Importantly, HR51 and HR59 can also cross the triple-coculture model of the blood brain barrier (BBB), which consists of astrocytes, pericytes and epithelial cells cultured on 24-well (3 µm pores) transwell membranes (Fig. 10A), prepared according to the protocol provided by Stone et al.^67^. All experiments in which the ability of HR compounds to cross the BBB were evaluated using the BBB inserts that demonstrated a significant increase of the electric resistance (Ω) in comparison to inserts without cells *(Ω*_*BBB*_ − *Ω*_*Insert*_) (Fig. 10B). In particular, *Ω*_*BBB*_ − *Ω*_*Insert*_ values of 105.3 ± 16; 110.6 ± 14; 117.8 ± 19; 132.3 ± 23; and 129.0 ± 24 were measured for inserts used to evaluate BBB penetration of FF, caffeine, HR51 and HR59, respectively (Fig. 10B). Accordingly, trans-endothelial electric resistance (TEER = Ω_BBB_ − Ω_Insert_ × area of the membrane)^67,68^ for the inserts used for FF, caffeine, HR51 and HR59 testing are: 34.7 ± 5.3; 36.5 ± 4.6; 43.7 ± 7.6; and 42.6 ± 8.1, respectively. These TEER values are comparable to TEER values obtained from similar triple-coculture model of the BBB in which 24-well inserts with 3 μm pores were previously evaluated^67^, indicating effective electric resistance produced by our BBB model.

Following TEER measurements, 25 μM HR51, 25 μM HR59, as well as 50 μM caffeine (positive control^69^) and 25 μM fenofibrate (negative control^42^) were added to the corresponding insert and the plates were incubated in 37 °C and 5% CO_2_ for 24 h. Subsequently, aliquots of media from the corresponding inserts and from the wells were collected for HPLC measurements and to calculate BBB permeability (P = V_A_·C_A_/(t·S·C_L_)^70^. Results in Fig. 10C show that HR51, HR59 and caffeine cross the in vitro BBB at levels 4.4-fold, 3.5-fold and 22.0-fold higher compared to our internal negative control, fenofibrate, which although has a similar molecular weight and structure to the tested HR compounds (Fig. 4), its ability of crossing natural BBB is very low^42^.

By exploring the computed structural variations of phenolic-BPAs, and testing their cell toxicity, we have demonstrated that the addition of phenol moieties improves anti-glioblastoma activity with acceptable LogBB and LogS properties. This could be further improved by adding additional substituent(s) to the phenol moiety, including hydroxy group, halogen, alkyl, nitro on carbonyl to name a few. However, replacing the phenol residue with the larger naphthol, although it might not compromise anti-glioblastoma activity in cell culture, it may result in decreased compound bioavailability mostly due to low water solubility (Fig. 7)^71^. Therefore, these naphthol compounds are less likely to be considered as anti-glioblastoma candidates.

Presented in silico analyses of phenolic BPAs in combination with the in vitro BBB penetration model, represent a quick and relatively inexpensive way of testing a large number of new glioblastoma drug candidates. The observed effective penetration of the BBB model membranes by HR51 and HR59 is therapeutically promising, and it also validates our overall strategy for designing and selecting compounds with the most favorable physiochemical parameters for the BBB penetration (compare Figs. 4, 6 and 10). It should be emphasized, however, that these in silico and in vitro assays, although quite effective in narrowing down the number of potential glioblastoma drugs, cannot replace detailed pharmacokinetic analyses (tissue bioavailability and time of clearance) in clinically relevant intracranial glioblastoma animal model/s. Further experiments are necessary to confirm penetration and retention of the selected drug candidate/s in the brain tumor tissue at therapeutically-relevant concentrations.

## Methods

All starting materials were reagent grade and purchased from Sigma–Aldrich, ArkPharm, and TCI America. ^1^H-NMR spectra were recorded on Varian Mercury 300 and Varian Mercury 400 Plus instruments in CDCl_3_ and DMSO-d_6_, using the solvent chemical shifts as an internal standard^48^. All computed molecular descriptors were generated by ChemAxon MarvinSketch version 19.20. Electrostatic potential maps were calculated with a PM3 semi-empirical method as implemented in Spartan ’18 v 1.1.0^48^. ^1^H-NMR and ^13^C-NMR spectra for all HR compounds generated in this study are included in Supplementary Materials.

### Method A (Larger scale preparation without extraction or crystallization)

2-(4-(4-chlorobenzoyl)phenoxy)-*N*-(2-hydroxyphenyl)-2-methylpropanamide (HR49). Fenofibric chloride (FFC) was freshly prepared from FFA (4.8 g; 0.015 mol) and oxalyl chloride (2.6 mL; 3.8 g; 0.03 mmol). After drying with Argon, FFC was dissolved in dichloromethane (30 mL) and mixed with a pyridine (50 mL) solution of 2-aminophenol (1.3 g; 0.012 mol). The resulting solution was stirred at room temperature for 3 h, and then at 70 °C for an additional 3 h with distillation of dichloromethane. The pyridine was removed under reduced pressure. The resulting solid substance was mixed with ethanol (50 mL) and the resulting mixture was refluxed by stirring until all of the solid was dissolved. This resulting clear alcohol solution was mixed with 3% sodium carbonate solution (300 mL) and heated with stirring at 80 °C for one hour. After cooling to room temperature, the insoluble product was separated by filtration, extensively washed with water (10 × 100 mL) and dried at 110 °C for 1 h. The isolated yield was 97% (4.77 g). ^1^H-NMR (DMSO-d_6_, 400 MHz) δ 9.95 (1H, s, OH), 9.09 (1H, s, NH), 7.93 (1H, d, J = 8.0 Hz), 7.73 (2H, d, J = 8.0 Hz), 7.70 (2H, d, J = 8.4 Hz), 7.59 (2H, d, J = 8.4 Hz), 7.11 (2H, d, J = 8.4 Hz), 6.23 (1H, t, J = 7.6 Hz), 6.83 (1H, d, J = 8.0 Hz), 6.78 (1H, t, J = 8.0 Hz), and 1.60 (6H, s) ppm. ^13^C-NMR (DMSO-d_6_) δ 193.8, 171.7, 158.9, 147.7, 137.6, 136.5, 132.2, 131.7, 131.3, 129.1, 126.1, 125.1, 121.2, 119.9, 119.6, 115.5, 82.3 and 25.3 ppm.

### Method B (small scale preparation)

Preparation of 2-(4-(4-chlorobenzoyl)phenoxy)-*N*-(2-hydroxy-5-methylphenyl)-2-methylpropanamide (HR52). A dichloromethane (10 mL) suspension of fenofibric acid (FFA) (191 mg; 0.6 mmol) and oxalyl chloride (0.2 mL; 384 mg; 2 mmol) was stirred at room temperature for five minutes. A few drops of *N*,*N*-dimethylformamide (DMF) were then added to the suspension, which induced bubbling, and resulted in a clear reaction mixture after approximately 30 min. This solution was then stirred at 60 °C to promote slow solvent evaporation. The solvent residue and oxalyl chloride were removed by drying under an Argon flow at room temperature. The resulting yellow solid substance was dissolved in dichloromethane (10 mL) and mixed with 2-amino-4-methylphenol (62 mg; 0.5 mmol) in THF (10 mL) and Na_2_CO_3_ (1.06 g; 10 mmol) in water (10 mL). The subsequent mixture was stirred at room temperature for five hours. The solvent was then evaporated under reduced pressure and the residue was mixed with dichloromethane (50 mL) and water (50 mL). This final mixture was sonicated at room temperature until all solid was dissolved. From this bilayer solution, the water layer was discarded, and the dichloromethane layer was washed with 5% Na_2_CO_3_ (3 × 50 mL), water (50 mL), 5% HCl (3 × 50 mL), water (50 mL) and dried over anhydrous Na_2_CO_3_. After solvent evaporation, the final product was purified by crystallization from dichloromethane (~ 3 mL) and hexane (20 mL). The isolated yield was 90% (190 mg). ^1^H-NMR (DMSO-d_6_, 400 MHz) δ 9.74 (1H, broad s, OH), 9.06 (1H, s, NH), 7.81 (1H, s), 7.73 (2H, d, *J* = 8.8 Hz), 7.70 (2H, d, *J* = 8.8 Hz), 7.58 (2H, d, *J* = 8.8 Hz), 7.11 (2H, d, *J* = 8.4 Hz), 6.73 (2H, s), 2.18 (3H, s), and 1.60 (6H, s) ppm. ^13^C-NMR (DMSO-d_6_) δ 193.8, 171.6, 158.8, 145.4, 137.7, 136.5, 132.2, 131.7, 131.3, 129.1, 128.1, 125.9, 125.3, 121.5, 119.9, 115.2, 82.2, 25.3, and 20.9 ppm.

### Cell culture and viability assays

All cell culture procedures used for HR compound testing were previously described^43,48^. Briefly, human glioblastoma cell line, LN-229 (ATCC# CRL-2611), U-87MG (ATCC# HTB-14), U-118 MG (ATCC# HTB-15) and T98G (ATCC# CRL-1690) were maintained as semi-confluent monolayer cultures in DMEM (1 g/L glucose; with sodium pyruvate and l-glutamine) supplemented with 100 U/ml penicillin, 100 μg/mL streptomycin, and 10% fetal bovine serum (FBS) at 37 °C in a 5% CO_2_ atmosphere. GBM12 are patient-derived human glioblastoma cells established in Dr. Sarkaria's lab^33,73^. GBM12 cells are routinely propagated in subcutaneous tissue of nude mice (IACUC protocol # 3750,LSUHSC, New Orleans), and were isolated from the tumor tissue for short-term monolayer and suspension cultures as previously reported^41^. For three-dimensional (3-D) gliosphere cultures, the cells aggregates were dissociated with Accutase (Innovative Cell Technologies: AT104), and low density single cell suspension cultures of GBM12 (1 × 10^4^/mL) were incubated in the serum-free growth factor-supplemented stem-cell medium (StemPro NSC SFM: A1050901, which consists of KnockOut DMEM/F-12 Basal Medium (500 mL), plus StemPro NSC SFM Supplement (10 mL), 10 μg recombinant human basic FGF and 10 μg EGF)^66^. In the sphere-formation assay, the cells were allowed to form 3-D gliospheres, and the sphere morphology was evaluated 5 days following the plating.

Normal human astrocytes (NHA) were used to evaluate effects the selected HR compounds may have on normal non-transformed cells. NHA were cultured according to the manufacturer protocol (LONZA/Clonetics™). Prior to the treatment with HR compounds, the cells were plated in 96-well plates (BD Falcon) at the initial density of 2 × 10^4^/well. Twenty-four hours after plating, stock solutions of the HR compounds were prepared in DMSO, diluted in cell culture medium, to the final concentration of 25 µM, and added to the cells in triplicate for every experimental condition. For the vehicle control, DMSO was used at 0.5%. After 72 h incubation, MTT assay (surrogate for cell viability) was performed according to our previous publications^48^. Briefly, following 1.5 h incubation with MTT, formazan crystals were dissolved in 5 mM HCl in isopropanol and the absorbance read at 540 nm. Data represent mean values expressed as % cell viability of control (DMSO) ± SD. Phase contrast images of treated cells were taken 72 h after treatment with HR compounds with a BZ-X800 fluorescence microscope (Keyence) equipped with a 20 × objective. The drug dose causing 50% inhibition in the MTT assay at 72 h time point (half maximal inhibitory concentration—IC50) was calculated using GraphPad Prism 8.

### In vitro model of the blood brain barrier (BBB)

The blood brain barrier was re-created in vitro using a modified protocol provided by Stone et al.^67^. Briefly, 24-well transwell inserts (Falcon, catalog number 353096) were coated with 10 μg/cm^2^ of Collagen Type IV from human placentas (Sigma) for 24 h at 4 °C. Inserts were washed with sterile water and air-dried for 2 h. Next, the inserts were coated with 2 μg/cm^2^ poly-l-lysine (ScienCell) for 1 h at 37 °C, then washed twice with sterile H_2_0 and air-dried for 2 h. 1.57 × 10^5^ primary human astrocytes (ScienCell) and 3.125 × 10^4^ primary human pericytes (ScienCell) were resuspended in 25 μL of astrocyte medium and pericyte medium (ScienCell), respectively, then combined in a 1:1 ratio for 50 μL total volume. Dried, coated inserts were turned upside down such that the basolateral surface was exposed at the top, and 50 μL of the cell mixture was added to the membrane, covered with the plate lid, and incubated for 2 h at 37 °C to allow cell adherence. Any medium remaining on top of the membrane was carefully removed before returning inserts to their upright position with the apical surface facing upward as they were placed in a 24-well plate containing 500 μL per well of astrocyte/pericyte medium (1:1). An additional 300 μL of medium was added to the apical compartment. Four days after plating, the apical compartment medium was removed, and 3.75 × 10^4^ of telomerase-immortalized vain endothelial cells (TIVE; provided by Dr. Rolf Renne) in 50 μL of TIVE medium^74^ were added and incubated for 5 h at 37 °C to allow cell adherence, followed by the addition of an extra 250 μL of TIVE medium. Half the volume of the corresponding media in the lower and upper compartment was replaced with fresh media every third day. Ten days after initial plating, trans-endothelial electric resistance (TEER) was measured using a EVOM^2^ meter with a STX3 electrode (World Precision Instruments). The ability of selected HR compounds to pass through in vitro BBB was tested using inserts with effectively reconstructed BBB as confirmed by TEER^67,68^.

### HPLC detection of selected HR compounds

Following TEER measurement, the medium from apical compartment of the in vitro BBB model (Fig. 10A) was replaced with 350 μL of fresh TIVE medium containing corresponding compounds [HR49 (PP99), HR51 (PP12), HR59 (PP171)] all used at 25 μM. In addition, 25 μM fenofibrate (FF), which does not cross the BBB^42^ was used as negative control, and 50 μM caffeine was used as a positive control^69^. Plates containing the inserts were returned to the incubator (37 °C, 5% CO_2_), and after 24 h of incubation conditioned media from basolateral (well) and apical (insert) compartments were collected and frozen for quantitative analyses by HPLC. 100 μL aliquots of the collected samples were subsequently mixed with 100 μL of 100% acetonitrile, samples were centrifuged at 16,000 rpm at 4 °C for 10 min and supernatants collected for HPLC analyses using UltiMate 3000 system (Thermo Scientific) equipped with analytical YMCbasic, 3 µm, 150 × 4.6 mm column (octyl silane C8; YMC America, Inc.). Isocratic elution of the compounds was performed using mobile phase composed of solvent A (50 mM acetic acid in dH_2_O) and solvent B (acetonitrile) mixed at ratios predetermined for each compound (Table 1). All separations were carried out with sample volume of 5 μL at flow rate of 1 mL/min, at 20 °C. Concentration of each compound was calculated using serial dilutions of the known concentration of the compound separated at the same run with experimental and control samples. After separation, integrated areas under the peak were used to prepare calibration curves and to determine concentration of the compounds.

**Table 1:** Details of the HPLC method for selected HR compounds.

Compound	Method length (min)	Concentration solvent B (%)	Detection wavelength (nm)	Retention time (min)
Caffeine	5	25	272	2.54
Fenofibrate	10	70	288	5.84
HR51	6	60	260	4.3
HR59	5	60	262	3.92

### Evaluation of metabolic parameters

Metabolic responses of human glioblastoma cells were evaluated with Extracellular Flux Analyzer XFe96 (Agilent Technologies) according to our previously established protocol^43^. During the day prior to each assay the cells were plated at 2 × 10^4^ cells/well in Agilent Seahorse 96-well XF cell culture microplates with growth supporting media and incubated overnight. At the time of measurement, growth media were replaced with serum-free XF assay medium (Seahorse XF Base Medium supplemented with 1 mM sodium pyruvate, 2 mM glutamine, and 5.5 mM glucose) and cartridges equipped with oxygen-sensitive and pH-sensitive fluorescent probes were placed above the cells. The oxygen consumption rate (OCR; indicative of mitochondrial respiration) was evaluated after injecting HR compounds or PP1 (all used at 25 μM), or DMSO (0.1%; vehicle control). These initial injections were followed by sequential injections of metabolic toxins to execute mitochondrial stress assay: oligomycin (inhibitor of ATP synthase; 0.5 μM); carbonylcyanide-p-trifluoromethoxyphenylhydrazone (FCCP; uncoupling factor; 0.5 μM), rotenone (inhibitor of mitochondrial complex I; 0.3 μM), and antimycin A (inhibitor of mitochondrial complex III; 0.3 μM).

## Supplementary Information

Supplementary Information.
