# Supplementary material for: Anti-glioblastoma effects of phenolic variants of benzoylphenoxyacetamide (BPA) with high potential for blood brain barrier penetration
Source: Sci Rep. 2022 Mar 1;12:3384. doi: 10.1038/s41598-022-07247-8 (PMC8888627; doi:10.1038/s41598-022-07247-8)
Supplement: Supplementary file 1 — Supplementary Information. [file 41598_2022_7247_MOESM1_ESM.pdf]

Supplementary Materials

*^{1}H-NMR, ^{13}C-NMRs and Computed properties of **HR48-HR65***

Anti-glioblastoma effects of phenolic variants of benzoylphenoxyacetamide (BPA) with high potential for Blood Brain Barrier penetration.

Joanna Stalinska[c,d], Cecilia Vittori[c,e], Charles H. Ingraham IV[a], Sean C. Carson[a], Karlie Plaisance-Bonstaff[c], Adam Lassak[c], Celeste Faia[c], Susan B. Colley[c], Francesca Peruzzi[c], Krzysztof Reiss[c*], and Branko S. Jursic[a,b*]

[a]Department of Chemistry, University of New Orleans, New Orleans, LA 70148, United States; [b]Stepharm llc., PO Box 24220, New Orleans, LA 70184, United States; [c]Neurological Cancer Research, Stanley S. Scott Cancer Center, Department of Medicine, LSU Health Sciences Center, New Orleans, LA 70112; [d]Department of Cell Biology, Faculty of Biochemistry, Biophysics and Biotechnology, Jagiellonian University, Cracow Poland; [e]Department of Biomedical and Clinical Sciences L. Sacco, University of Milan, Milan, Italy.

*corresponding authors

^{1}H-NMR(DMSO-d_6) Varian Mercury 400 Plus

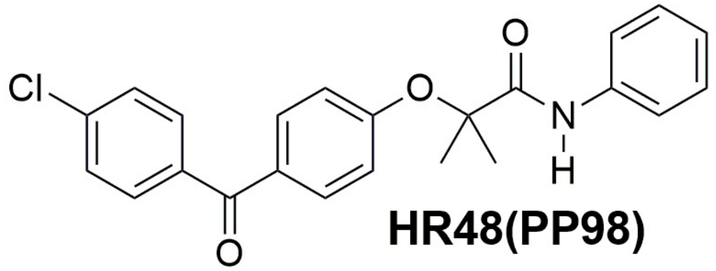

HR48(PP98)

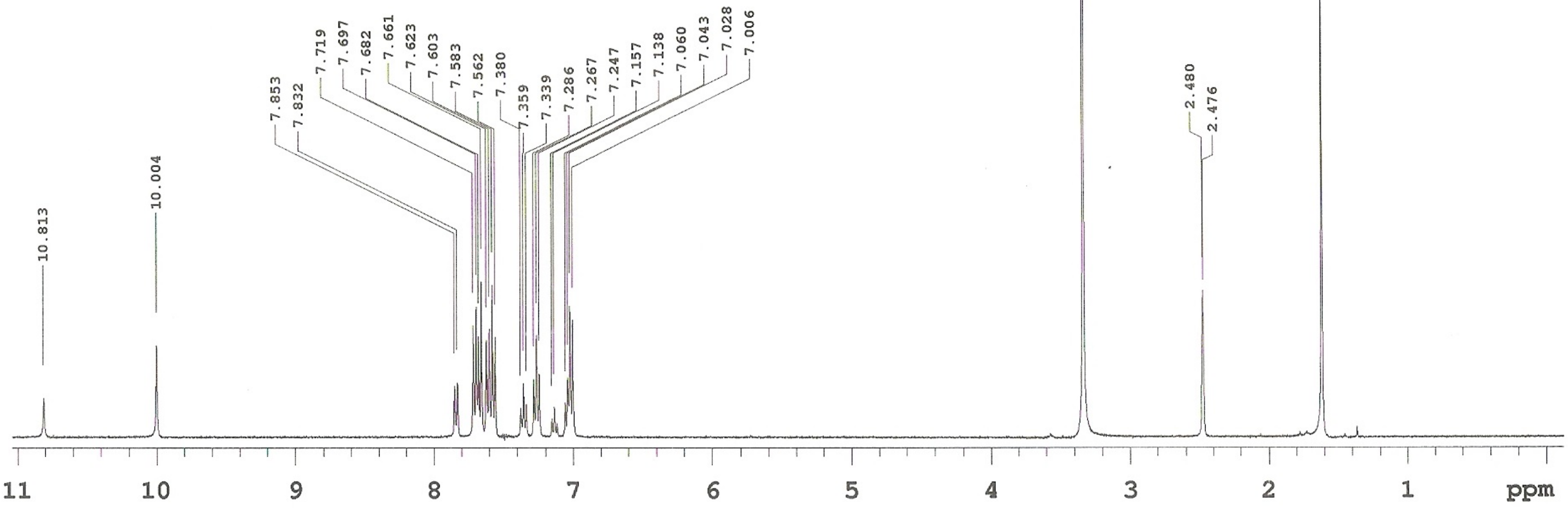

^{13}C-NMR(DMSO-d_6) Varian Mercury 400 plus

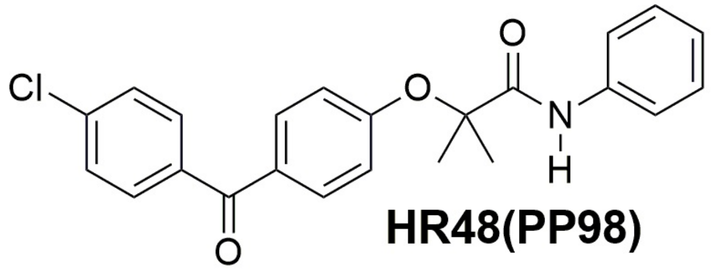

HR48(PP98)

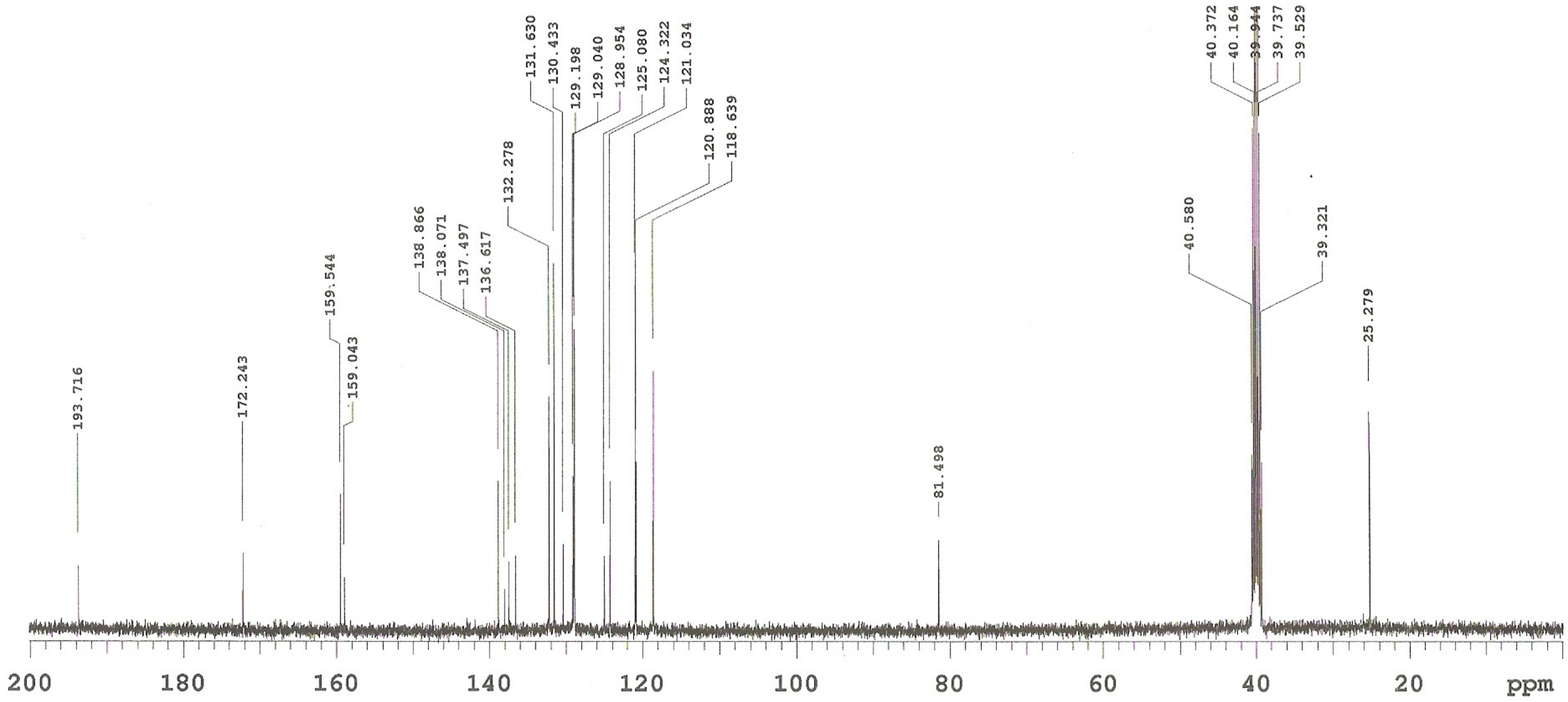

^{1}H-NMR ($CDCl_3$) Varian Mercury 400 Plus

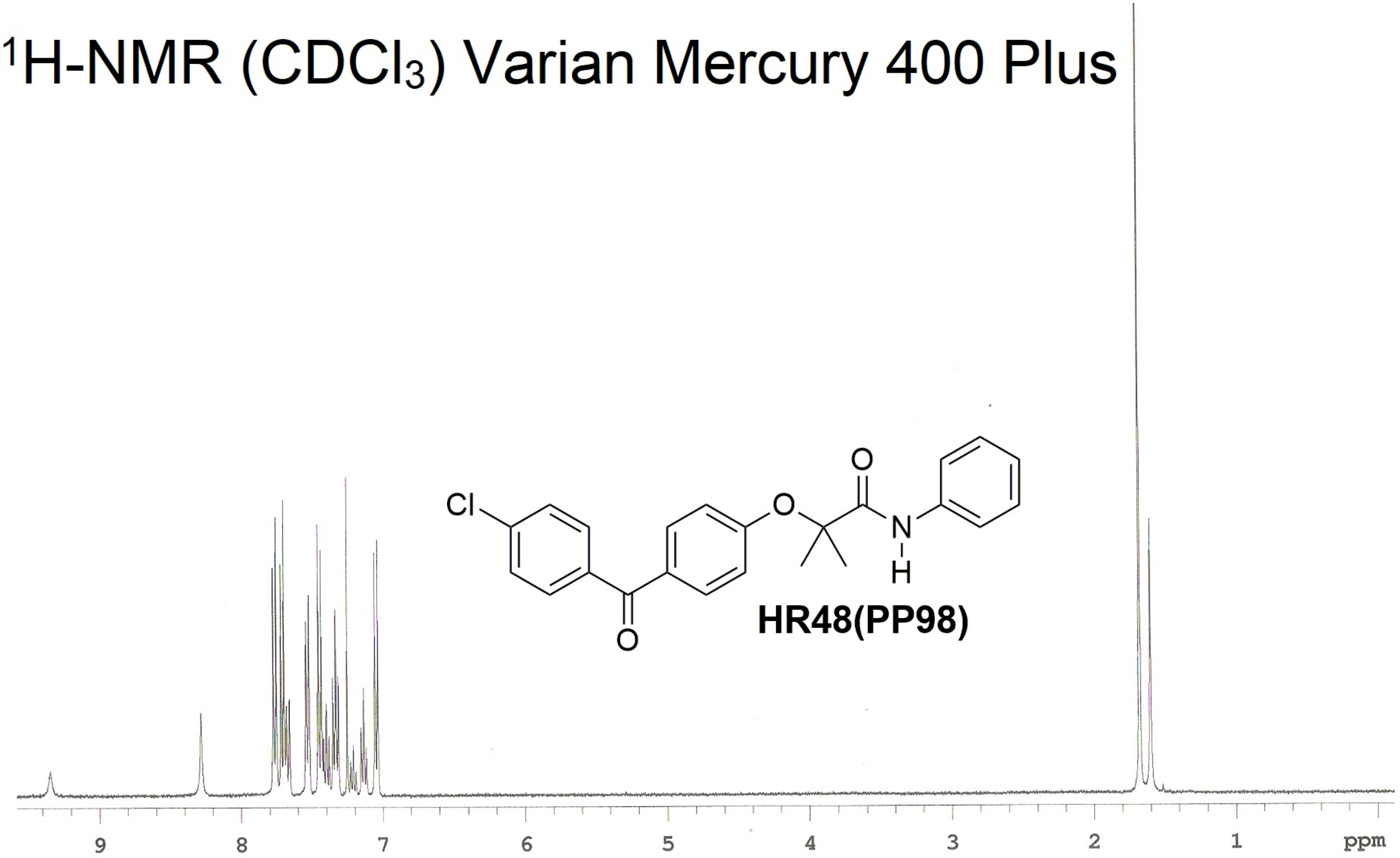

^{1}H-NMR(DMSO-d_6) Varian Mercury 400 Plus

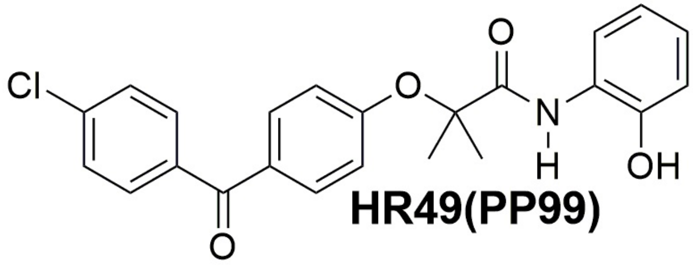

HR49(PP99)

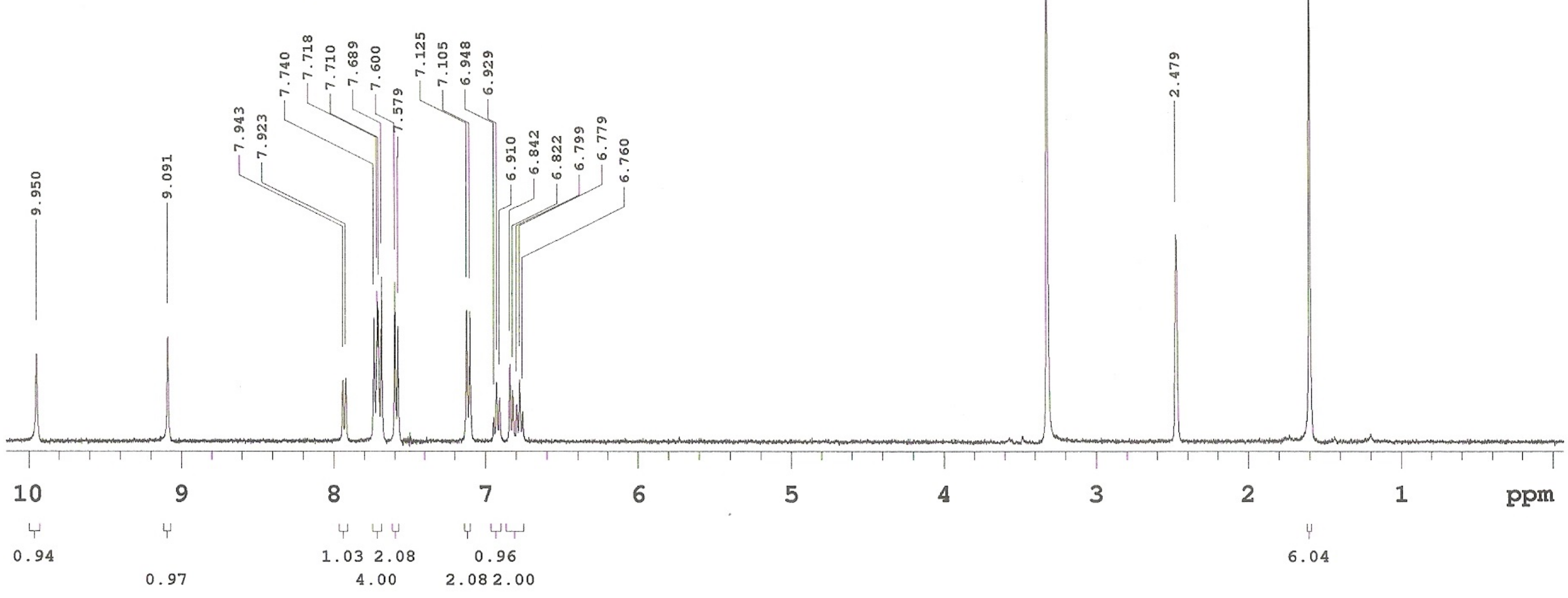

^{1}H-NMR(DMSO-d_6) Varian Mercury 400 Plus

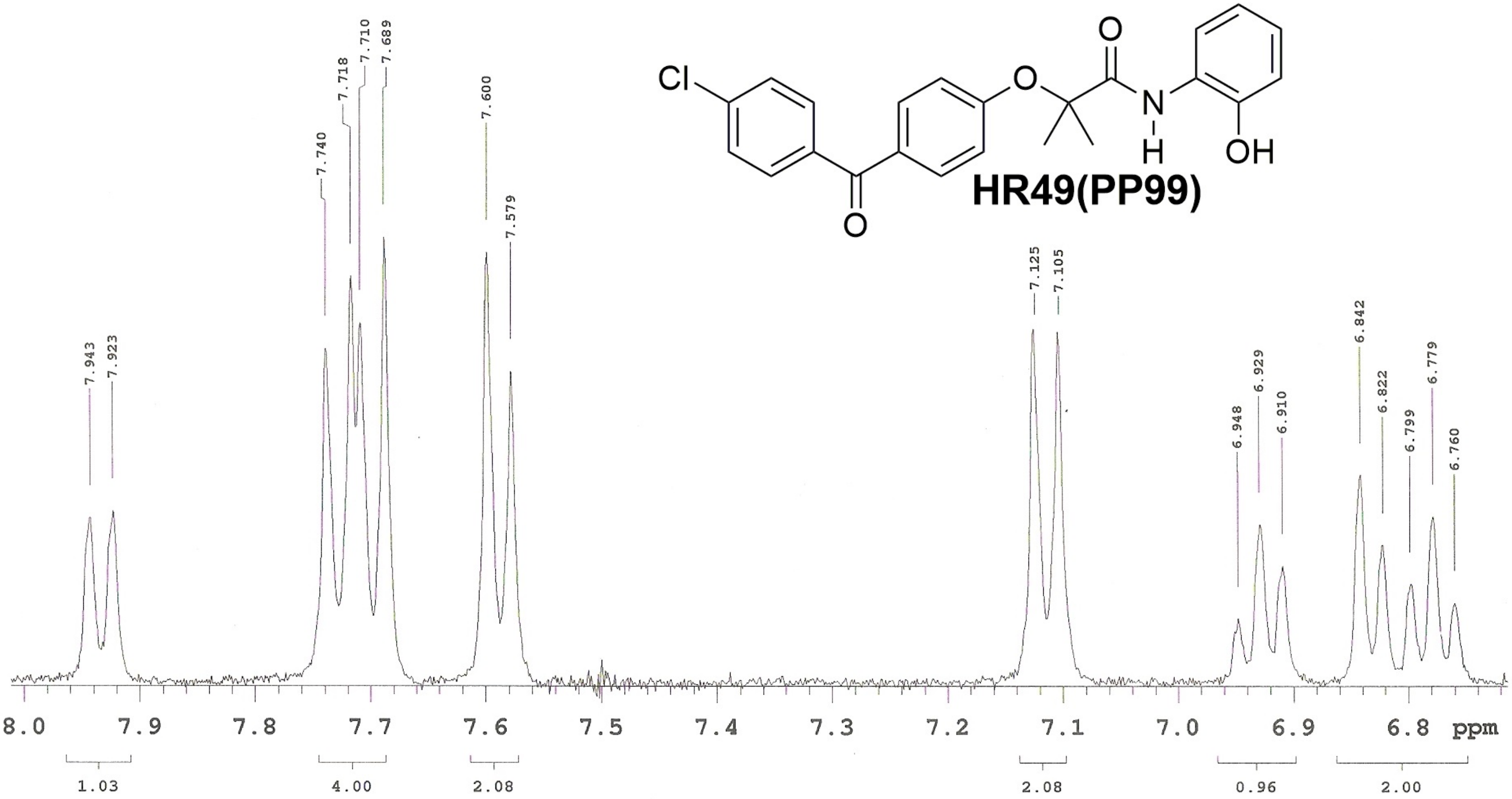

HR49(PP99)

^{13}C-NMR(DMSO-d_6) Varian Mercury 400 plus

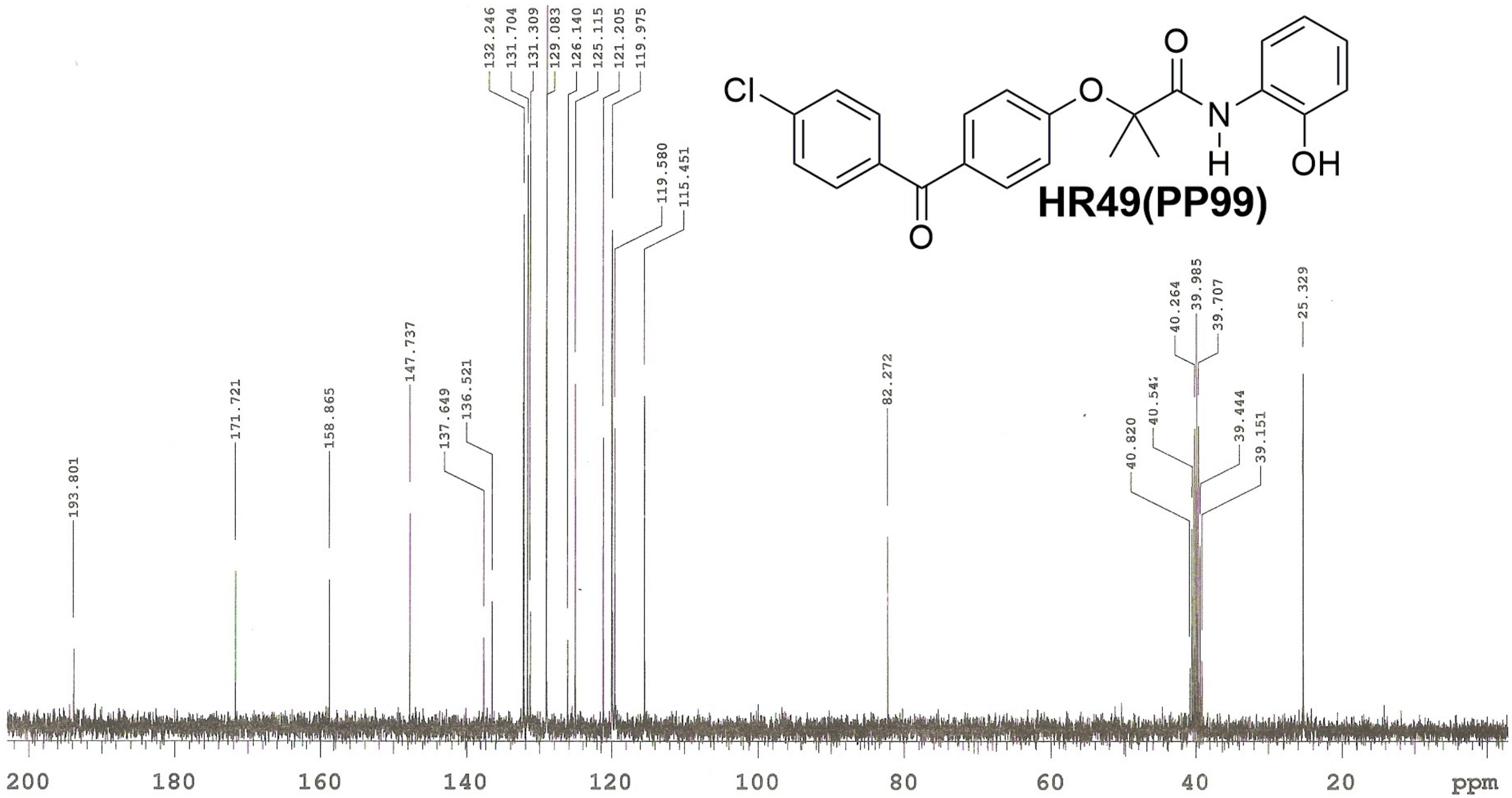

HR49(PP99)

^{1}H-NMR(DMSO-d_6) Varian Mercury 400 Plus

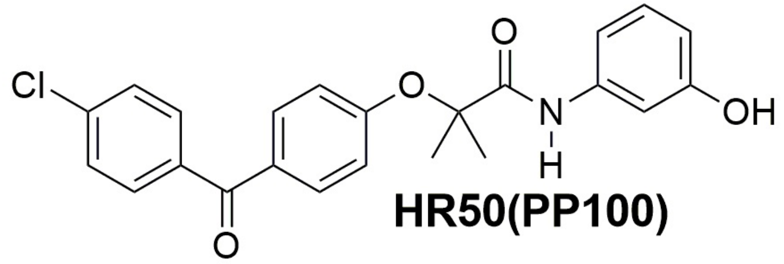

HR50(PP100)

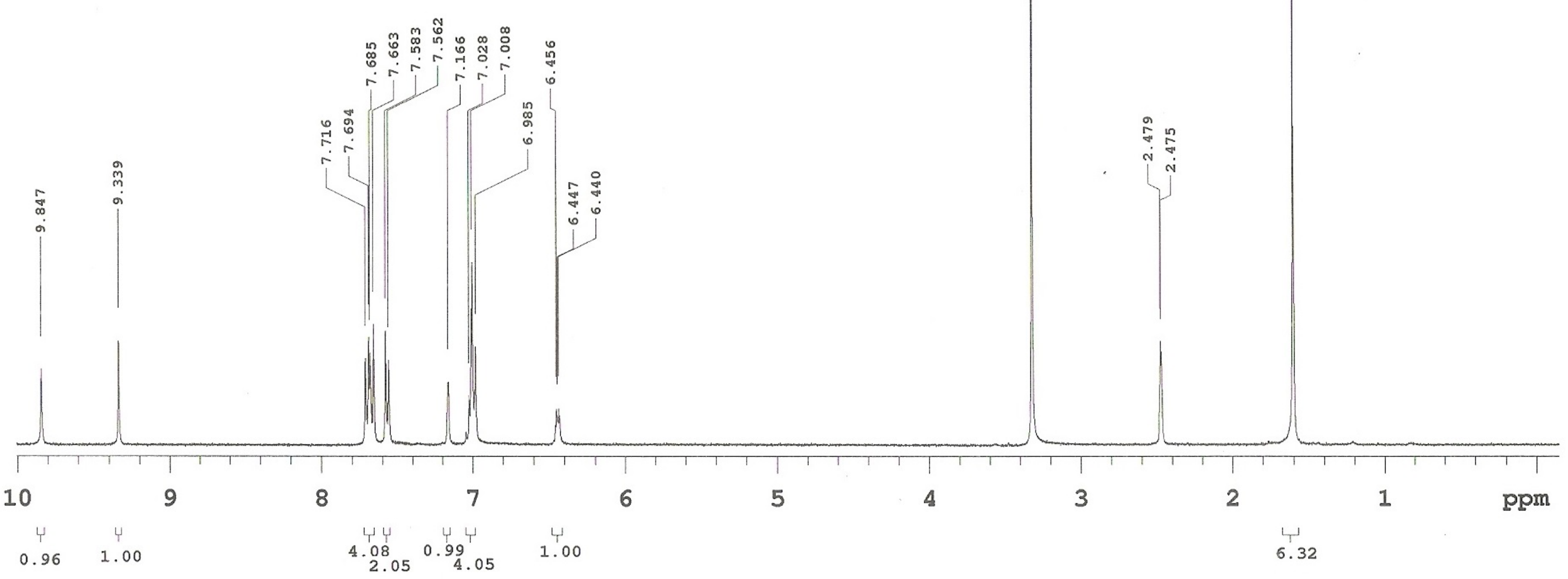

^{13}C-NMR(DMSO-d_6) Varian Mercury 400 plus

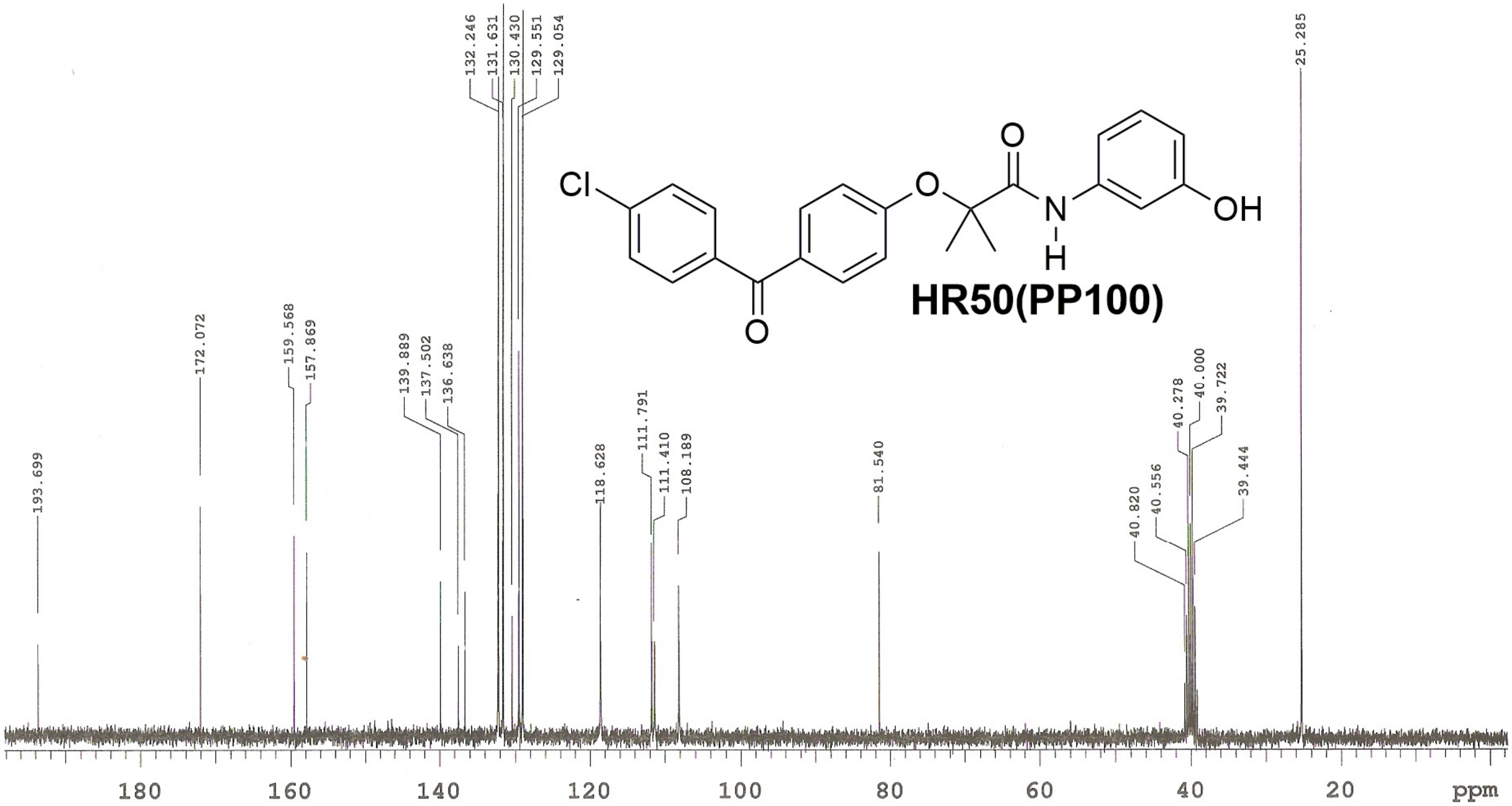

^{1}H-NMR($CDCl_3$) Varian Mercury 400 Plus

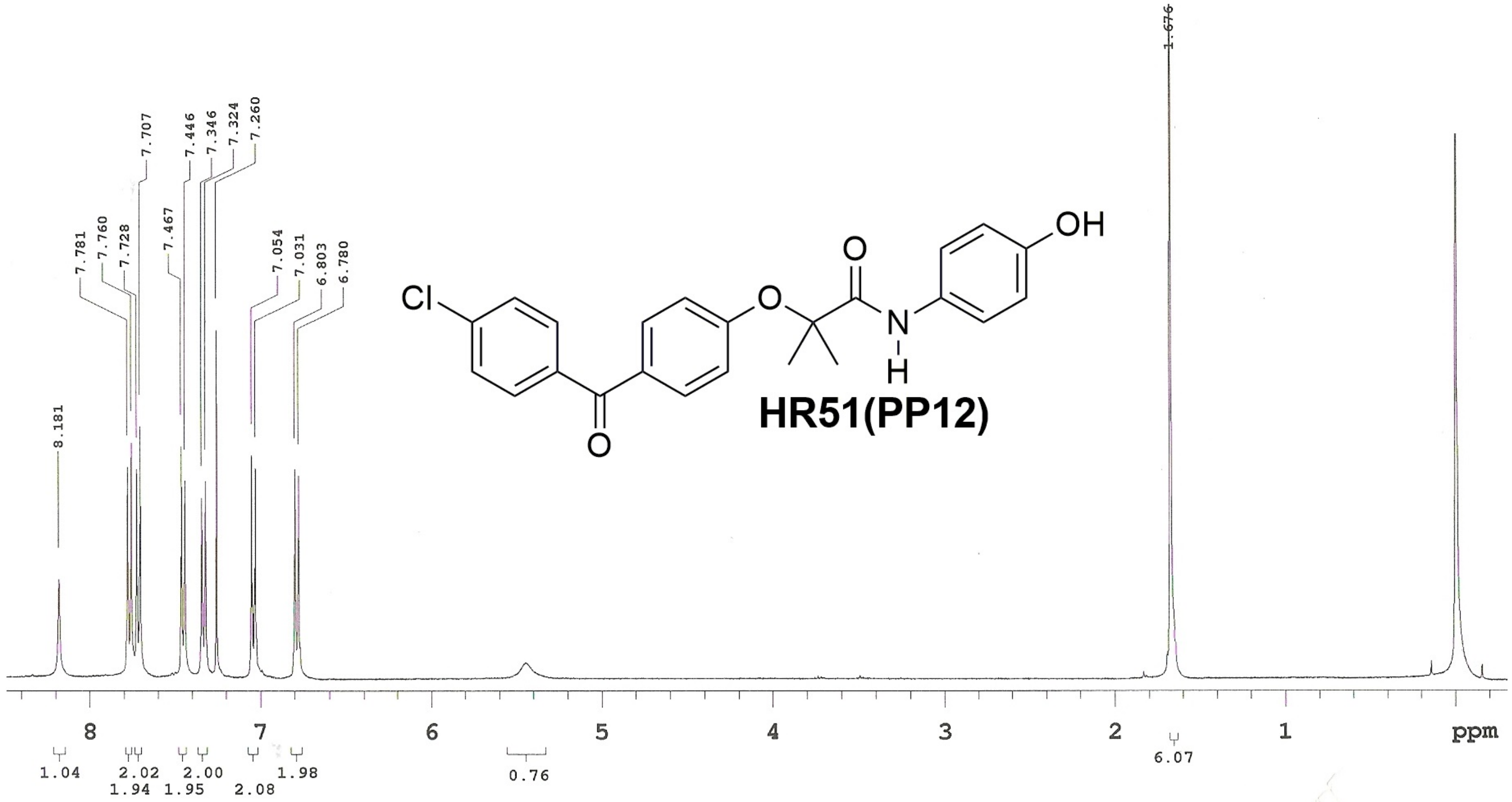

HR51(PP12)

^{1}H-NMR($CDCl_3$) Varian Mercury 400 Plus

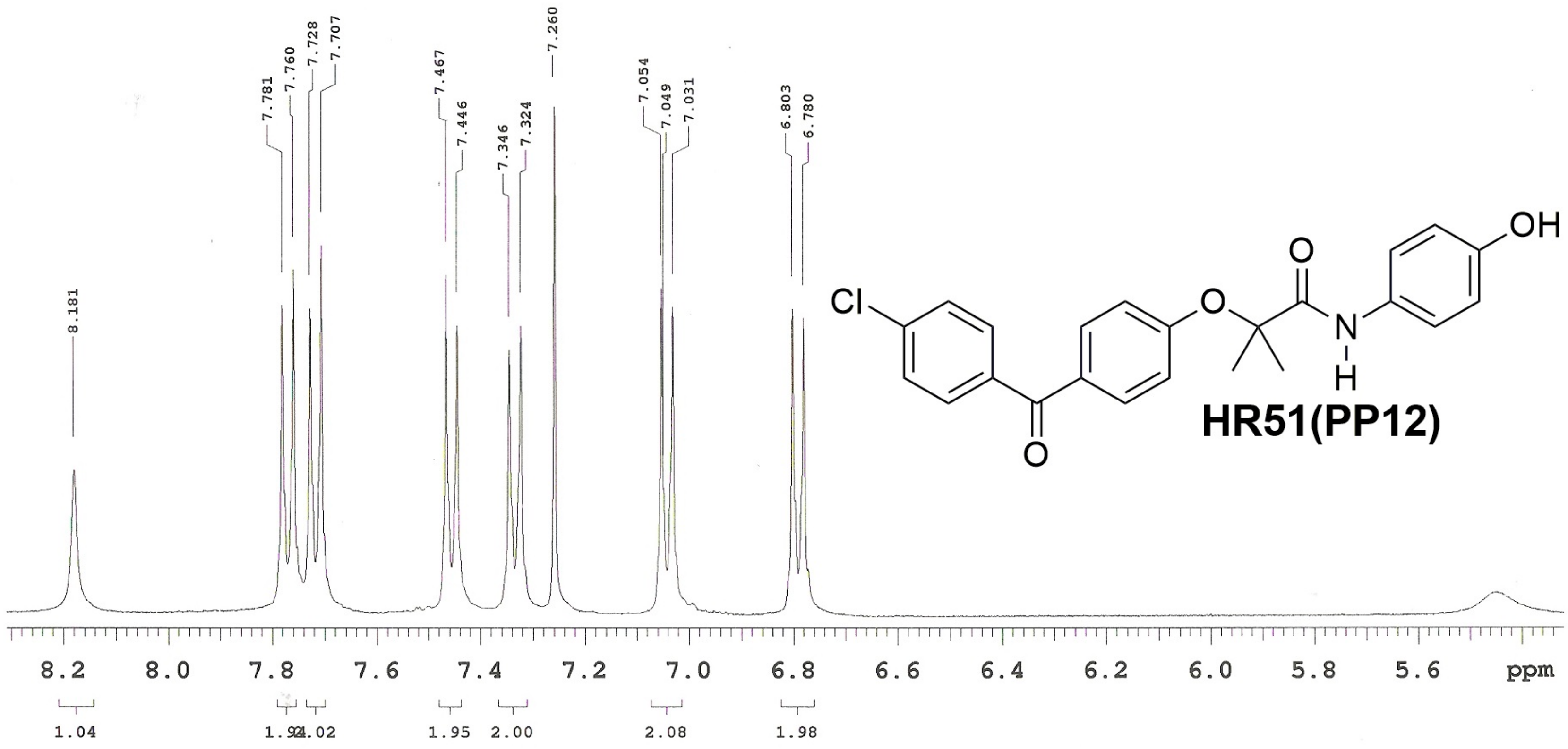

^{1}H-NMR(DMSO-d_6) Varian Mercury 400 Plus

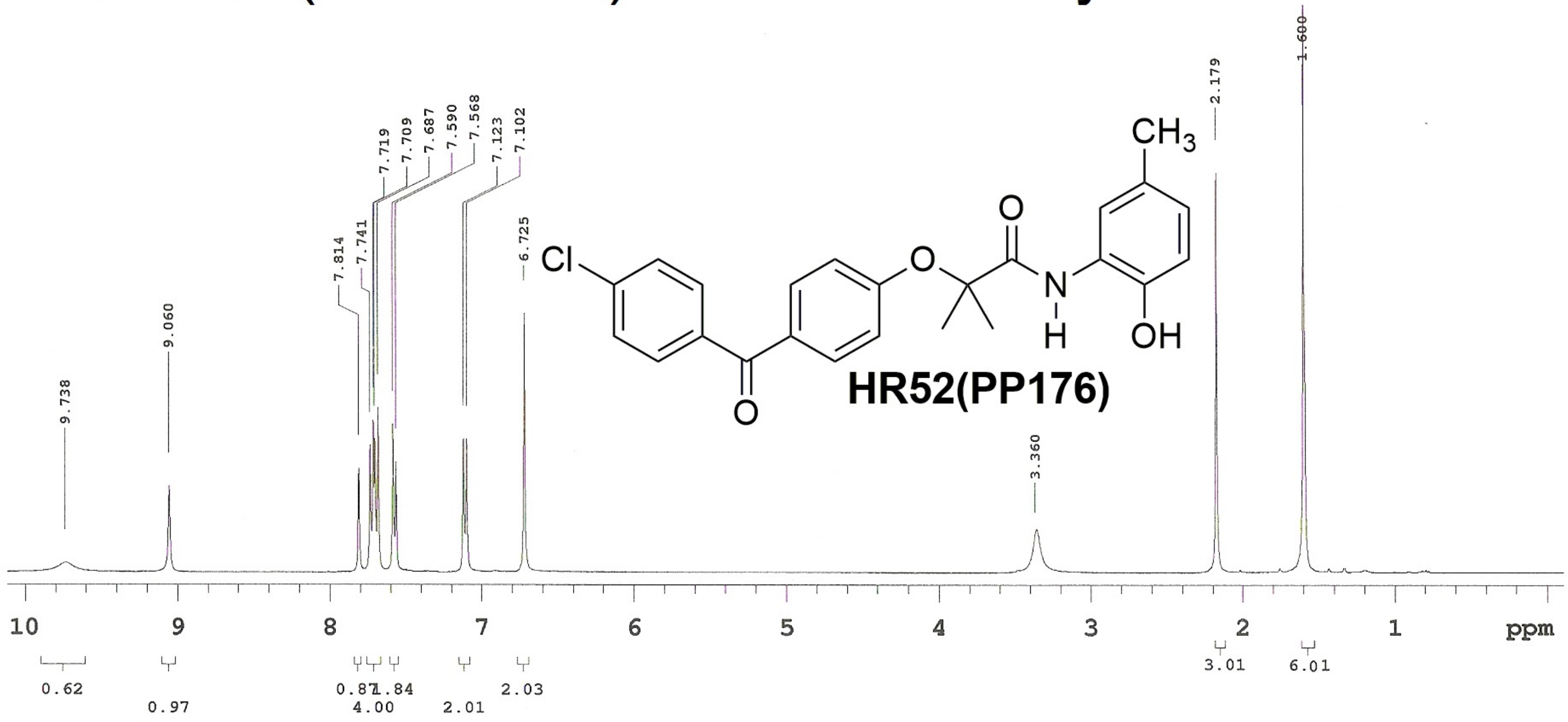

^{1}H-NMR(DMSO-d_6) Varian Mercury 400 Plus

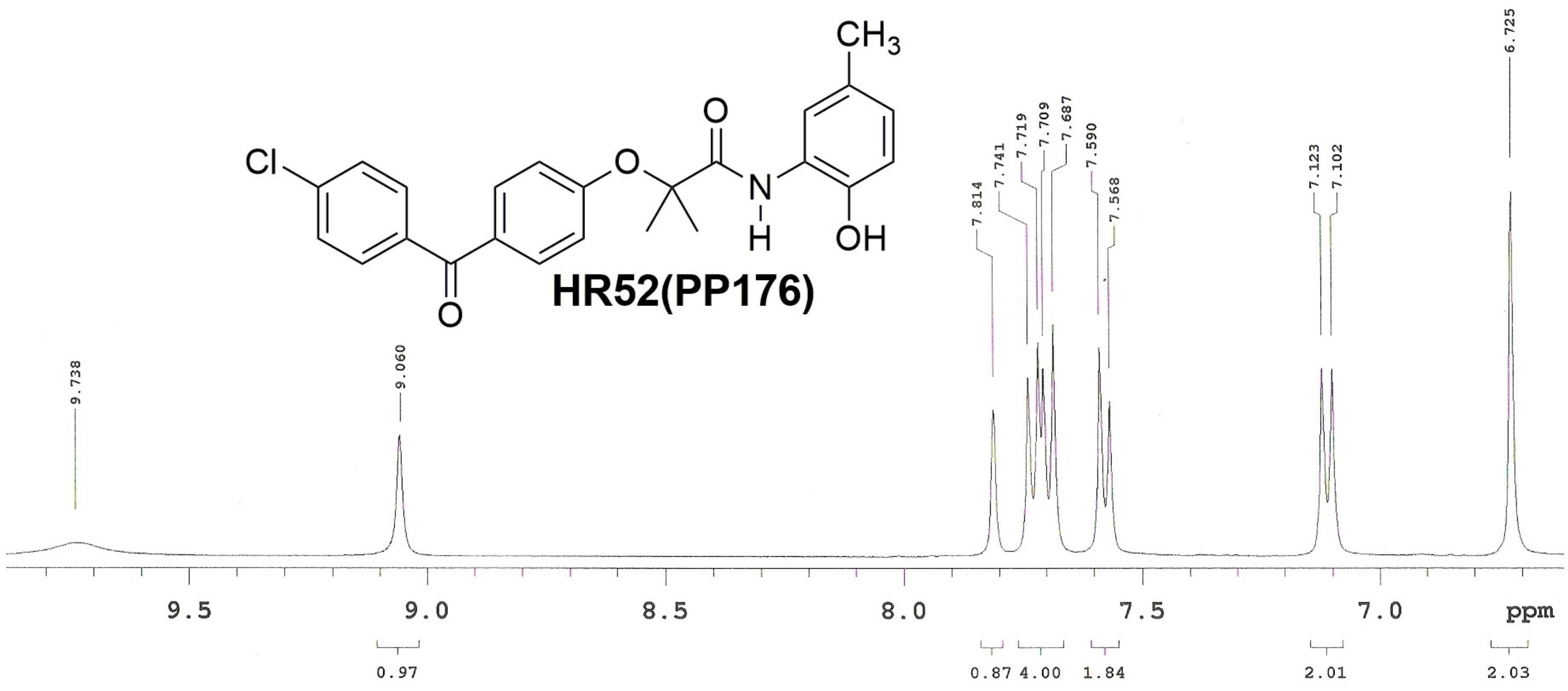

HR52(PP176)

^{13}C-NMR(DMSO-d_6) Varian Mercury 400 plus

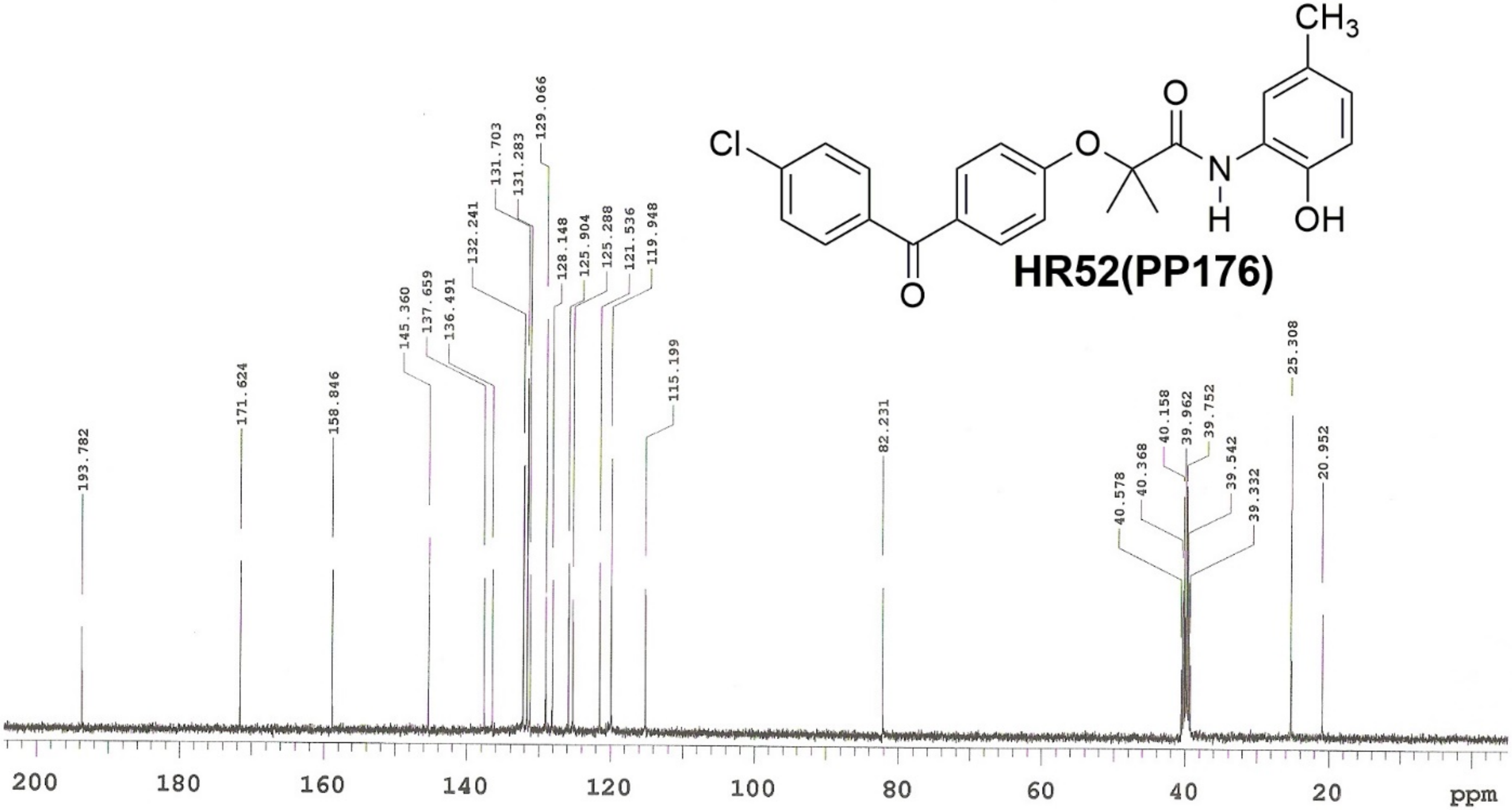

HR52(PP176)

^{1}H-NMR(DMSO-d_6) Varian Mercury 400 Plus

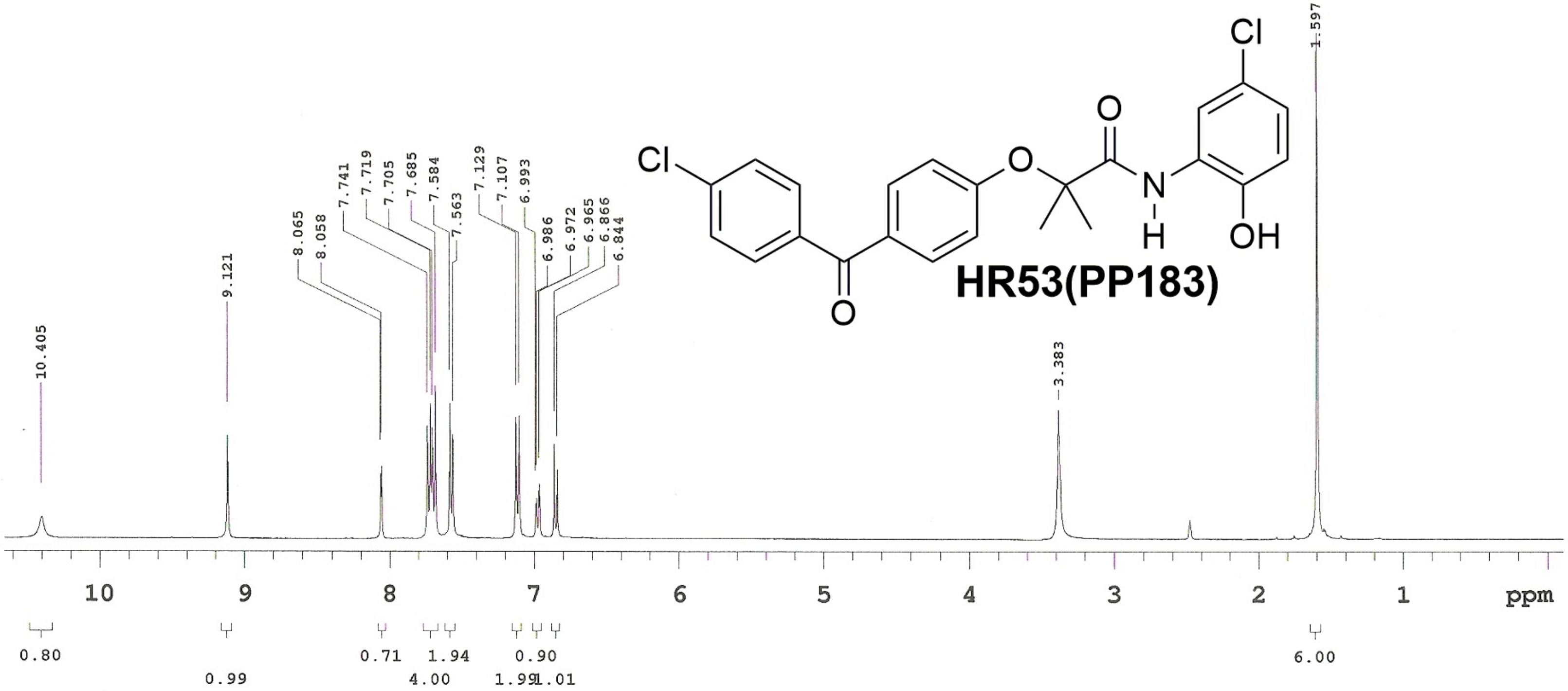

^{1}H-NMR(DMSO-d_6) Varian Mercury 400 Plus

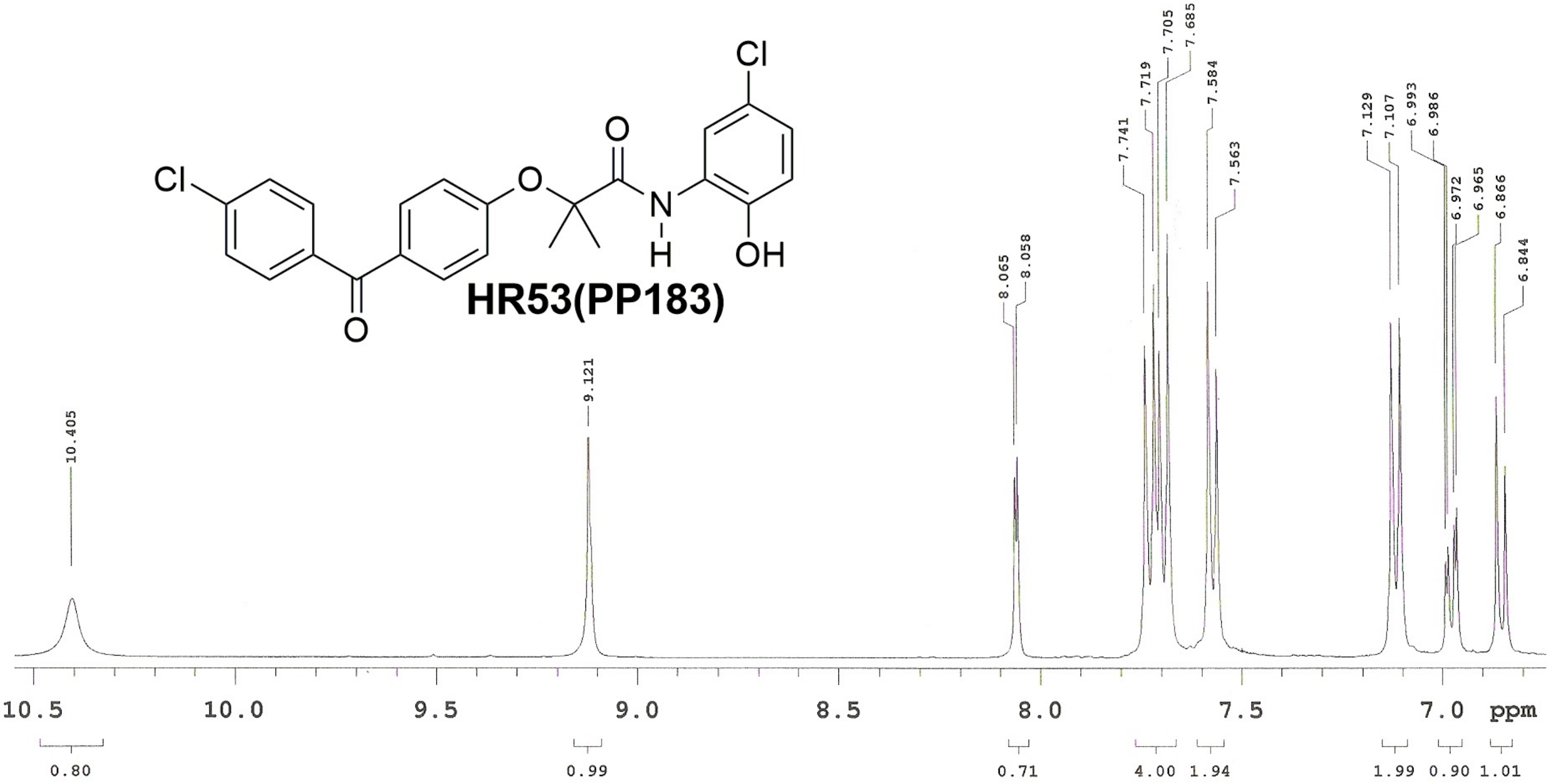

HR53(PP183)

^{13}C-NMR(DMSO-d_6) Varian Mercury 400 plus

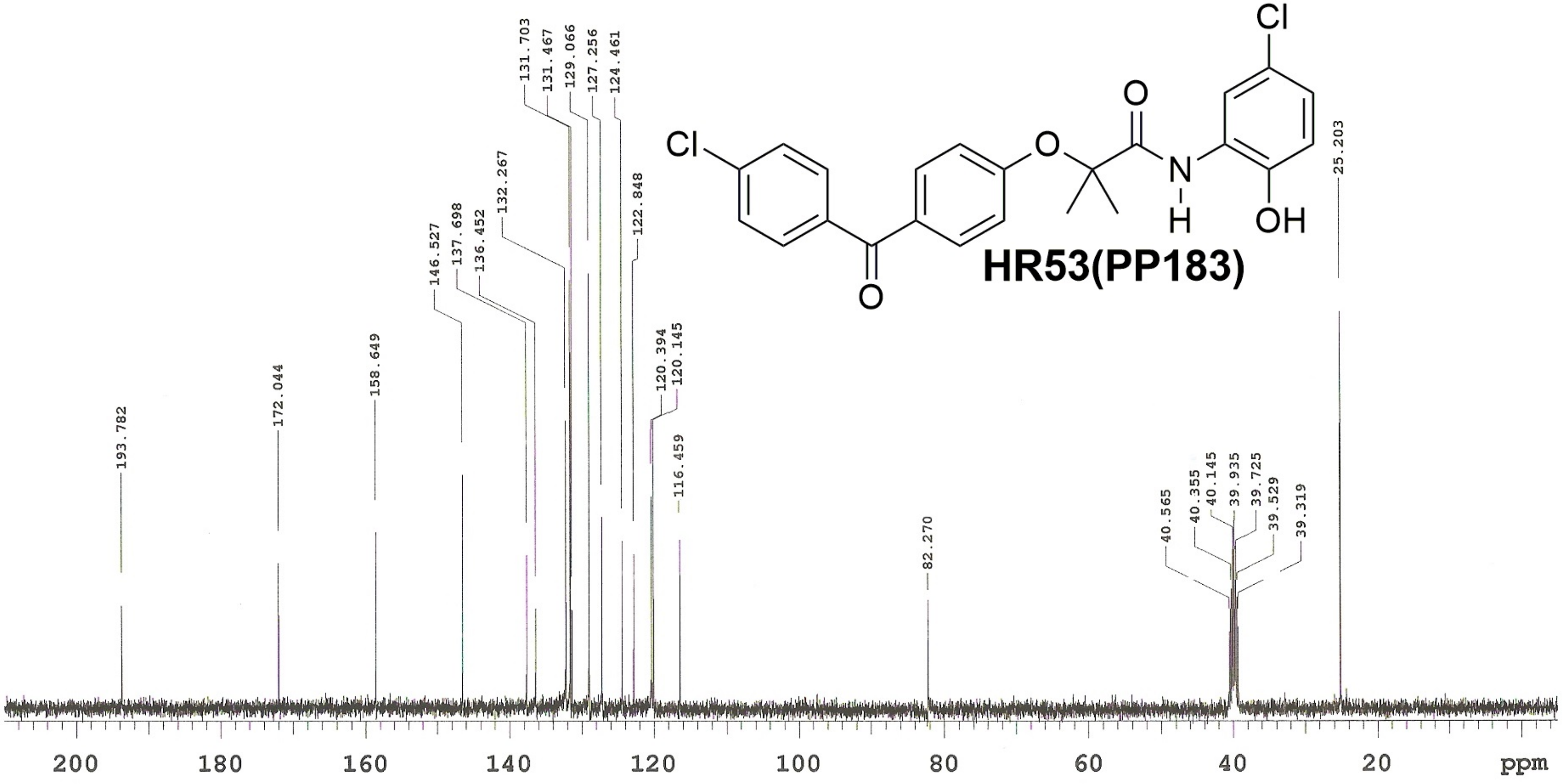

HR53(PP183)

^{1}H-NMR(DMSO-d_6) Varian Mercury 400 Plus

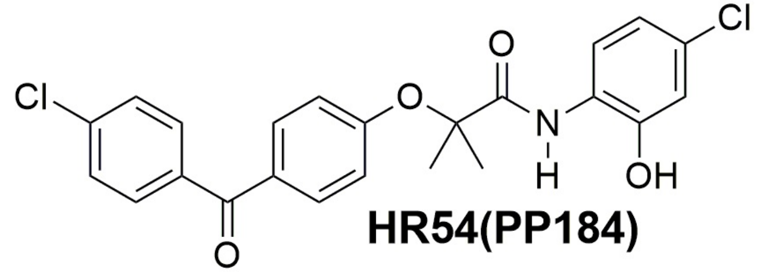

HR54(PP184)

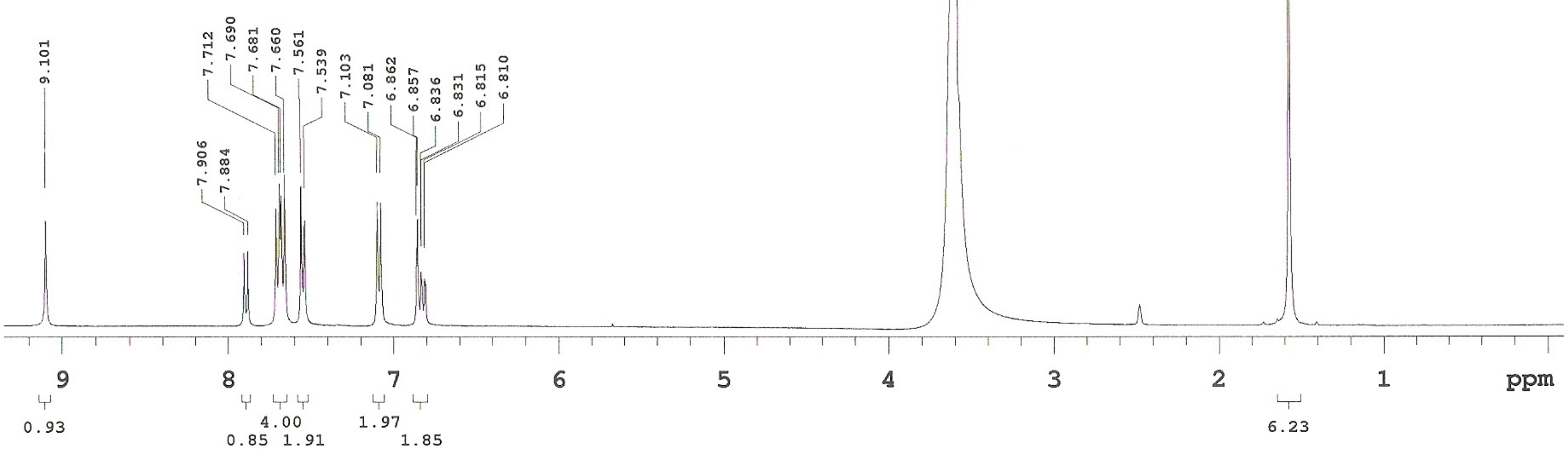

^{1}H-NMR(DMSO-d_6) Varian Mercury 400 Plus

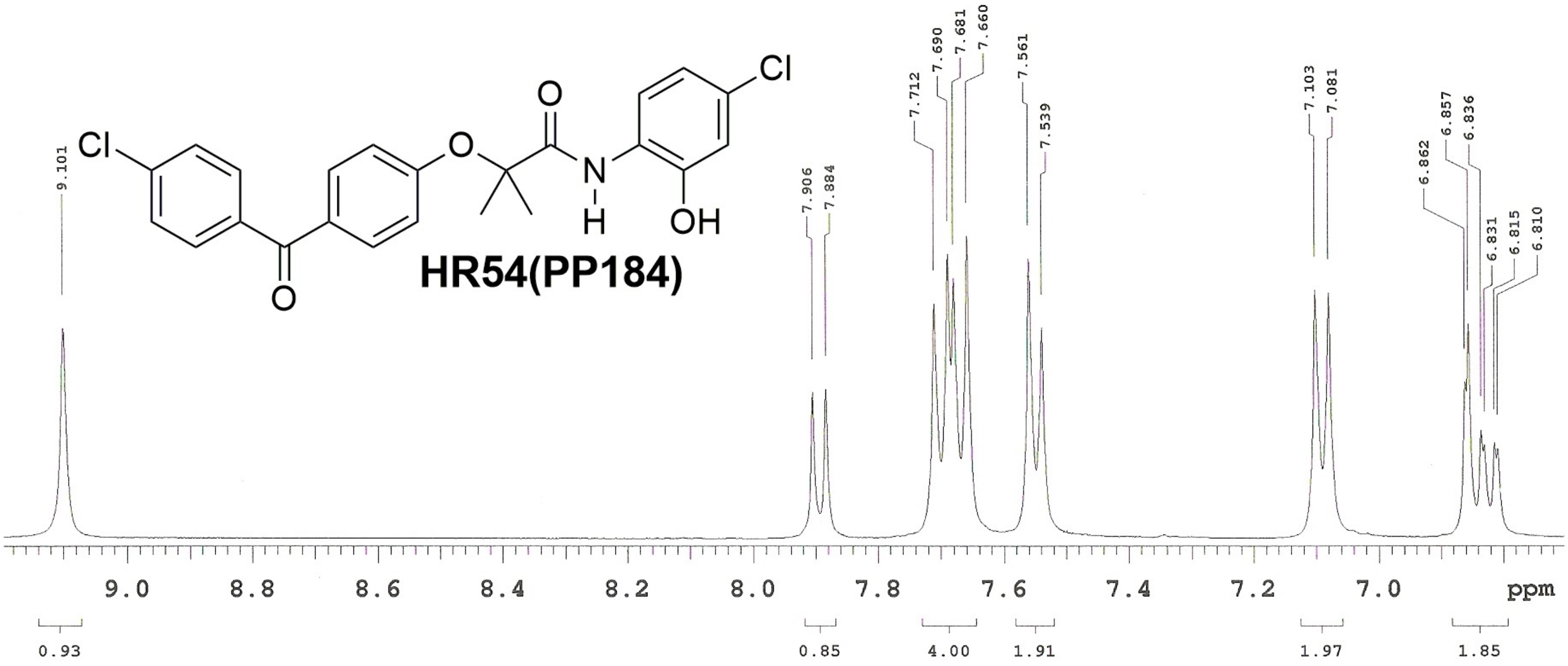

HR54(PP184)

^{13}C-NMR(DMSO-d_6) Varian Mercury 400 plus

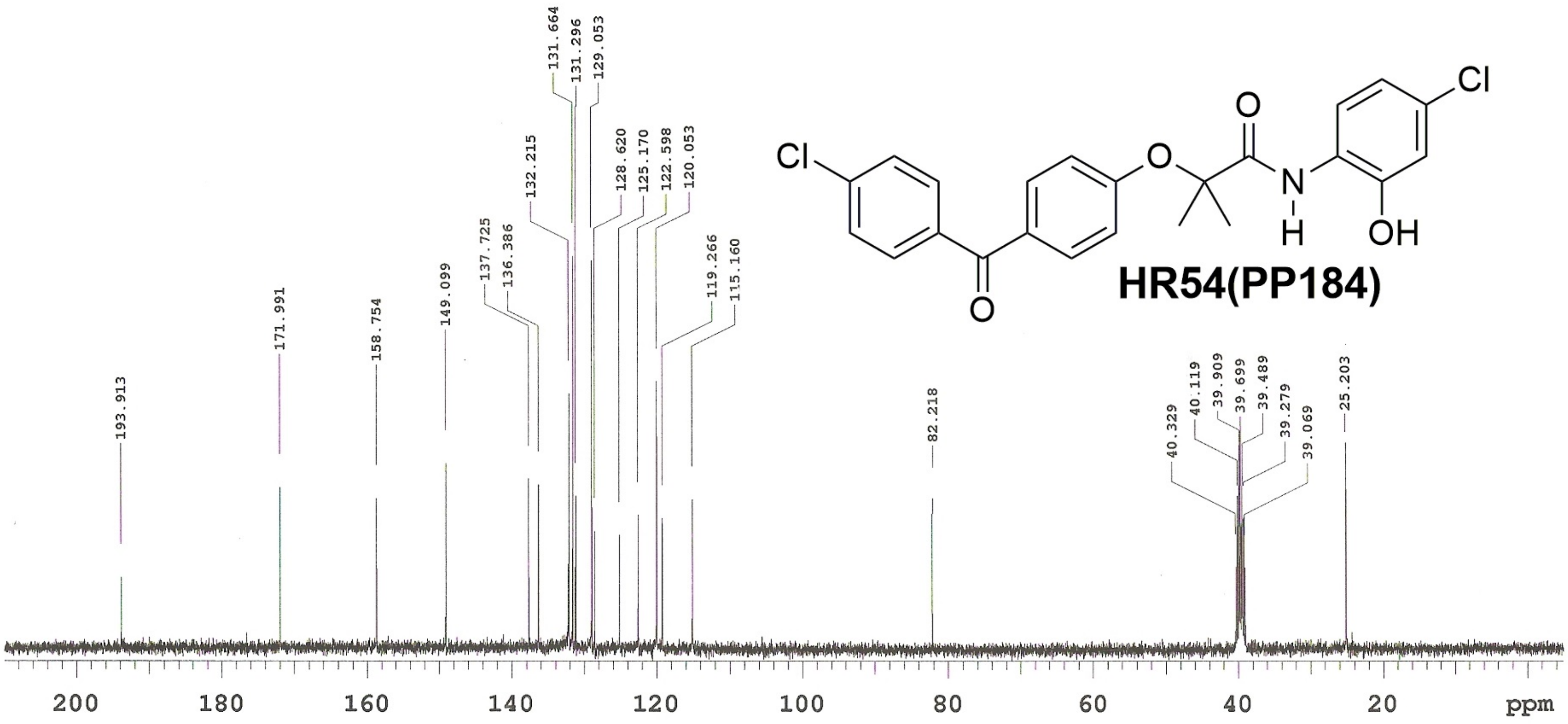

^{1}H-NMR(DMSO-d_6) Varian Mercury 400 Plus

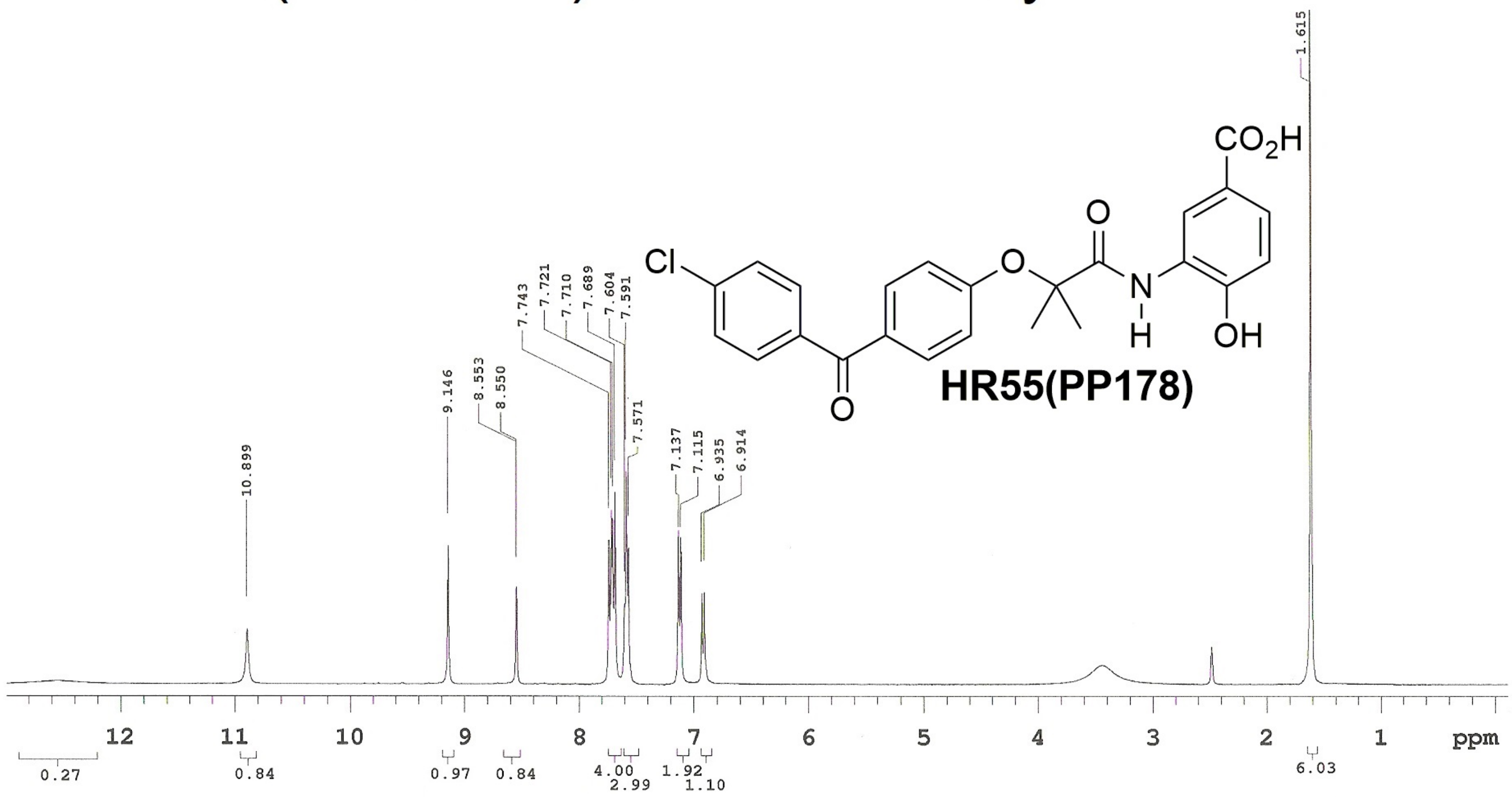

HR55(PP178)

^1H-NMR(DMSO-d_6) Varian Mercury 400 Plus

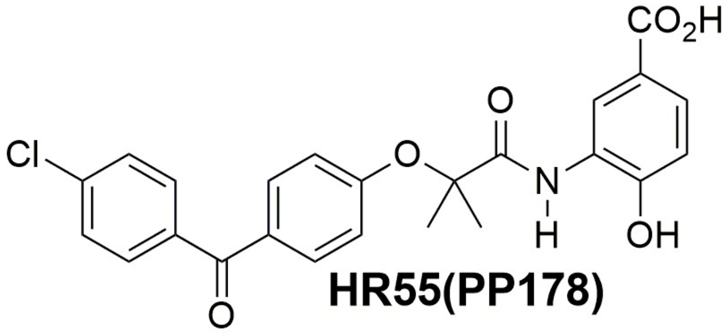

HR55(PP178)

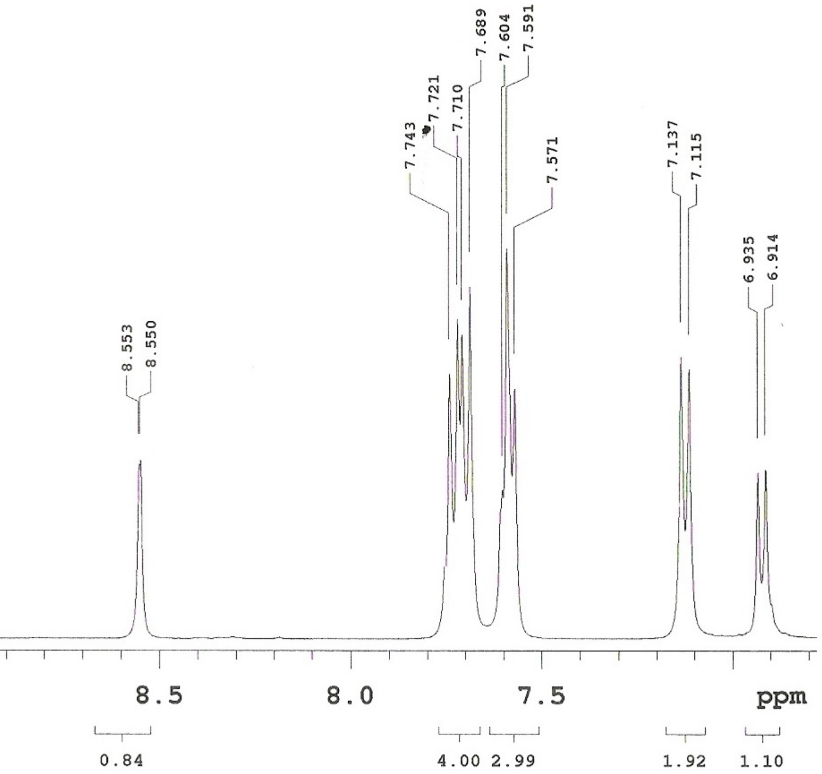

^{13}C-NMR($DMSO-d_6$) Varian Mercury 400 plus

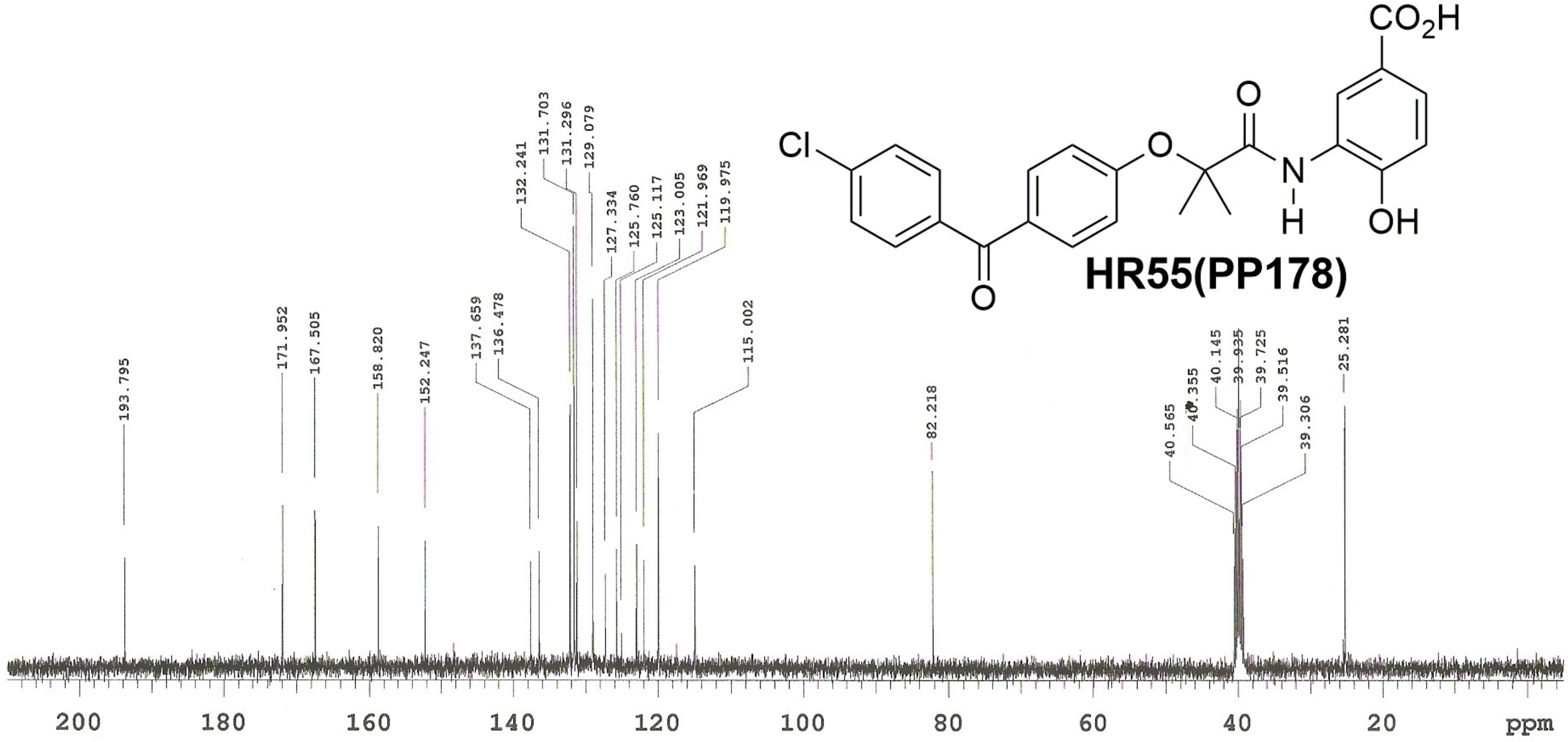

^{1}H-NMR($CDCl_3$) Varian Mercury 400 Plus

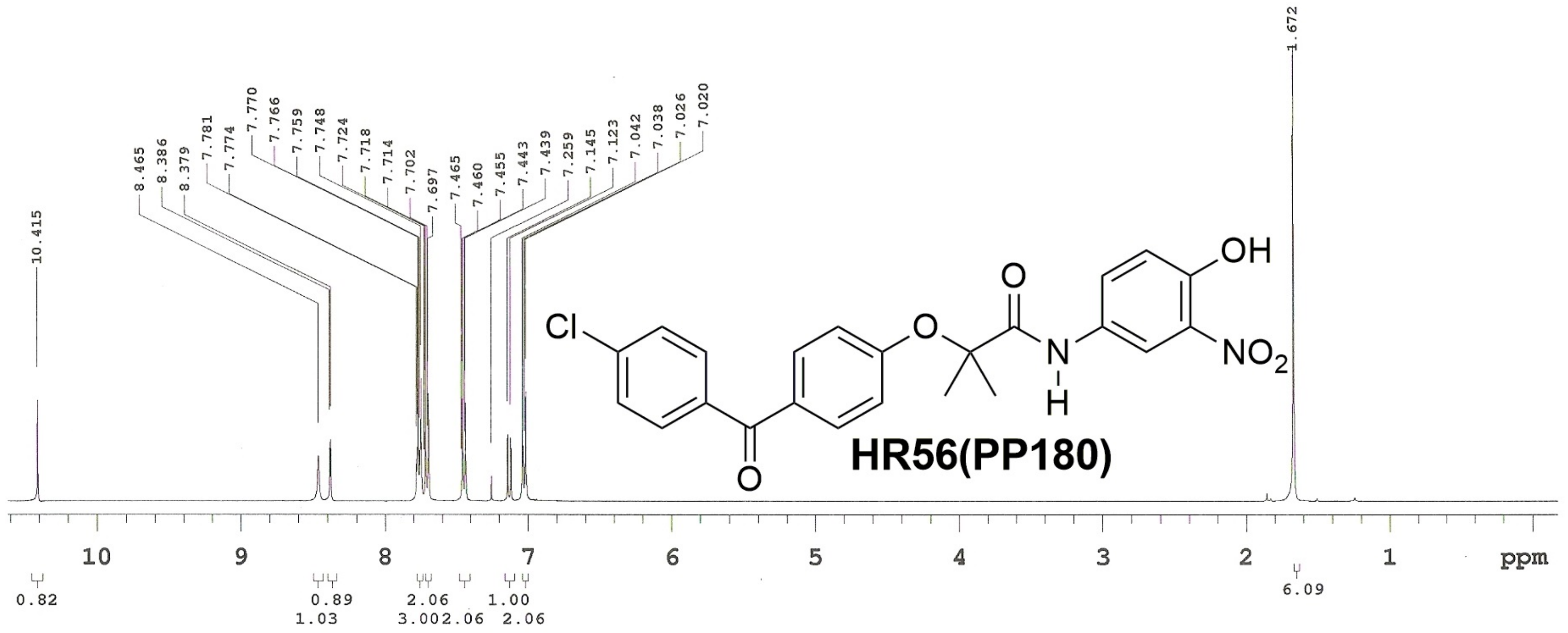

HR56(PP180)

^{1}H-NMR($CDCl_3$) Varian Mercury 400 Plus

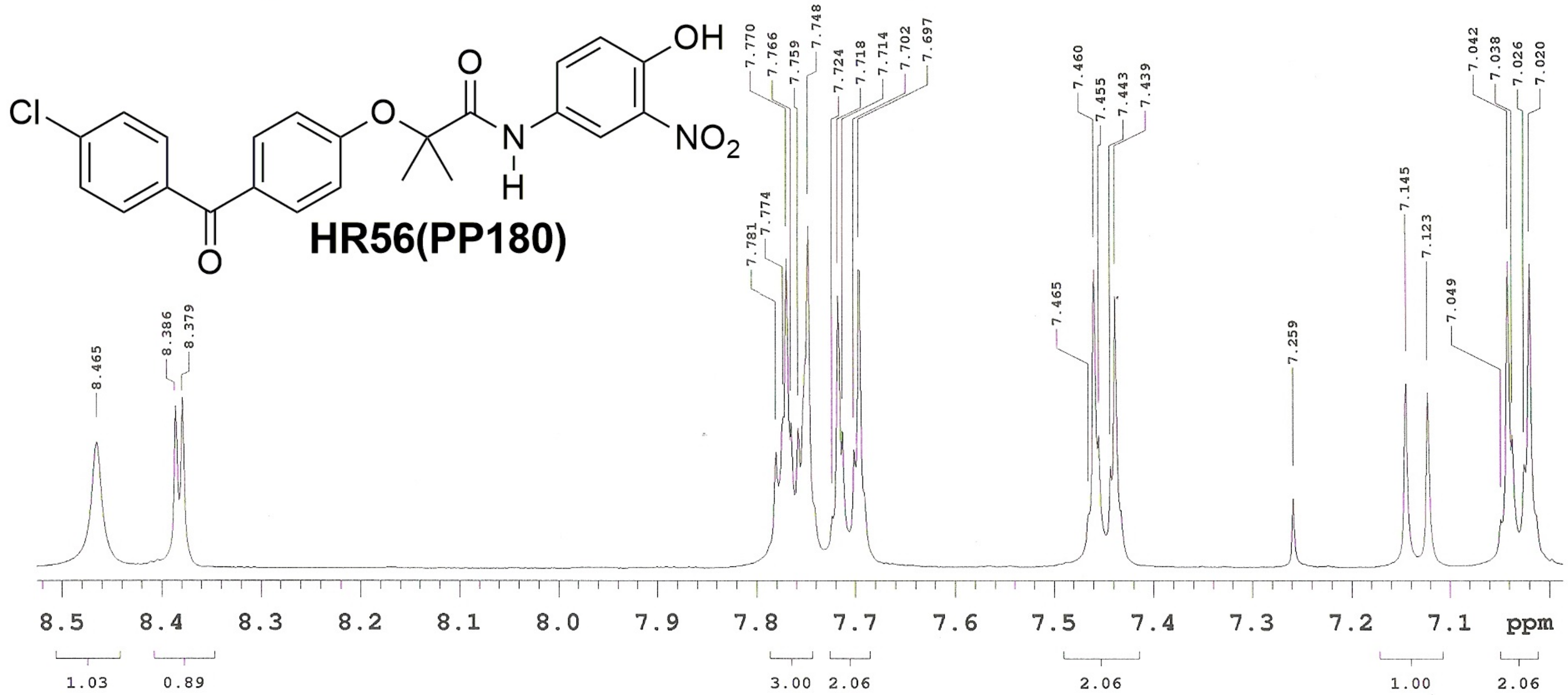

HR56(PP180)

^{13}C-NMR($CDCl_3$) Varian Mercury 400 Plus

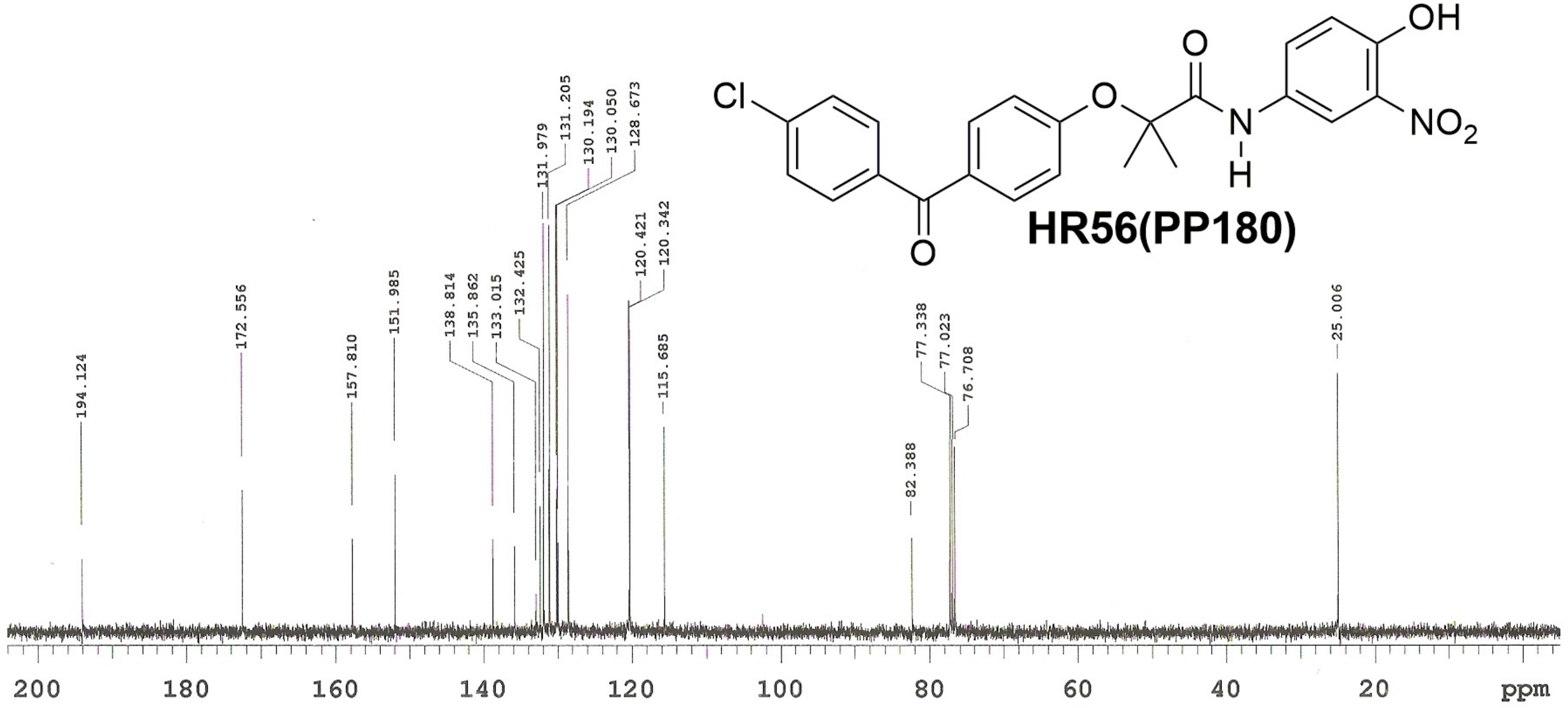

HR56(PP180)

^{1}H-NMR ($CDCl_3$) Varian Mercury 400 Plus

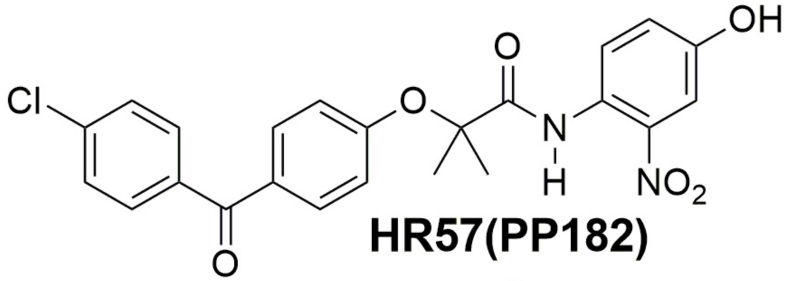

HR57(PP182)

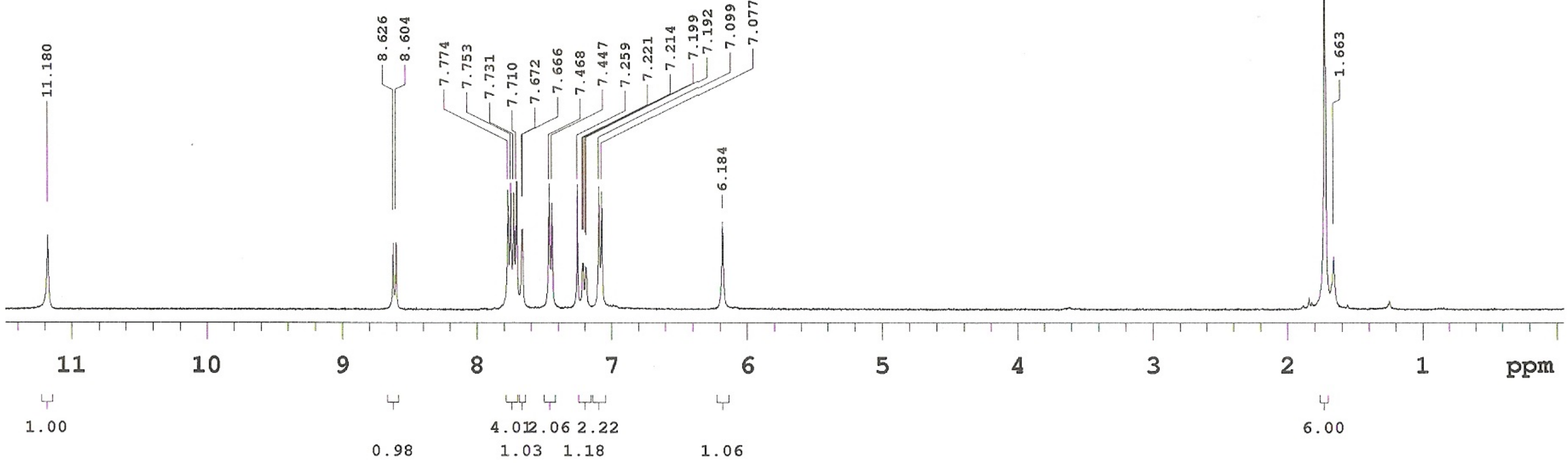

^{1}H-NMR ($CDCl_3$) Varian Mercury 400 Plus

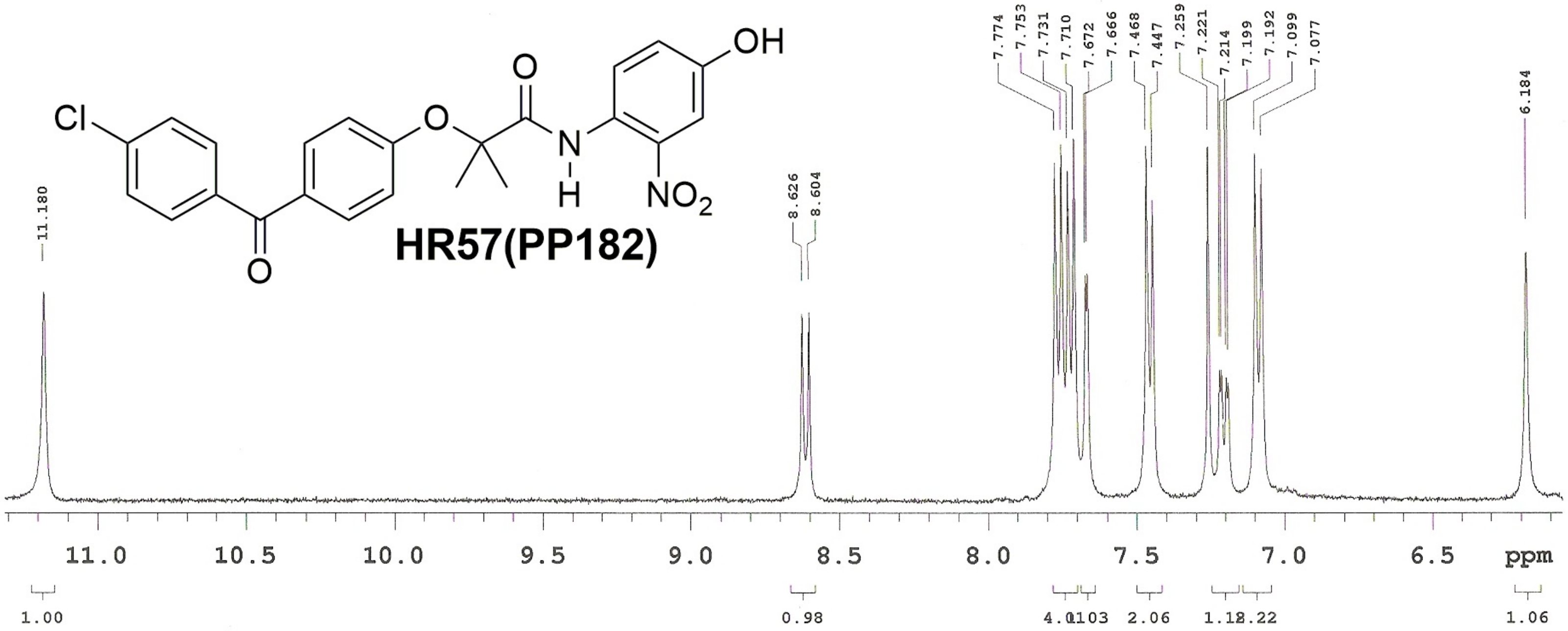

HR57(PP182)

^{13}C-NMR($CDCl_3$) Varian Mercury 400 Plus

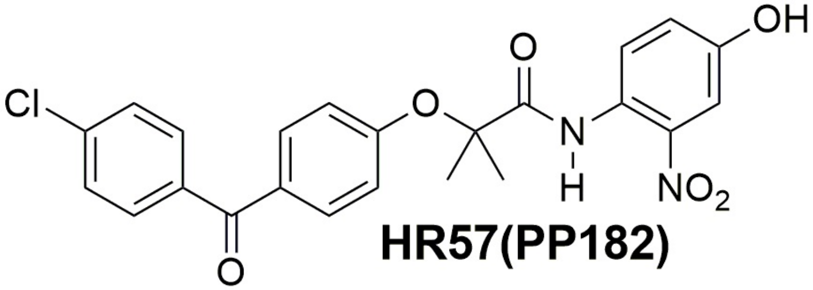

HR57(PP182)

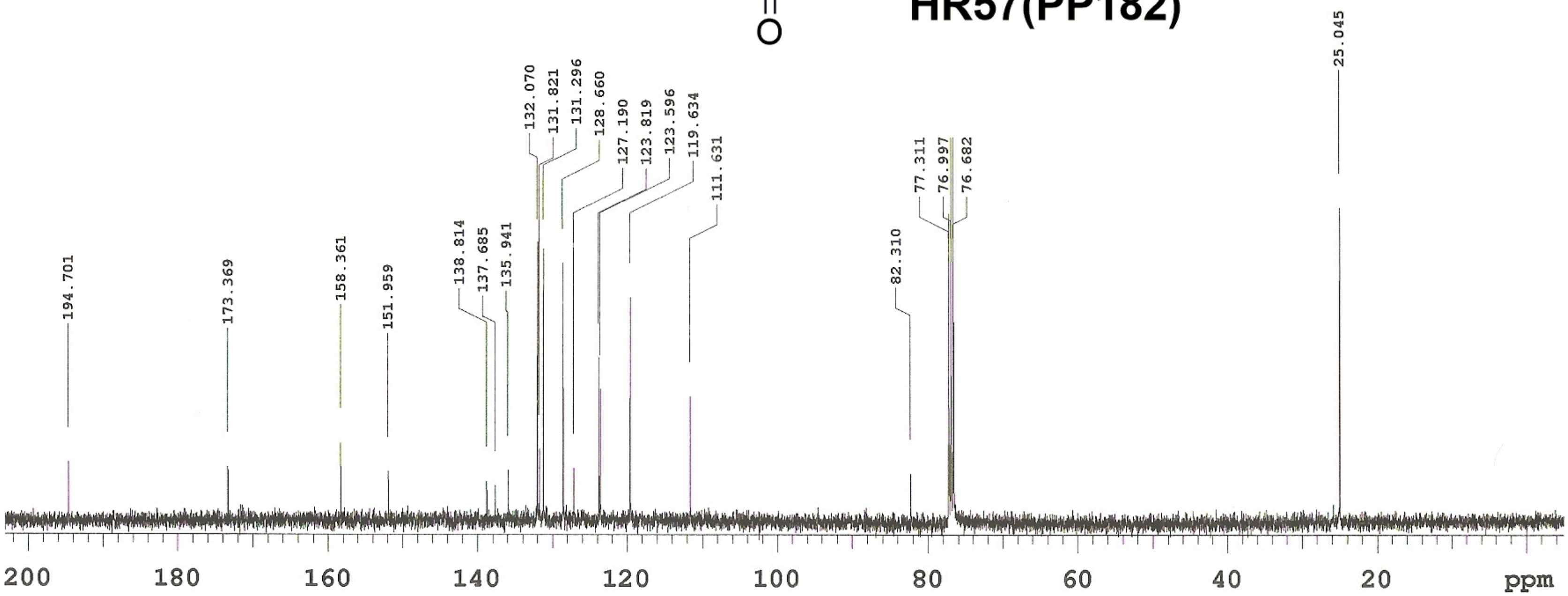

^{1}H-NMR(DMSO-d_6) Varian Mercury 400 Plus

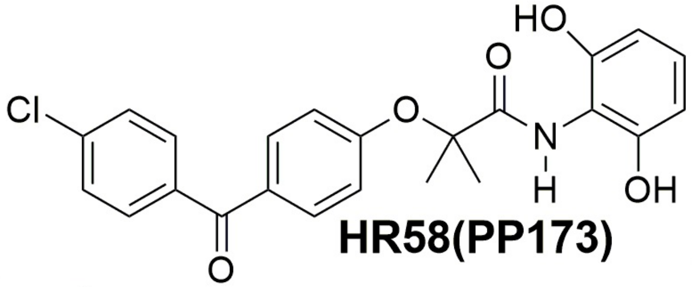

HR58(PP173)

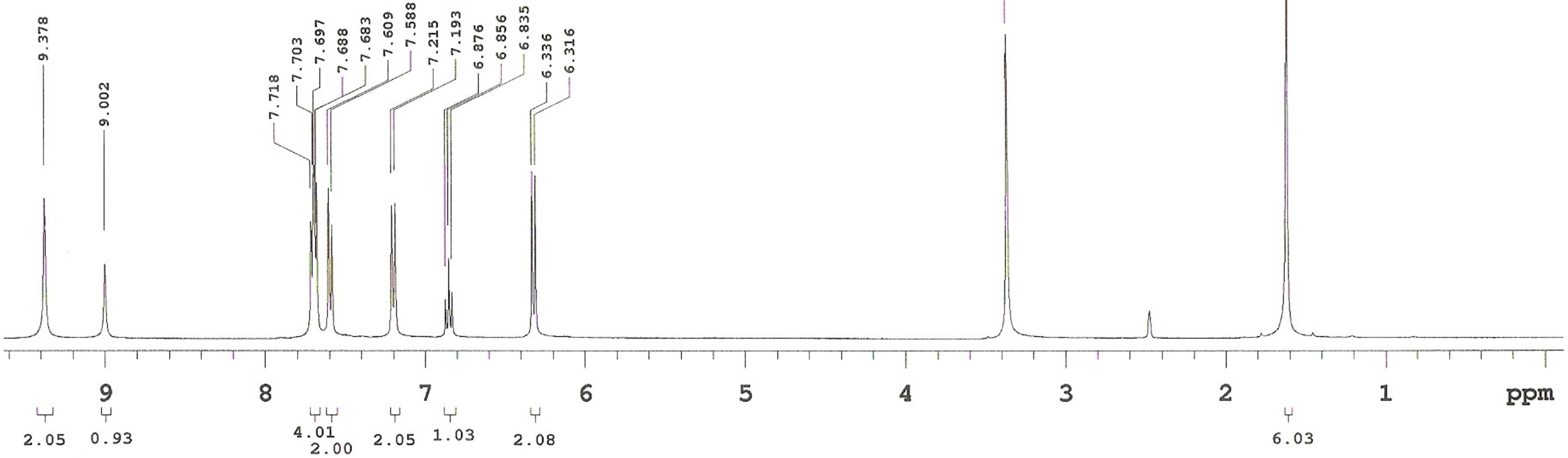

^{1}H-NMR(DMSO-d_6) Varian Mercury 400 Plus

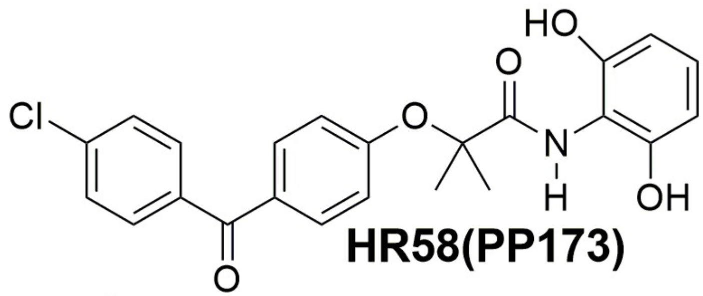

HR58(PP173)

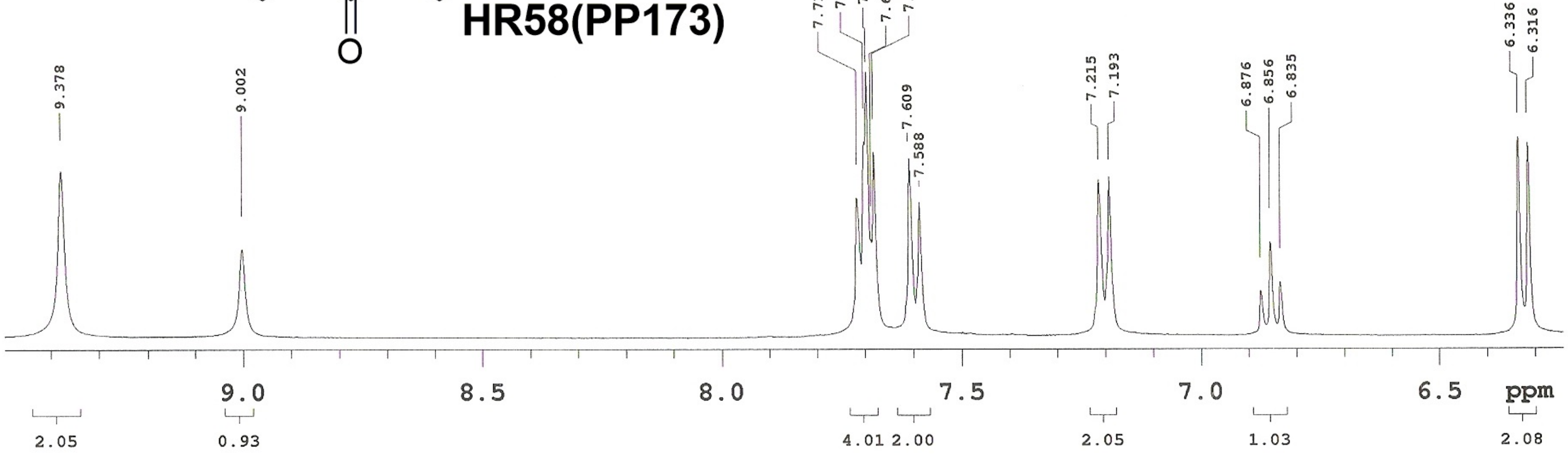

^{13}C-NMR(DMSO-d_6) Varian Mercury 400 plus

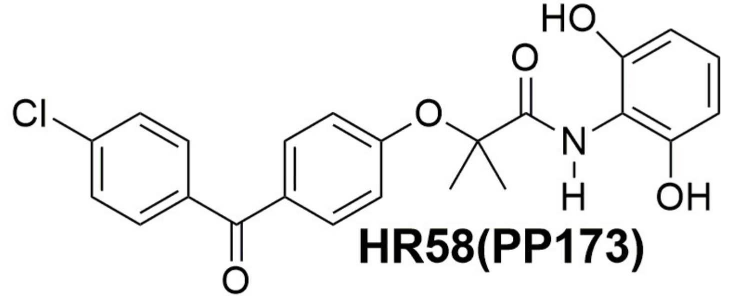

HR58(PP173)

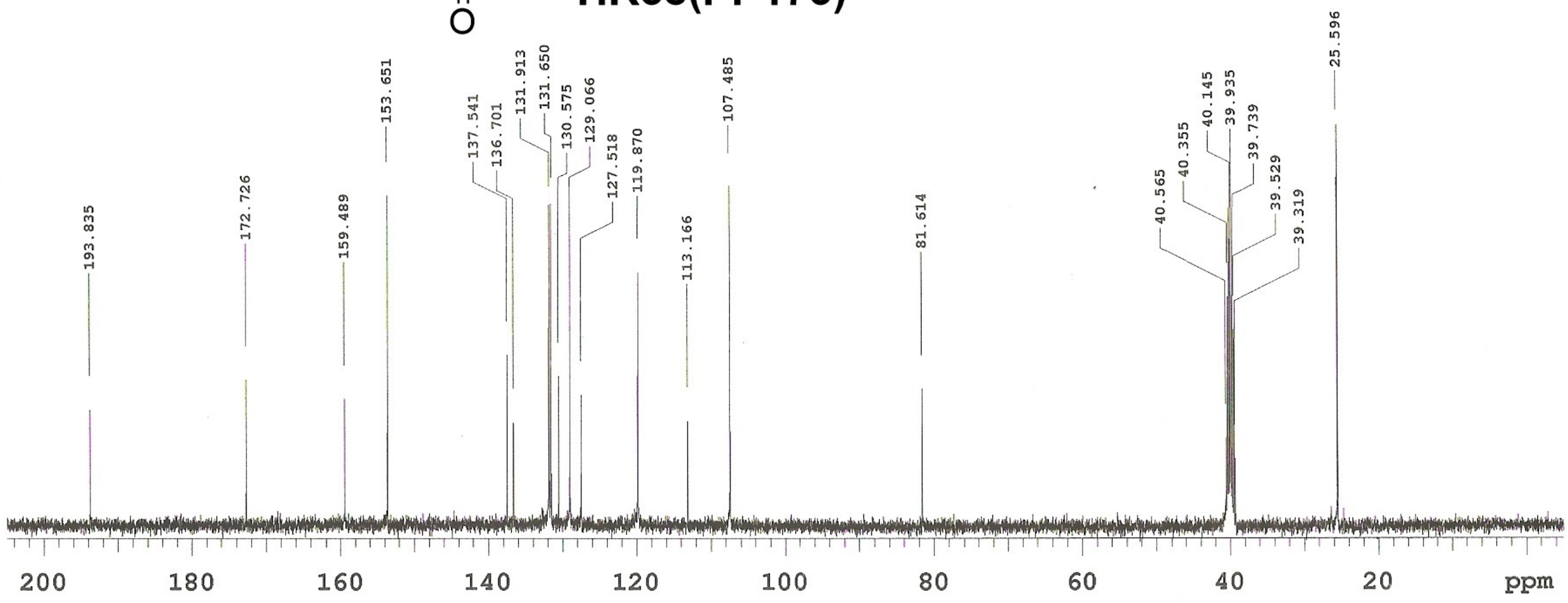

^{1}H-NMR(DMSO-d_6) Varian Mercury 400 Plus

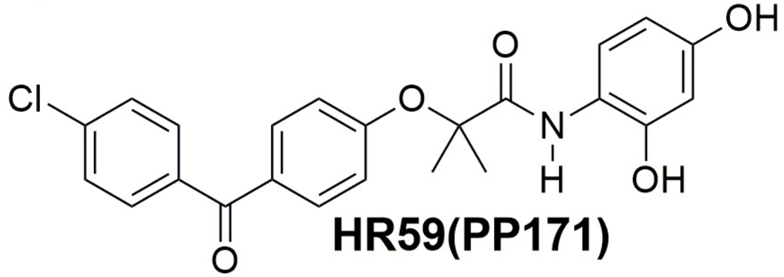

HR59(PP171)

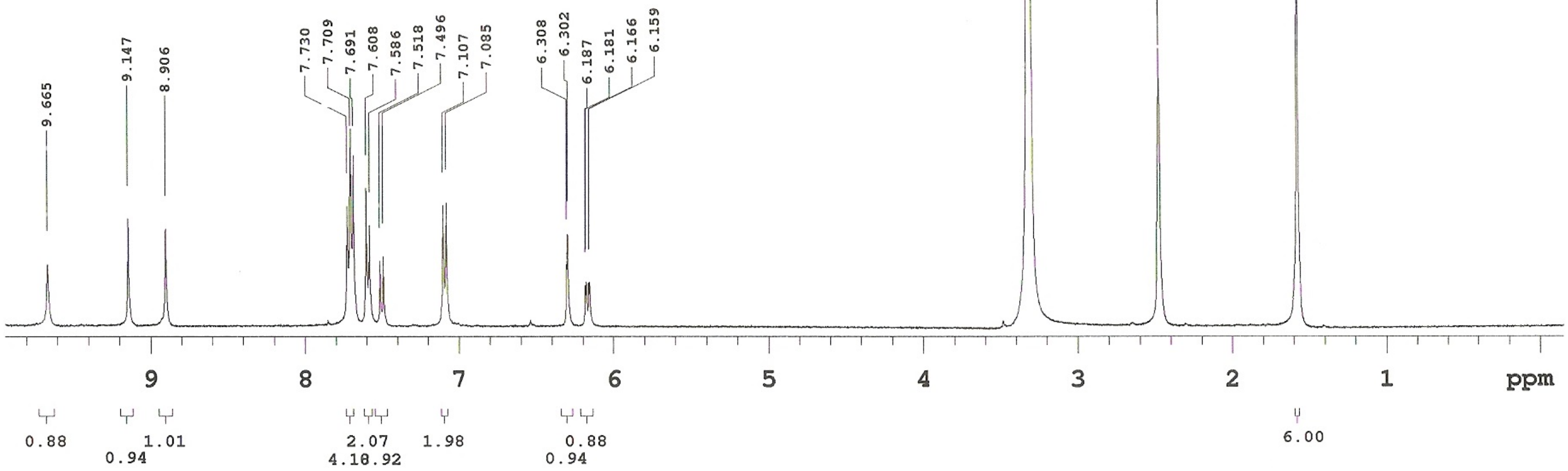

^{1}H-NMR(DMSO-d_6) Varian Mercury 400 Plus

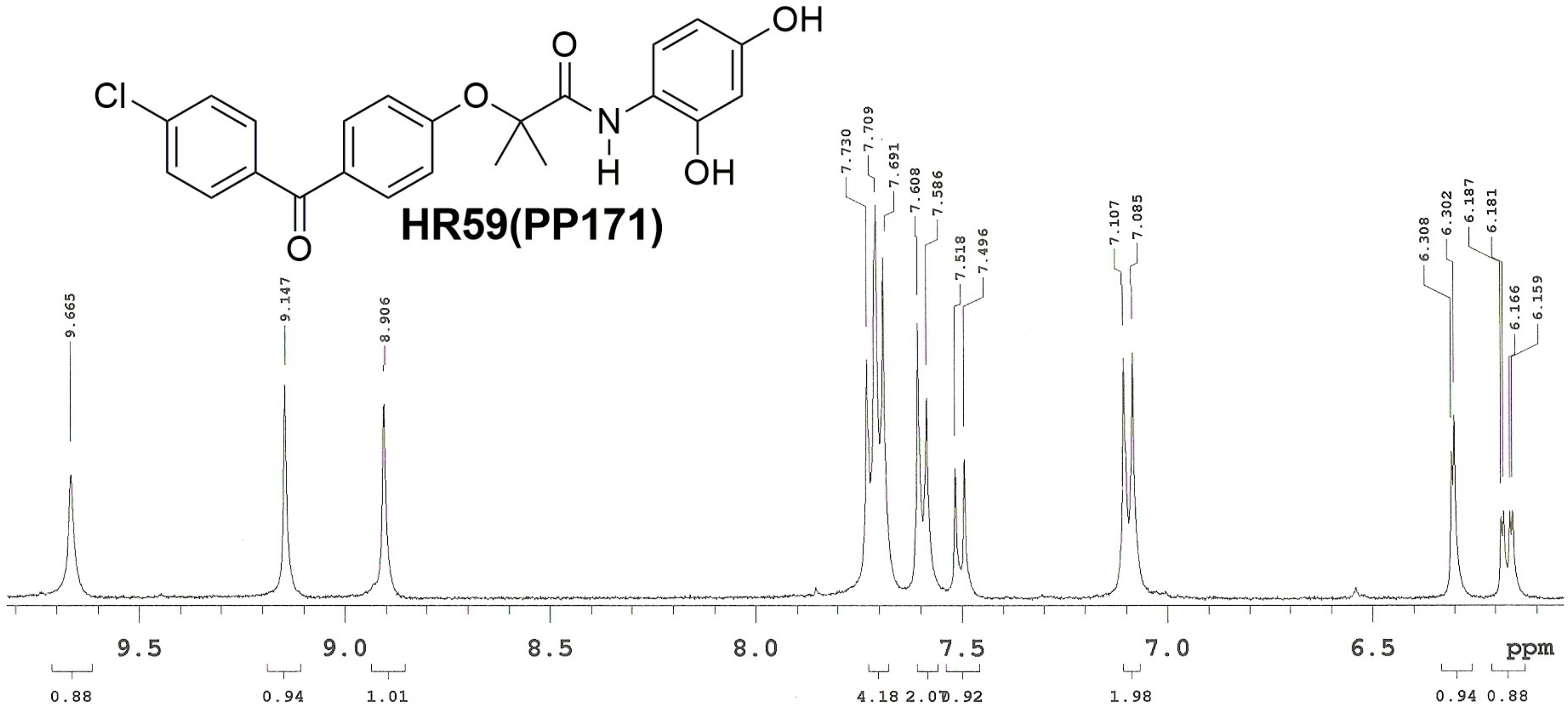

b

^{1}H-NMR($CDCl_3$) Varian Mercury 400 Plus

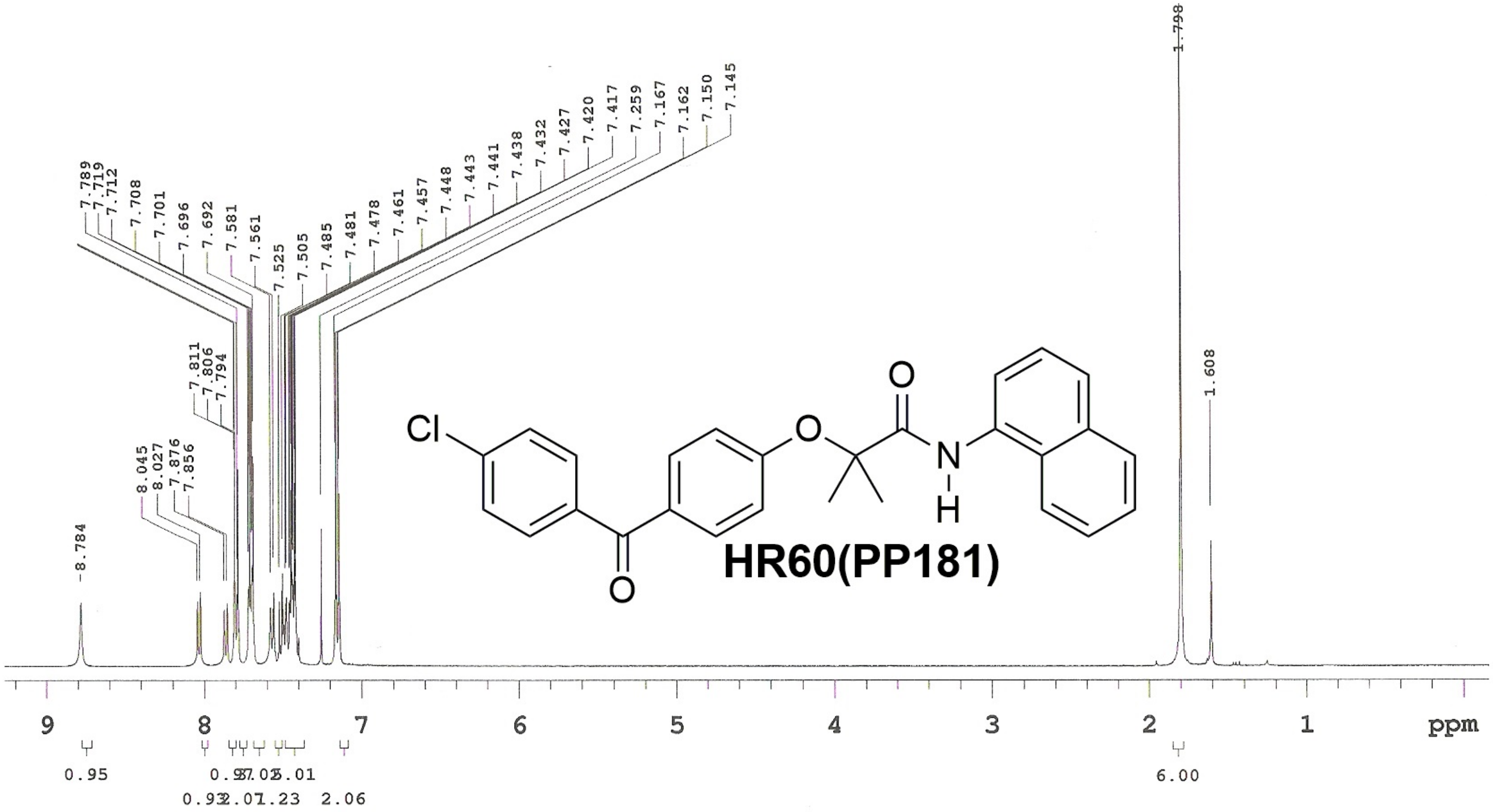

HR60(PP181)

^1H-NMR($CDCl_3$) Varian Mercury 400 Plus

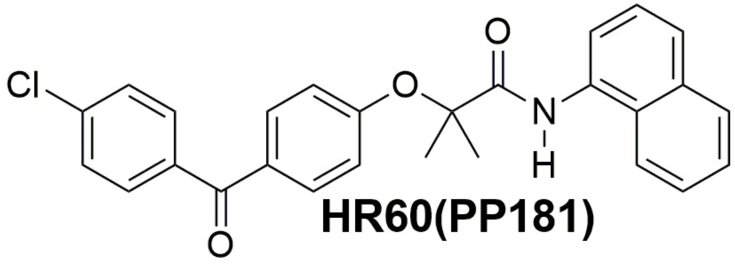

HR60(PP181)

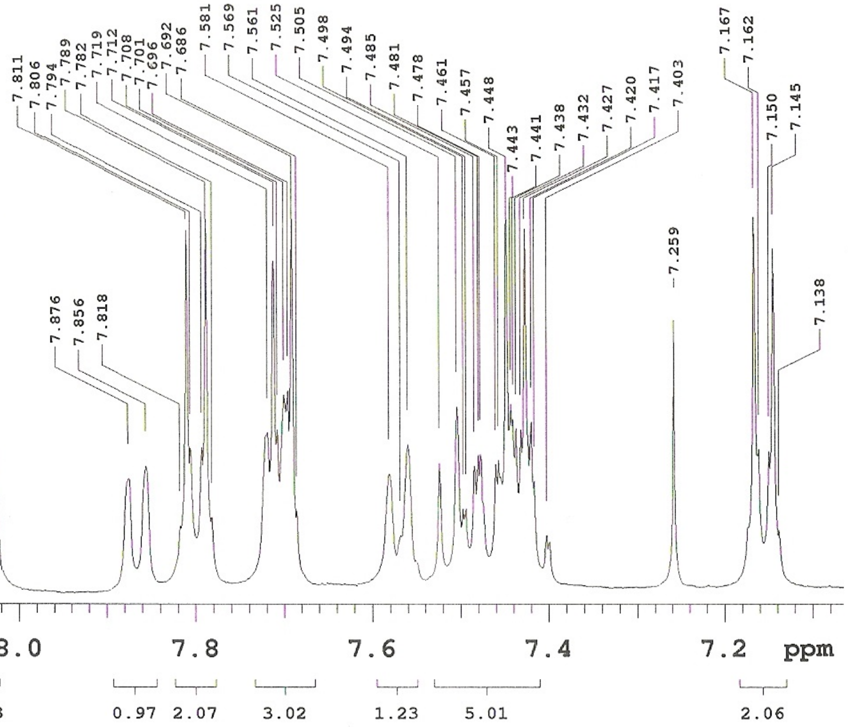

^{13}C-NMR($CDCl_3$) Varian Mercury 400 Plus

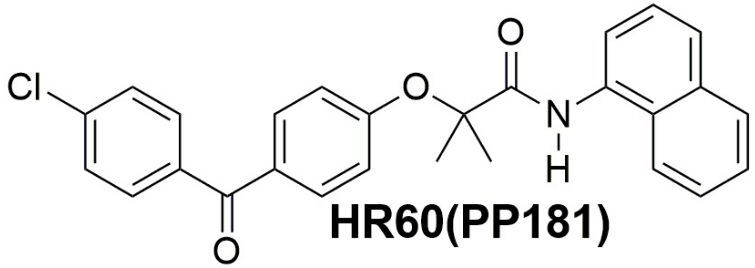

HR60(PP181)

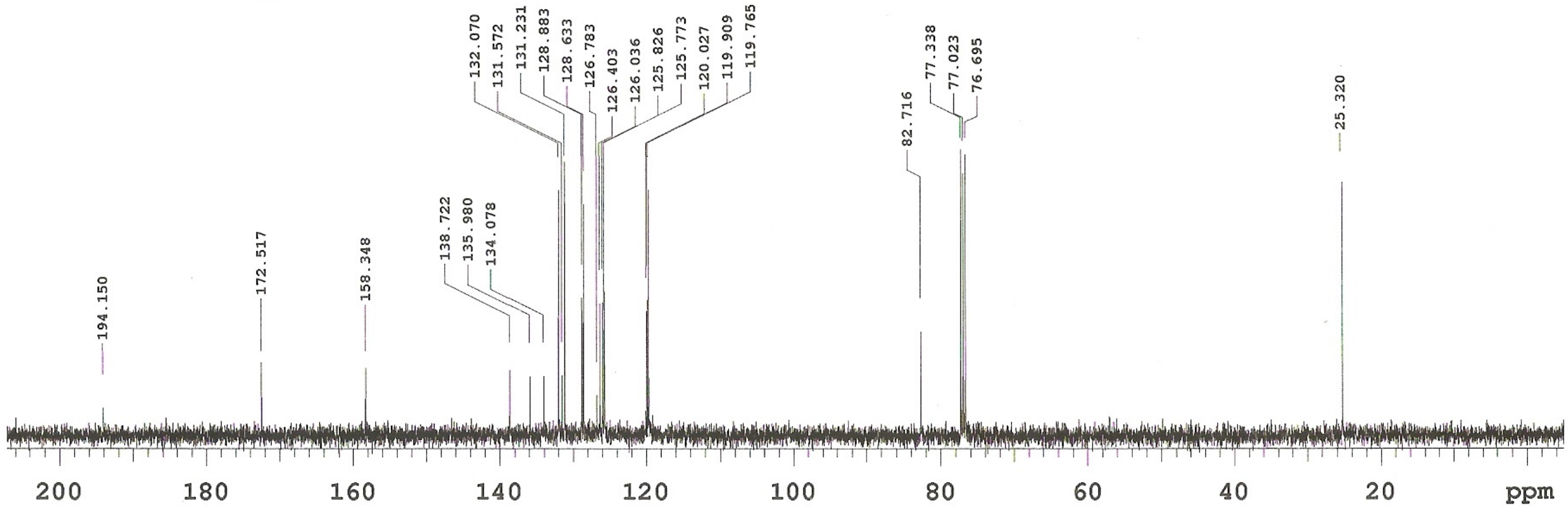

^{1}H-NMR(DMSO-d_6) Varian Mercury 400 Plus

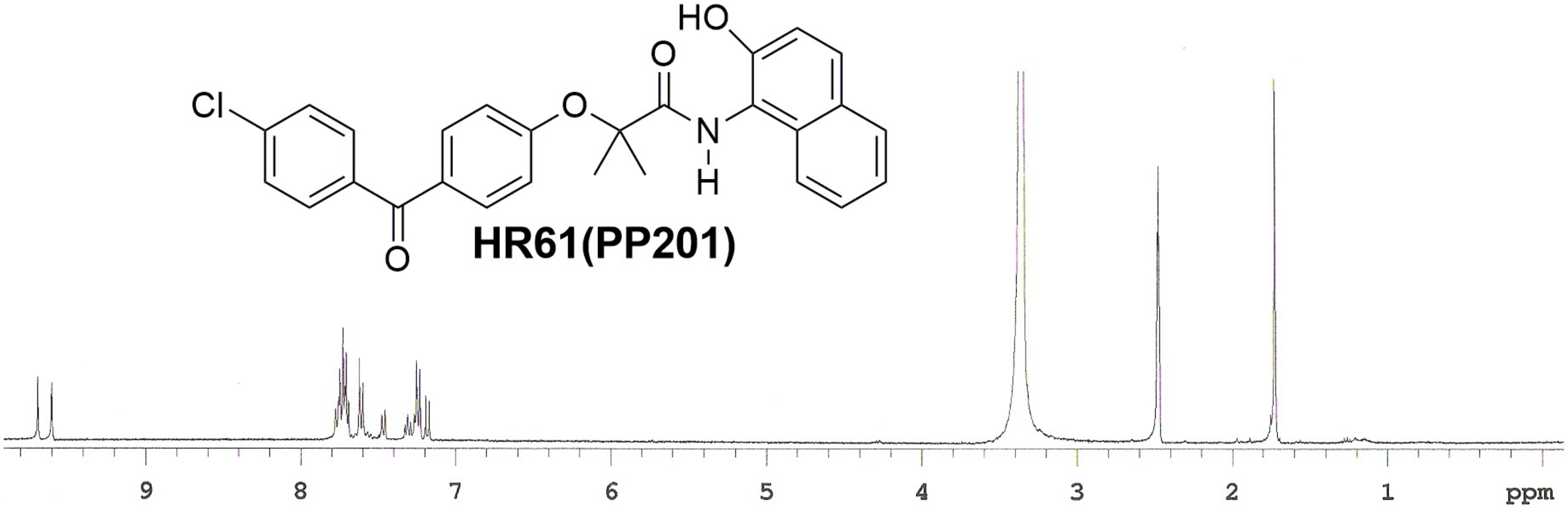

HR61(PP201)

^{13}C-NMR(DMSO-d_6) Varian Mercury 400 plus

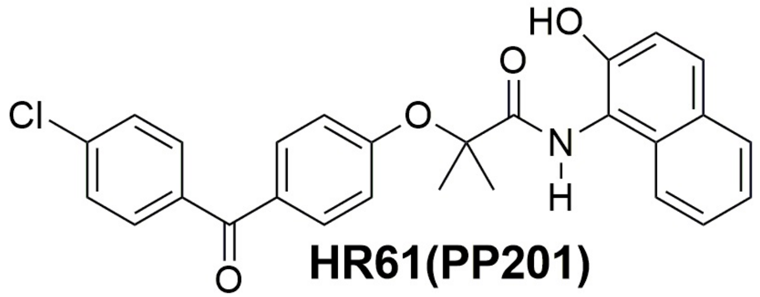

HR61(PP201)

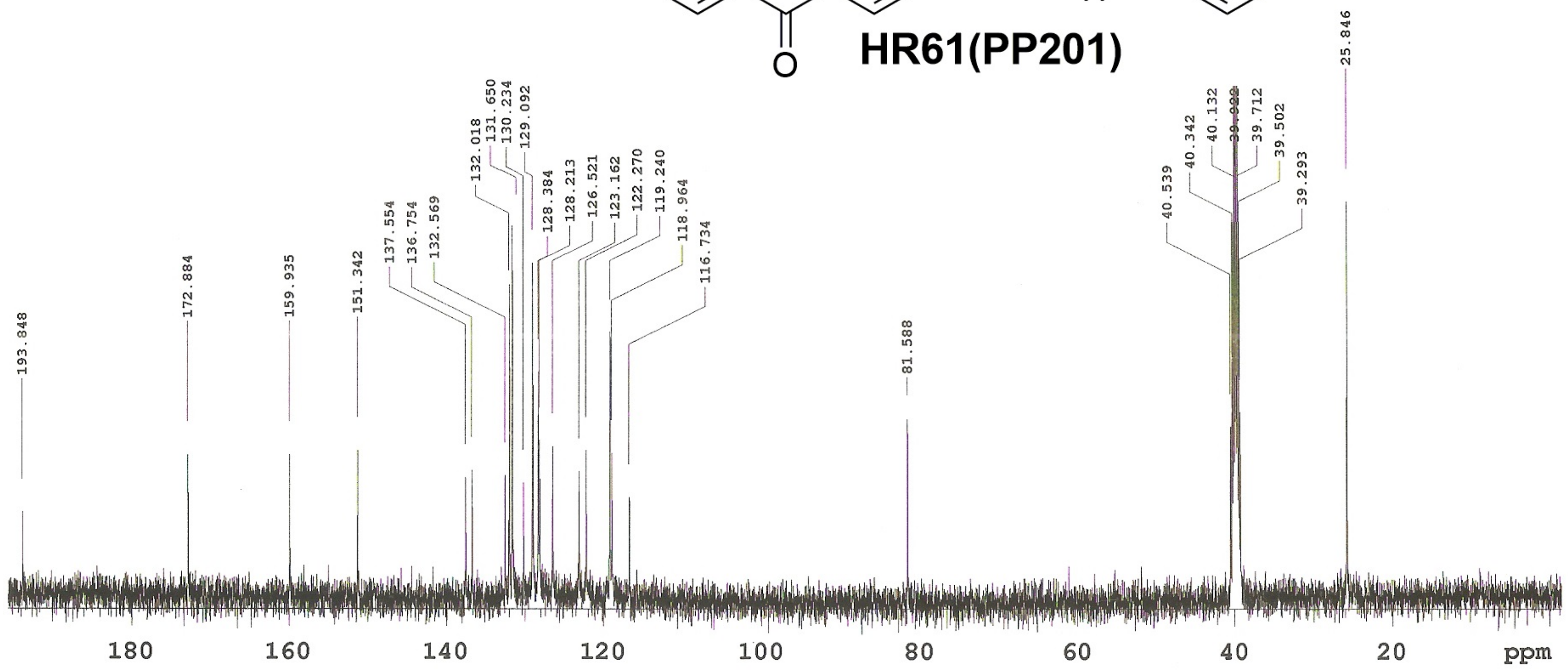

^{1}H-NMR(DMSO-d_6) Varian Mercury 400 Plus

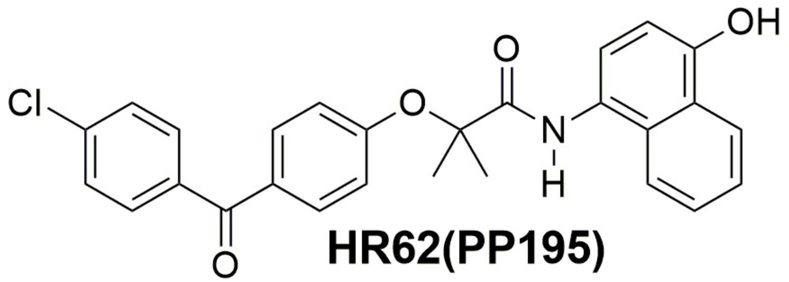

HR62(PP195)

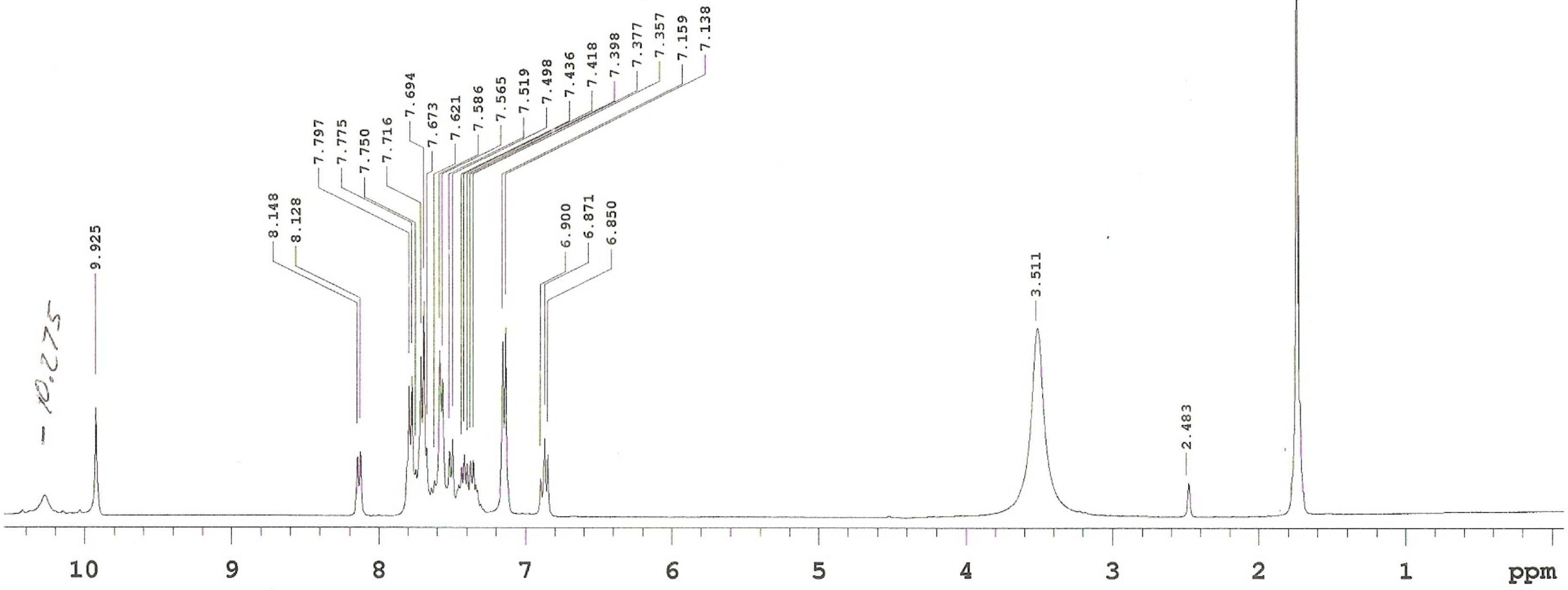

^{13}C-NMR(DMSO-d_6) Varian Mercury 400 plus

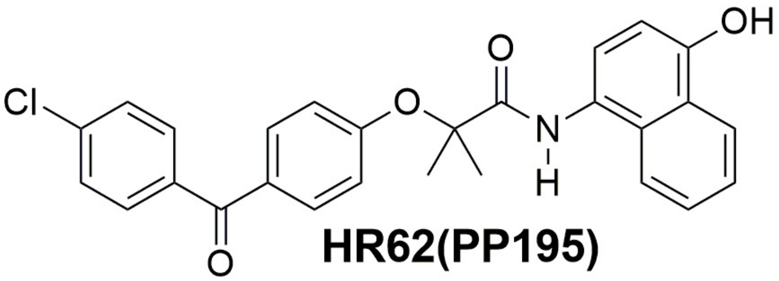

HR62(PP195)

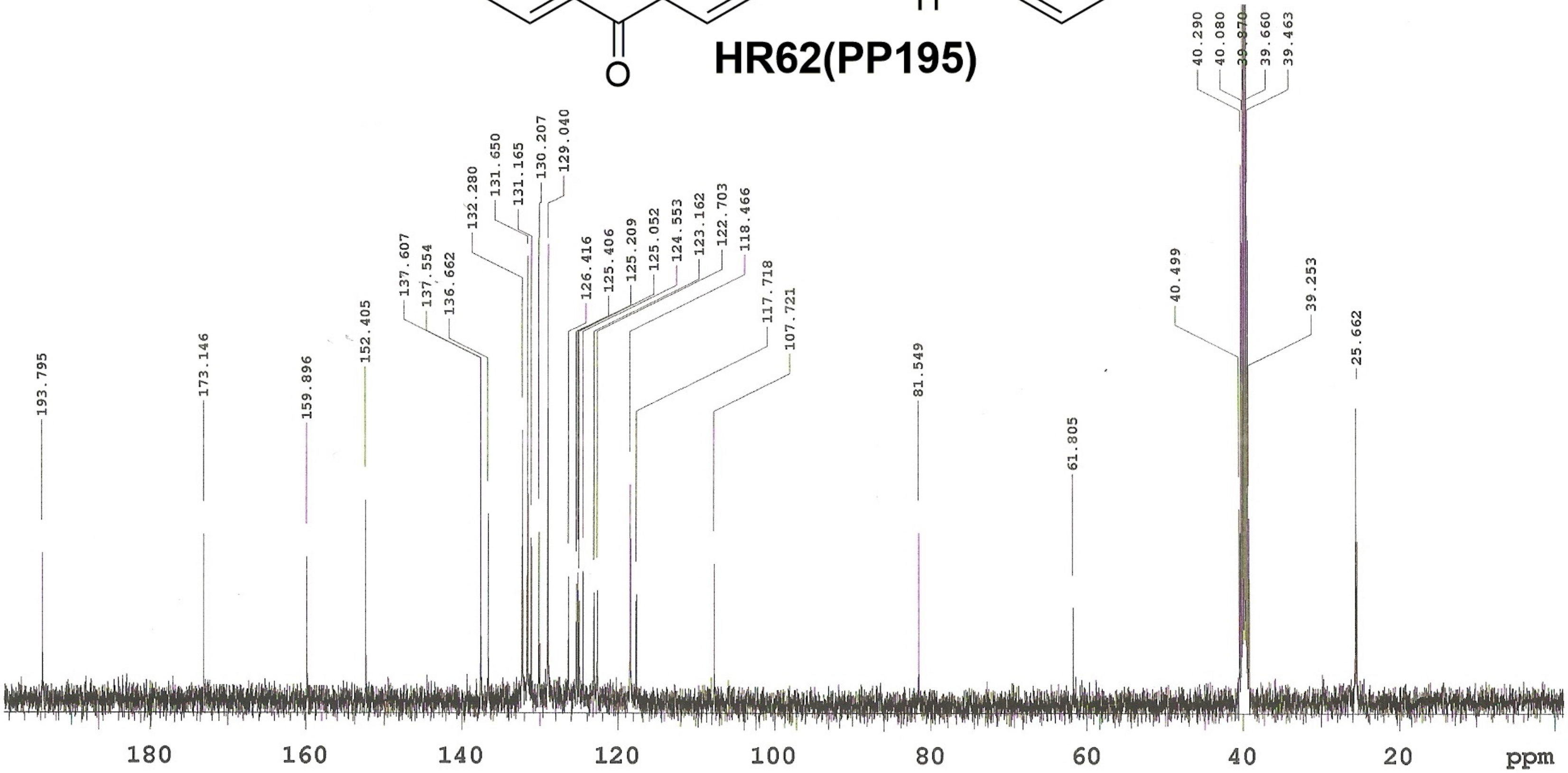

^{1}H-NMR(DMSO-d_6) Varian Mercury 400 Plus

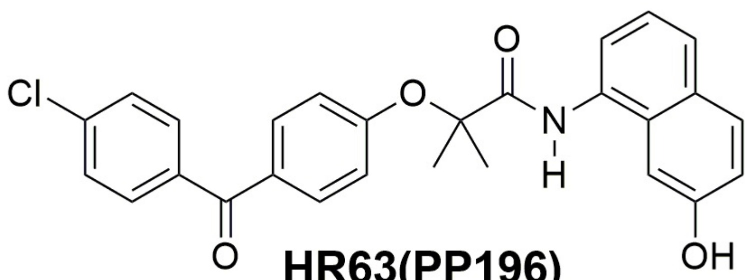

HR63(PP196)

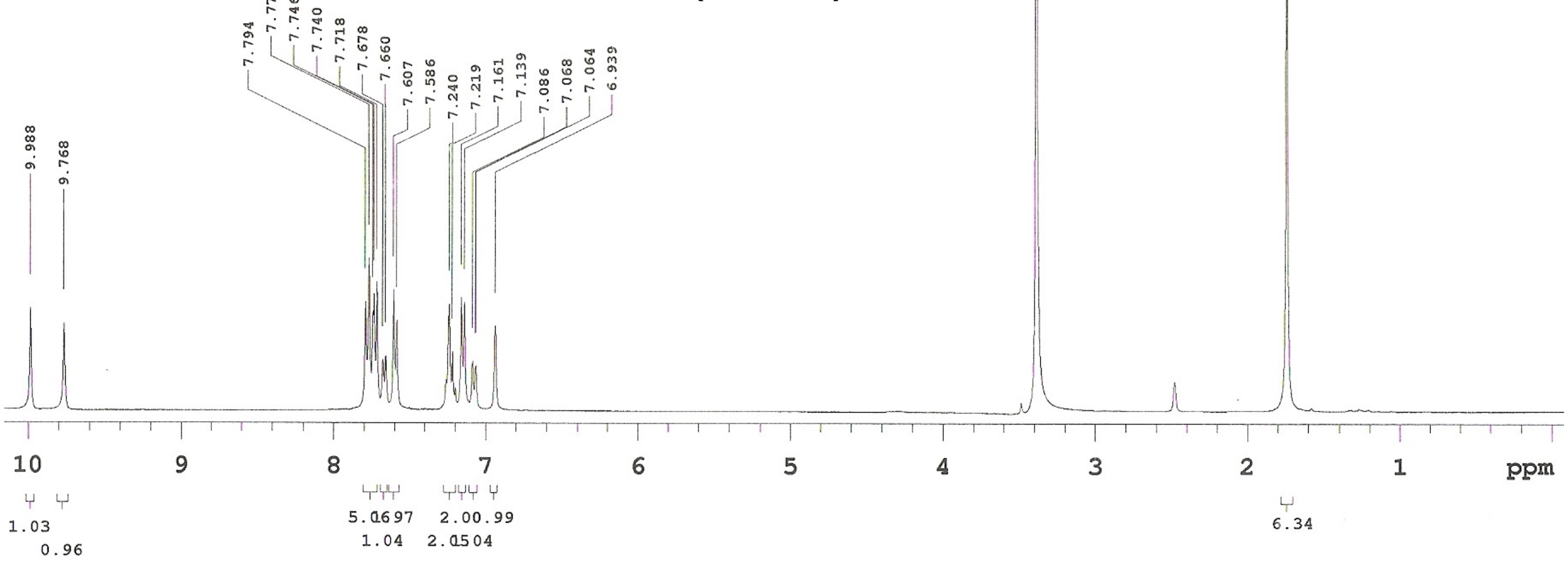

^{1}H-NMR($DMSO-d_6$) Varian Mercury 400 Plus

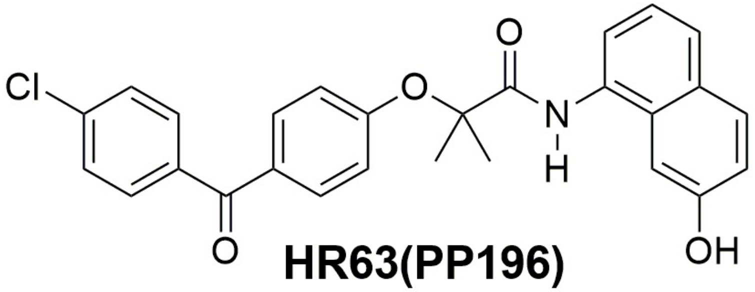

HR63(PP196)

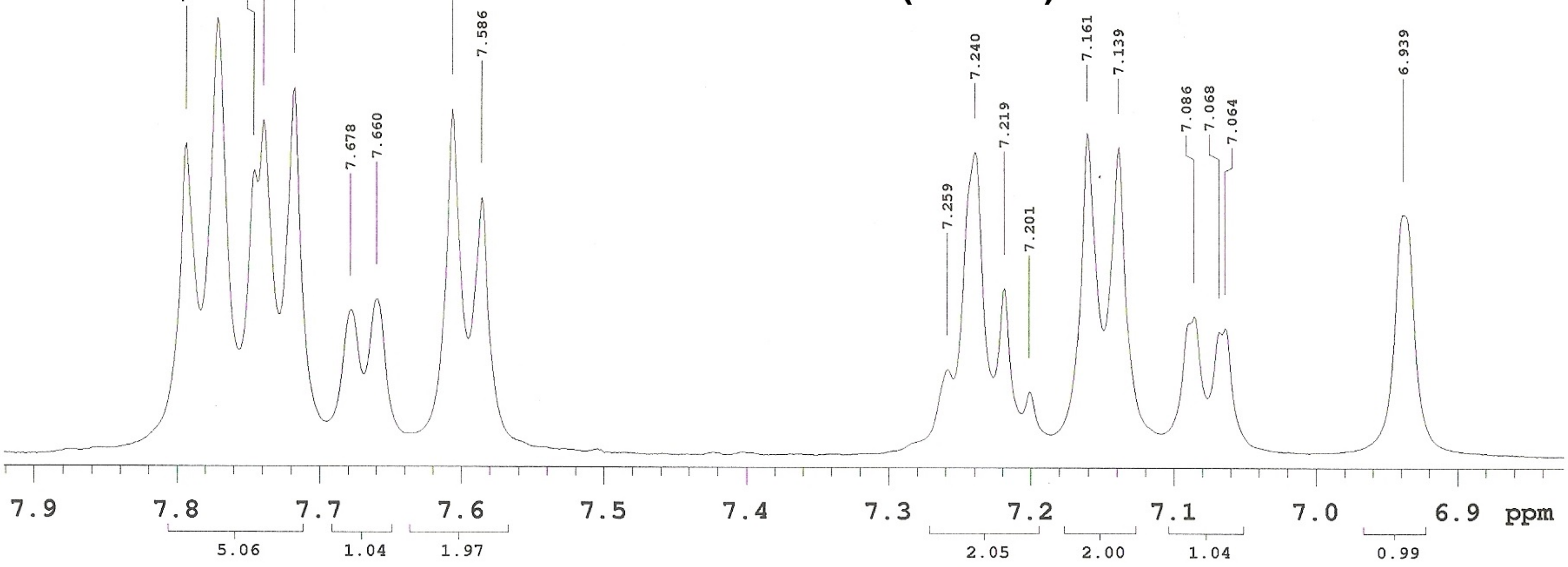

^{13}C-NMR(DMSO-d_6) Varian Mercury 400 plus

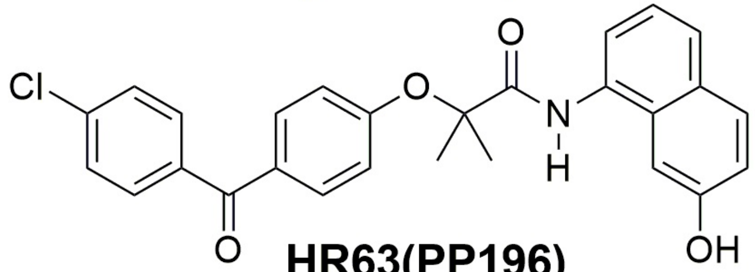

HR63(PP196)

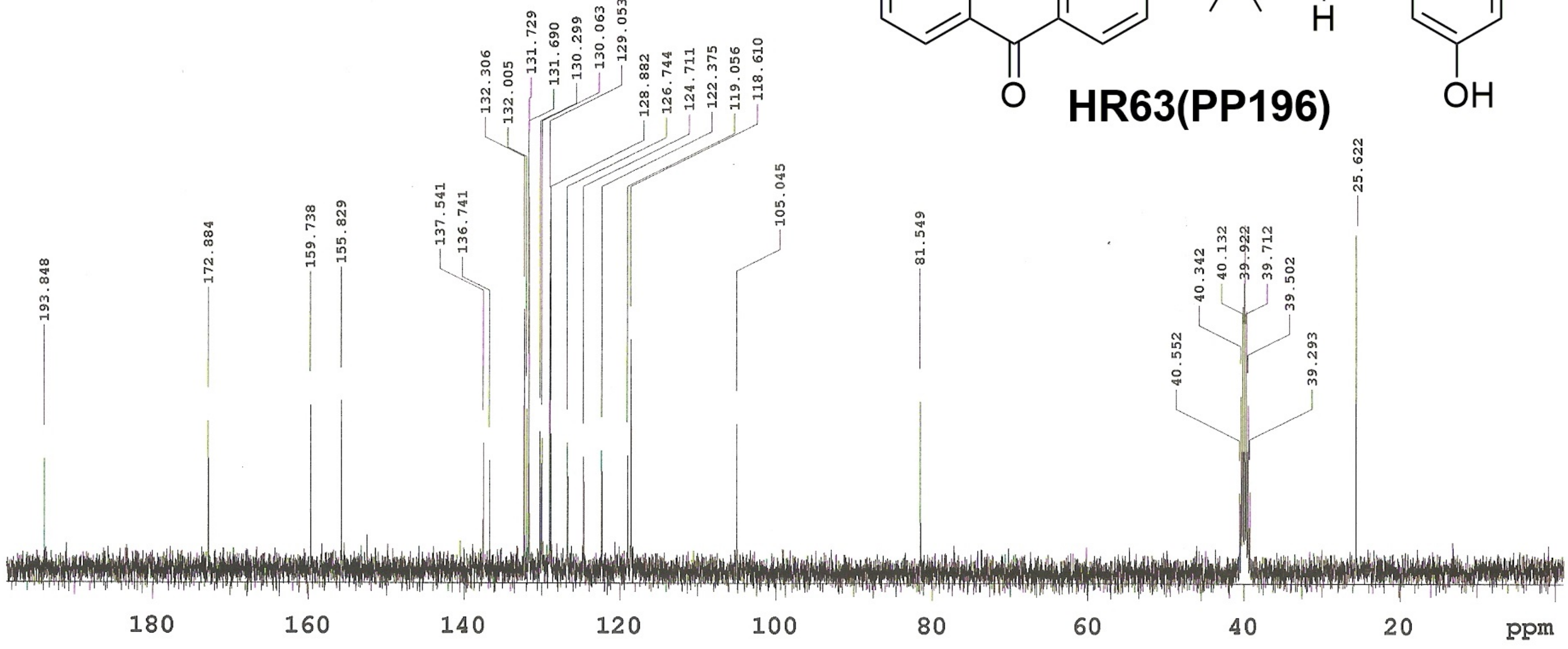

^{1}H-NMR(DMSO-d_6) Varian Mercury 400 Plus

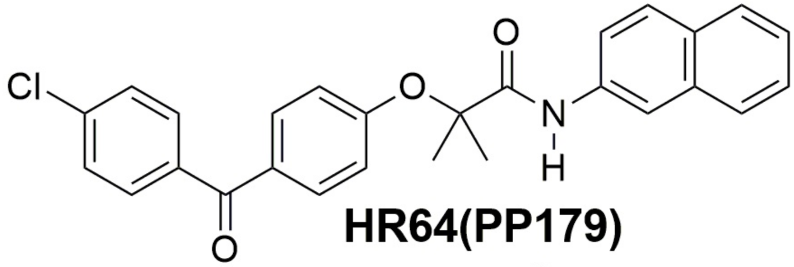

HR64(PP179)

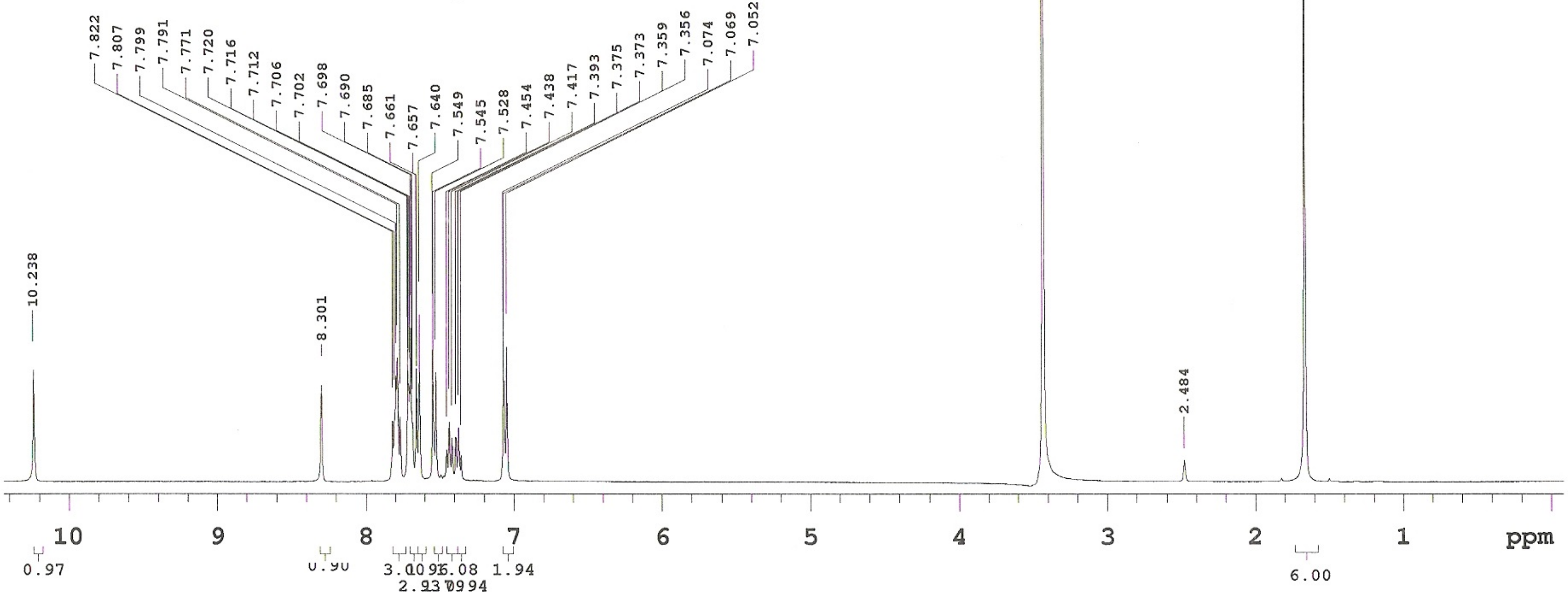

^{1}H-NMR(DMSO-d_6) Varian Mercury 400 Plus

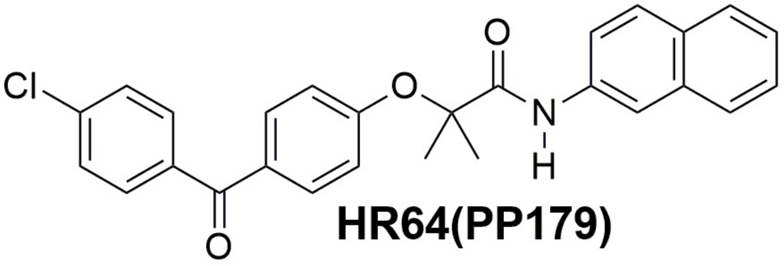

HR64(PP179)

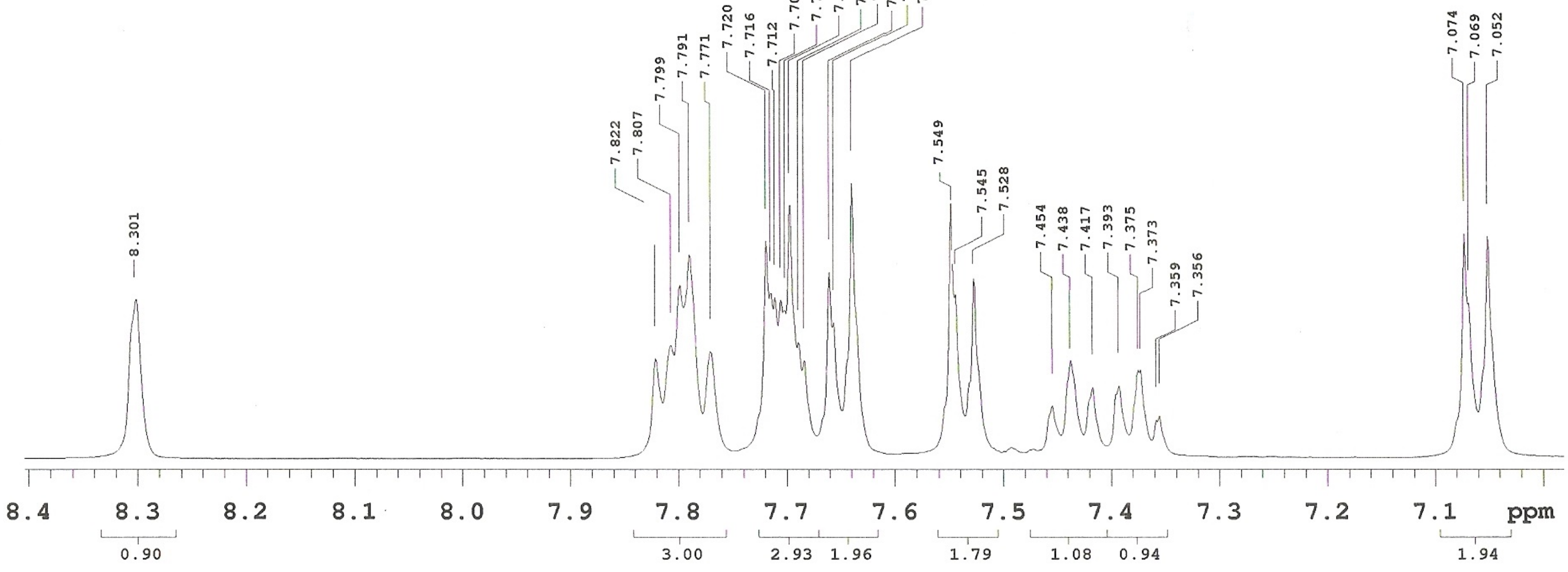

^{13}C-NMR(DMSO-d_6) Varian Mercury 400 plus

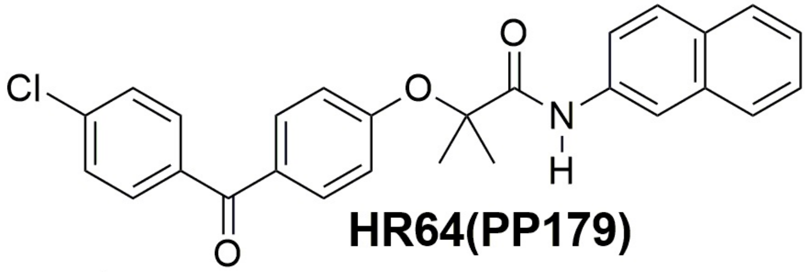

HR64(PP179)

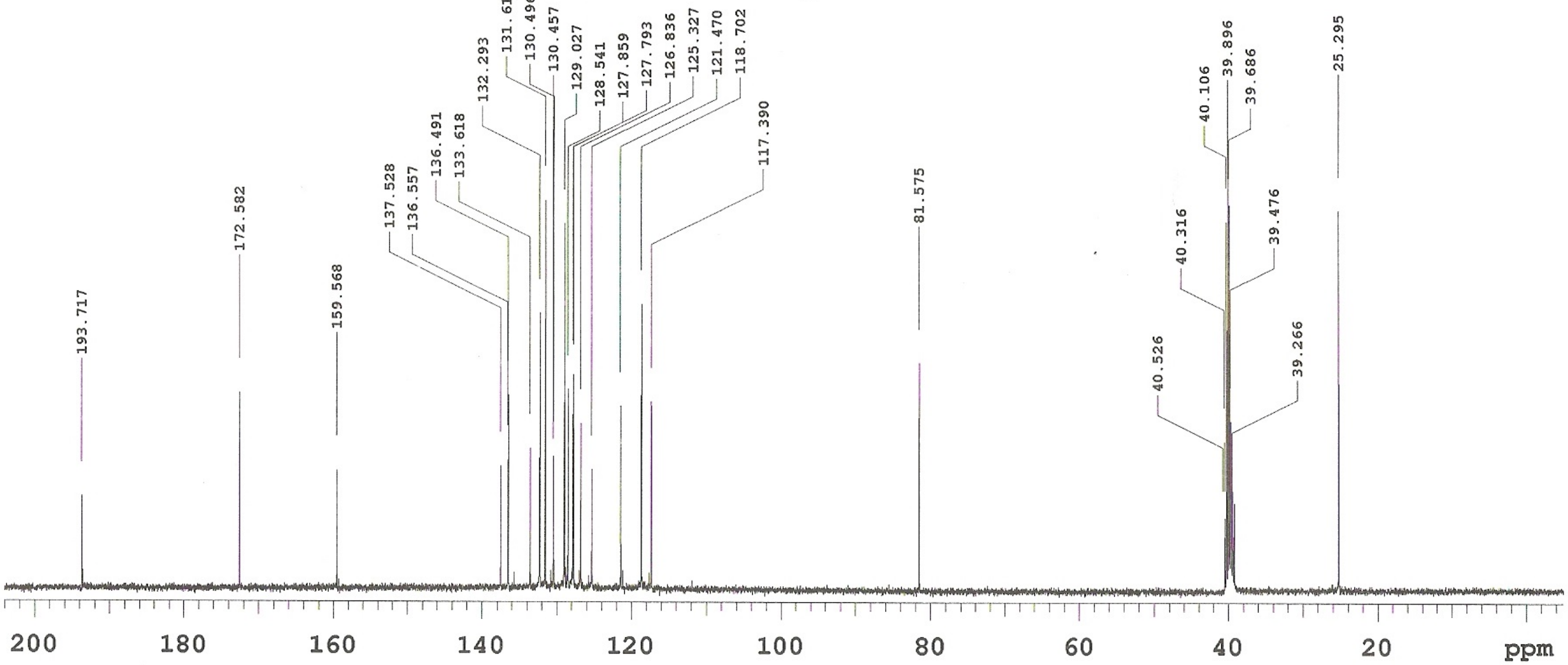

^{1}H-NMR(DMSO-d_6) Varian Mercury 400 Plus

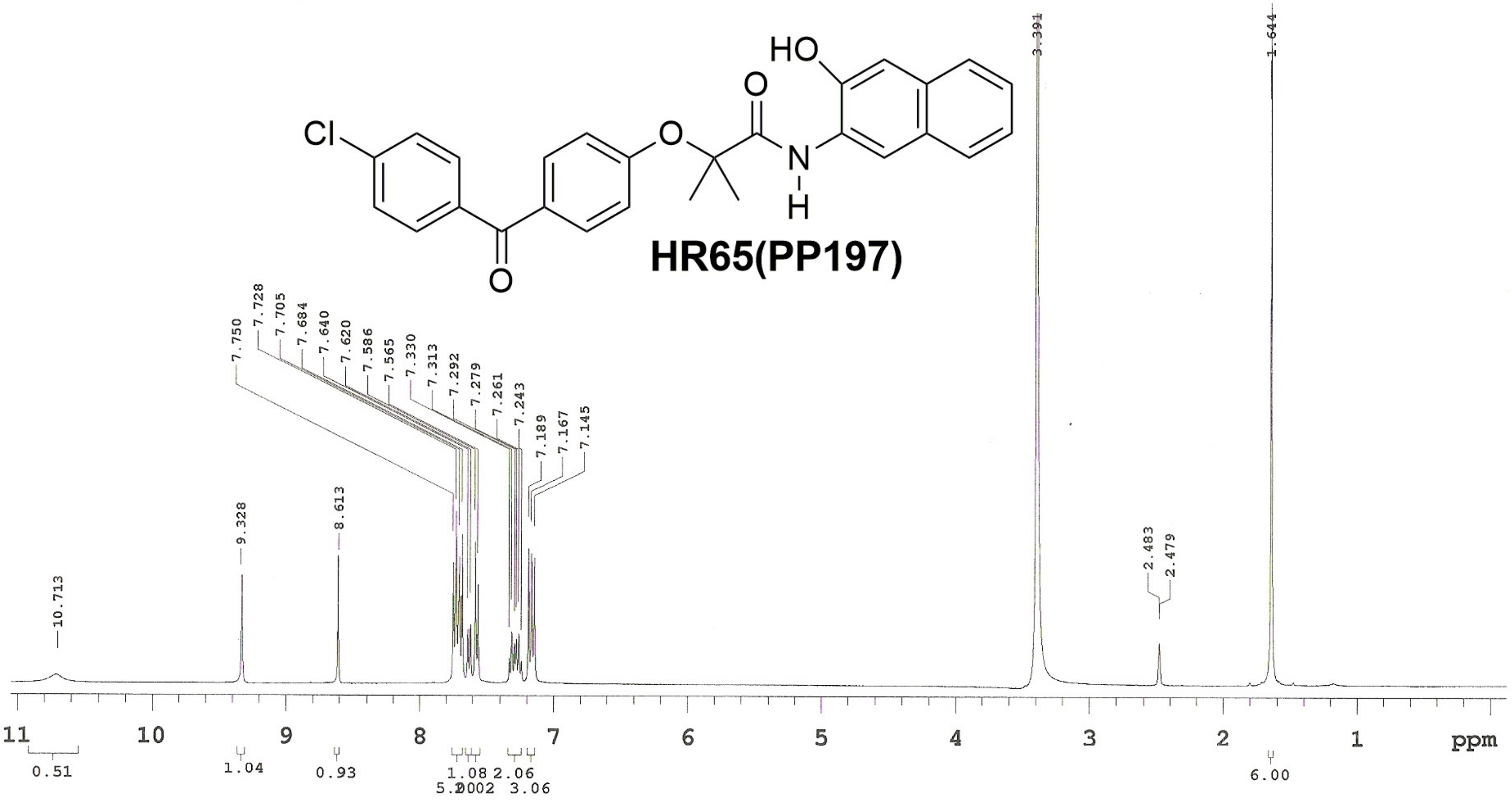

^{1}H-NMR(DMSO-d_6) Varian Mercury 400 Plus

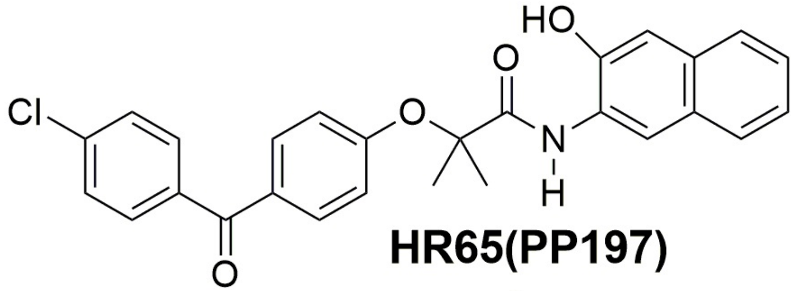

HR65(PP197)

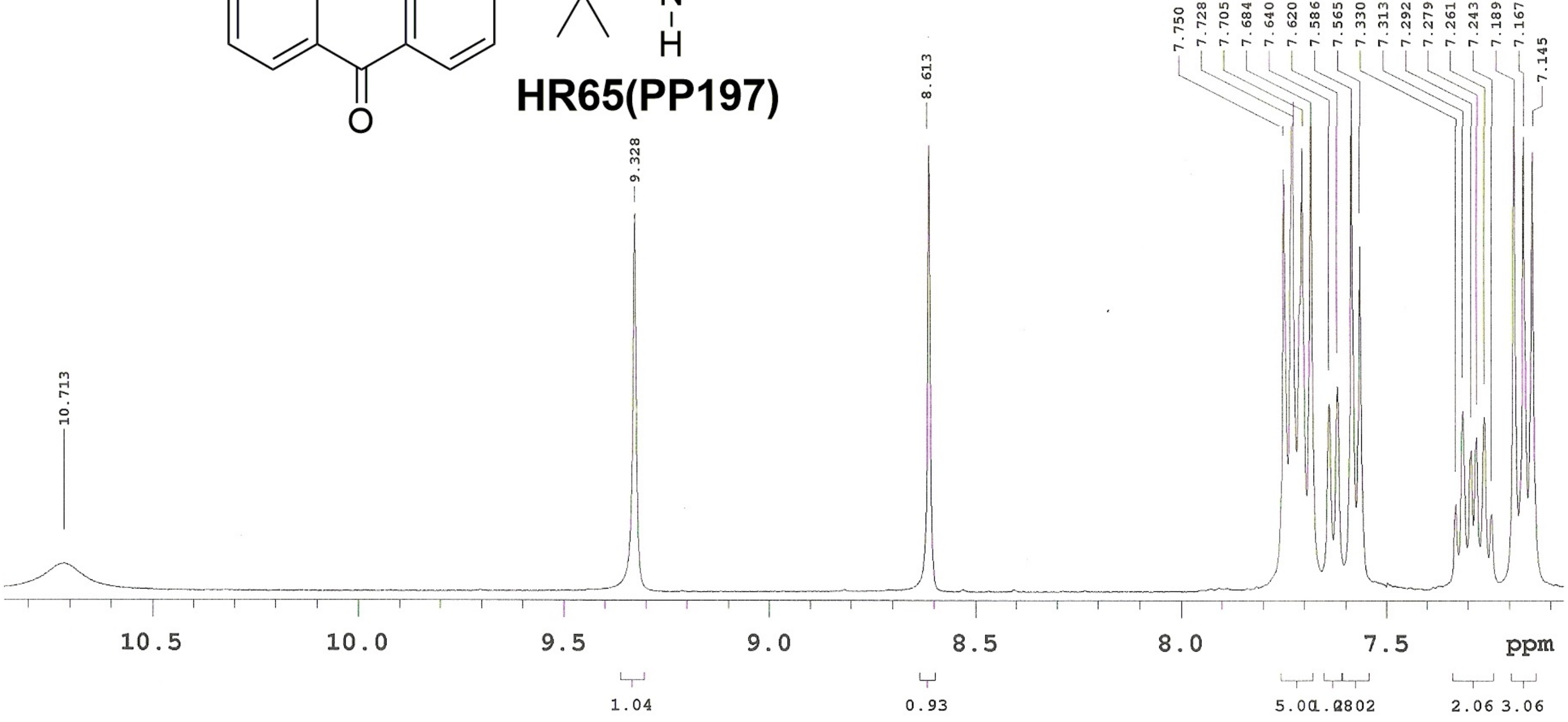

^{13}C-NMR(DMSO-d_6) Varian Mercury 400 plus

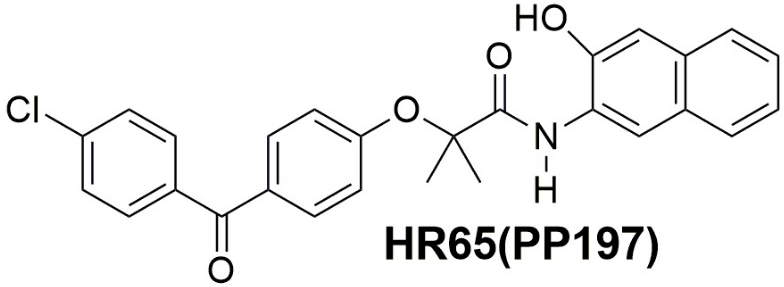

HR65(PP197)

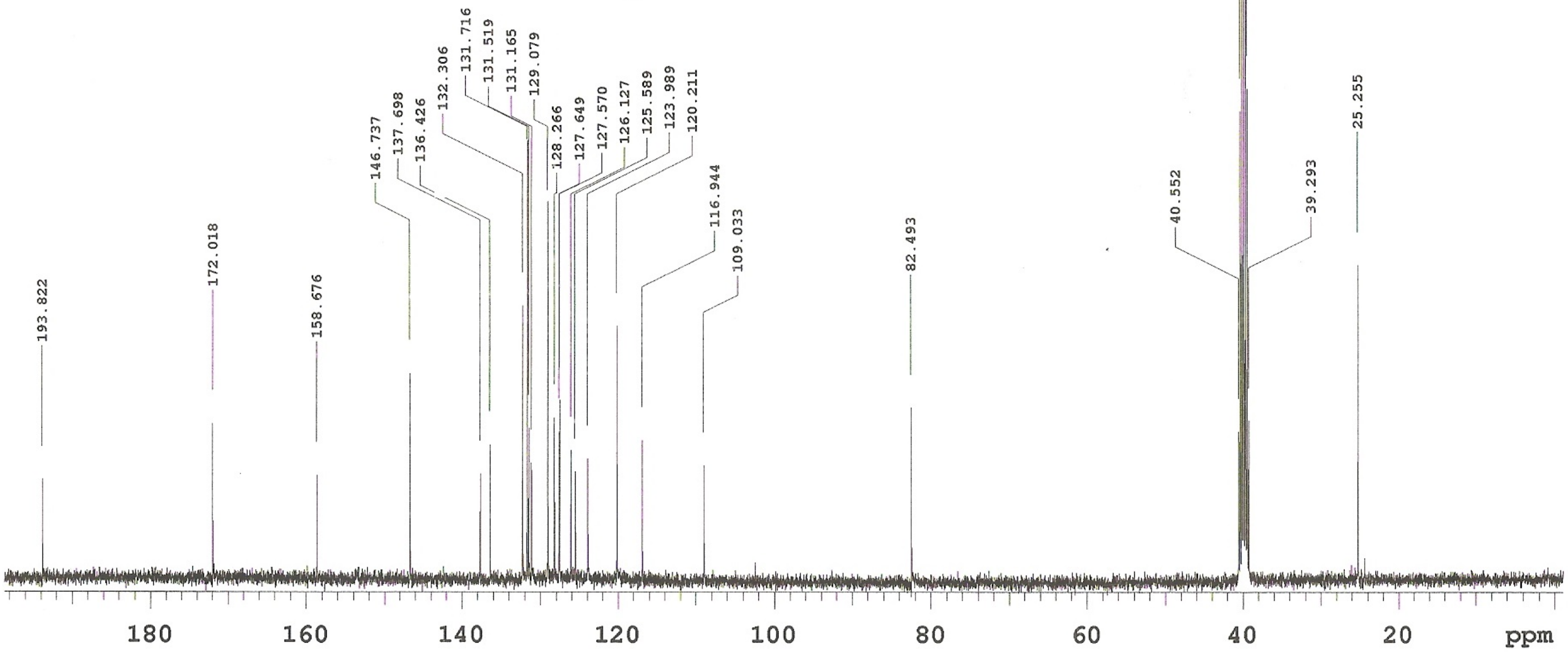

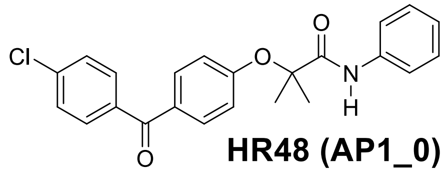

HR48 (AP1_0)

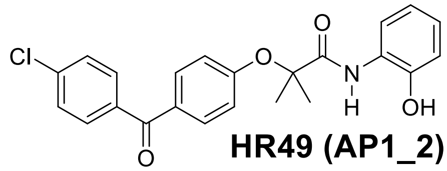

HR49 (AP1_2)

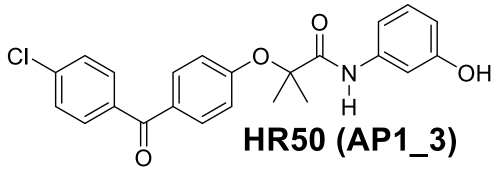

HR50 (AP1_3)

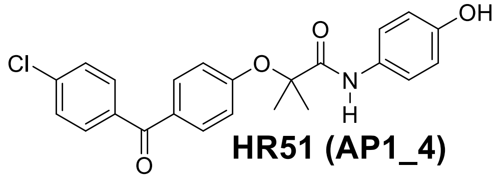

HR51 (AP1_4)

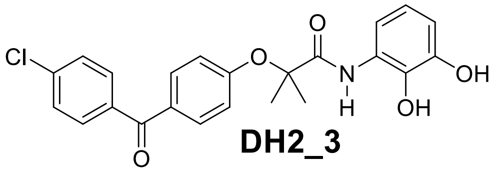

DH2_3

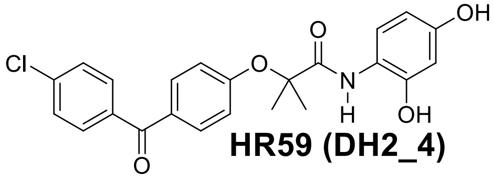

HR59 (DH2_4)

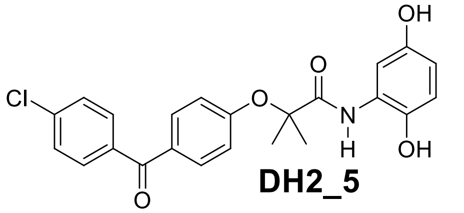

DH2_5

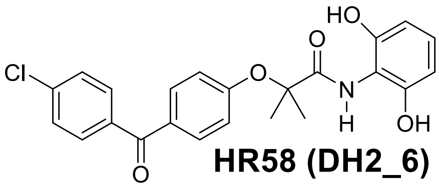

HR58 (DH2_6)

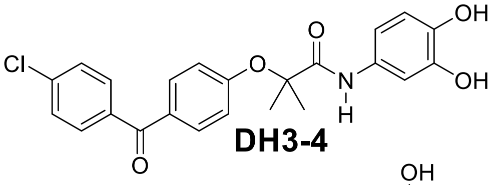

DH3-4

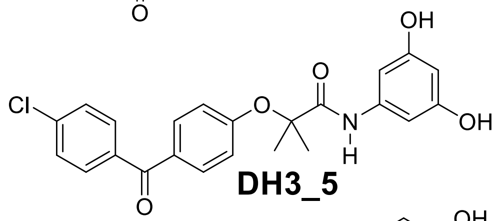

DH3_5

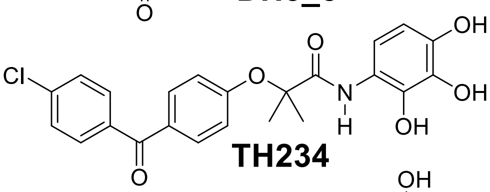

TH234

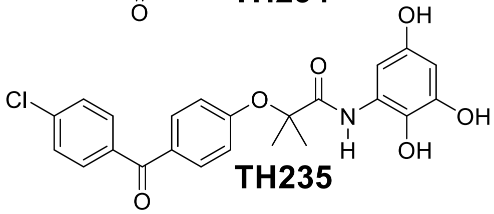

TH235

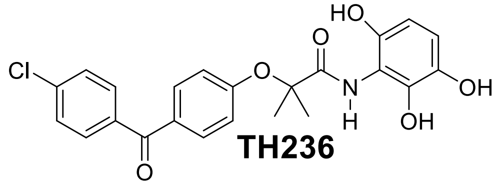

TH236

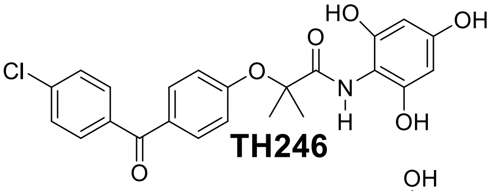

TH246

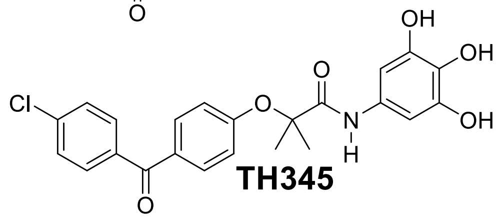

TH345

Scheme 1. Structures of hydroxy only *N*-phenyl **BPA** derivatives

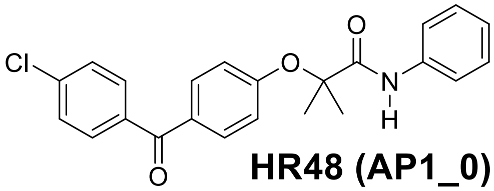

HR48 (AP1_0)

Calc.	Marvin 19.4		HLB	logS	PL	MPA	MSA	RF	HBA	LogBB
		T0	2.88	-7.07	42.67	43.73	551.01	111.47	3	0.20
ClogP	5.79	0								
ClogD	5.79	0								
MW	393.86	0.76								
TPSA	55.40	1								
HBD	1	0.83								
pKa	12.76	0								
CNS MPO	2.59									

HLB = hydrophilic-lipophilic balance; logS = water solubility; PL = polarizability(Å^3); Minimal Projection Area (Å^2); MSA = molecular surface area; RF = Refractivity; HBA = hydrogen bond acceptors; LogBB = blood-brain distribution; ClogP = partitioning; ClogD = distribution at pKa; TPSA = polar surface area; HBD = hydrogen bond donors; CNS MPO = score for CNS penetration.

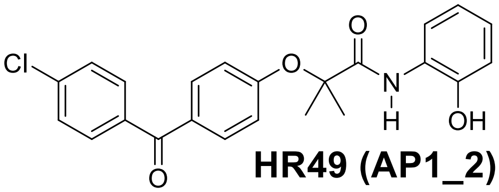

HR49 (AP1_2)

Calc.	Marvin 19.4		HLB	logS	PL	MPA	MSA	RF	HBA	LogBB
		T0	4.53	-6.61	43.28	43.75	561.60	113.45	4	-0.15
ClogP	5.49	0								
ClogD	5.44	0								
MW	409.86	0.64								
TPSA	75.63	1								
HBD	2	0.5								
pKa	8.76	0.62								
CNS MPO	2.76									

HLB = hydrophilic-lipophilic balance; logS = water solubility; PL = polarizability(Å^3); Minimal Projection Area (Å^2); MSA = molecular surface area; RF = Refractivity; HBA = hydrogen bond acceptors; LogBB = blood-brain distribution; ClogP = partitioning; ClogD = distribution at pKa; TPSA = polar surface area; HBD = hydrogen bond donors; CNS MPO = score for CNS penetration.

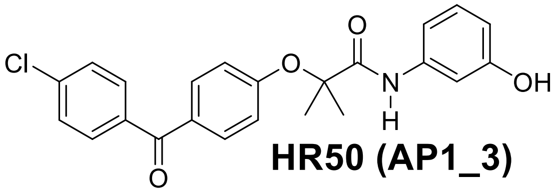

HR50 (AP1_3)

Calc.	Marvin 19.4		HLB	logS	PL	MPA	MSA	RF	HBA	LogBB
		T0	4.53	-6.61	43.28	45.22	561.92	113.45	4	-0.15
ClogP	5.49	0								
ClogD	5.49	0								
MW	409.86	0.64								
TPSA	75.63	1								
HBD	2	0.5								
pKa	9.29	0.36								
CNS MPO	2.5									

HLB = hydrophilic-lipophilic balance; logS = water solubility; PL = polarizability(Å^3); Minimal Projection Area (Å^2); MSA = molecular surface area; RF = Refractivity; HBA = hydrogen bond acceptors; LogBB = blood-brain distribution; ClogP = partitioning; ClogD = distribution at pKa; TPSA = polar surface area; HBD = hydrogen bond donors; CNS MPO = score for CNS penetration.

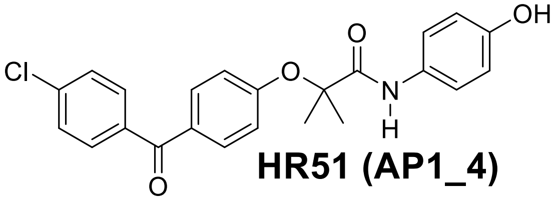

HR51 (AP1_4)

Calc.	Marvin 19.4		HLB	logS	PL	MPA	MSA	RF	HBA	LogBB
		T0	4.53	-6.61	43.28	49.70	561.95	113.45	4	-0.15
ClogP	5.49	0								
ClogD	5.49	0								
MW	409.86	0.64								
TPSA	75.63	1								
HBD	2	0.5								
pKa	9.43	0.29								
CNS MPO	2.45									

HLB = hydrophilic-lipophilic balance; logS = water solubility; PL = polarizability(Å^3); Minimal Projection Area (Å^2); MSA = molecular surface area; RF = Refractivity; HBA = hydrogen bond acceptors; LogBB = blood-brain distribution; ClogP = partitioning; ClogD = distribution at pKa; TPSA = polar surface area; HBD = hydrogen bond donors; CNS MPO = score for CNS penetration.

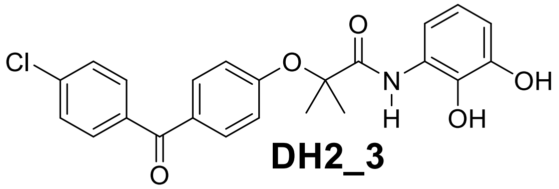

DH2_3

Calc.	Marvin 19.4		HLB	logS	PL	MPA	MSA	RF	HBA	LogBB
			6.16	-6.13	43.91	43.83	572.18	115.43	5	-0.49
ClogP	5.19	0								
ClogD	5.16	0								
MW	435.87	0.46								
TPSA	95.86	0.8								
HBD	3	0.17								
pKa	8.83	0.59								
CNS MPO	2.02									

HLB = hydrophilic-lipophilic balance; logS = water solubility; PL = polarizability(Å^3); Minimal Projection Area (Å^2); MSA = molecular surface area; RF = Refractivity; HBA = hydrogen bond acceptors; LogBB = blood-brain distribution; ClogP = partitioning; ClogD = distribution at pKa; TPSA = polar surface area; HBD = hydrogen bond donors; CNS MPO = score for CNS penetration.

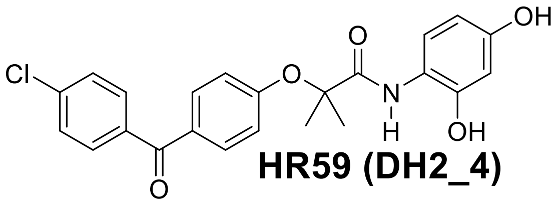

HR59 (DH2_4)

Calc.	Marvin 19.4		HLB	logS	PL	MPA	MSA	RF	HBA	LogBB
		T0	6.16	-6.11	43.90	44.25	572.76	115.43	5	-0.49
ClogP	5.19	0								
ClogD	5.15	0								
MW	425.86	0.53								
TPSA	95.86	0.8								
HBD	3	0.17								
pKa	8.69	0.66								
CNS MPO	2.16									

HLB = hydrophilic-lipophilic balance; logS = water solubility; PL = polarizability(Å^3); Minimal Projection Area (Å^2); MSA = molecular surface area; RF = Refractivity; HBA = hydrogen bond acceptors; LogBB = blood-brain distribution; ClogP = partitioning; ClogD = distribution at pKa; TPSA = polar surface area; HBD = hydrogen bond donors; CNS MPO = score for CNS penetration.

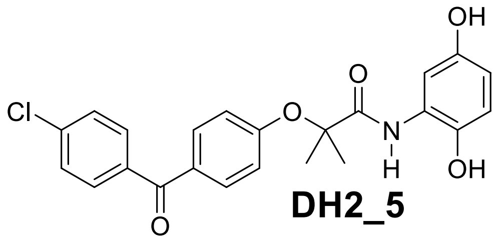

DH2_5

Calc.	Marvin 19.4		HLB	logS	PL	MPA	MSA	RF	HBA	LogBB
		T0	6.16	-6.13	43.90	46.81	570.88	115.43	5	-0.49
ClogP	5.19									
ClogD	5.19									
MW	425.87									
TPSA	95.86									
HBD	3									
pKa	9.10									
CNS MPO	1.88									

HLB = hydrophilic-lipophilic balance; logS = water solubility; PL = polarizability(Å^3); Minimal Projection Area (Å^2); MSA = molecular surface area; RF = Refractivity; HBA = hydrogen bond acceptors; LogBB = blood-brain distribution; ClogP = partitioning; ClogD = distribution at pKa; TPSA = polar surface area; HBD = hydrogen bond donors; CNS MPO = score for CNS penetration.

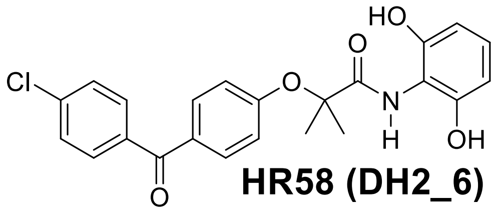

HR58 (DH2_6)

Calc.	Marvin 19.4		HLB	logS	PL	MPA	MSA	RF	HBA	LogBB
		T0	6.61	-6.10	43.91	49.30	570.93	115.43	5	-0.49
ClogP	5.19	0								
ClogD	5.15	0								
MW	425.86	0.53								
TPSA	95.86	0.8								
HBD	3	0.17								
pKa	8.57	0.71								
CNS MPO	2.21									

HLB = hydrophilic-lipophilic balance; logS = water solubility; PL = polarizability(Å^3); Minimal Projection Area (Å^2); MSA = molecular surface area; RF = Refractivity; HBA = hydrogen bond acceptors; LogBB = blood-brain distribution; ClogP = partitioning; ClogD = distribution at pKa; TPSA = polar surface area; HBD = hydrogen bond donors; CNS MPO = score for CNS penetration.

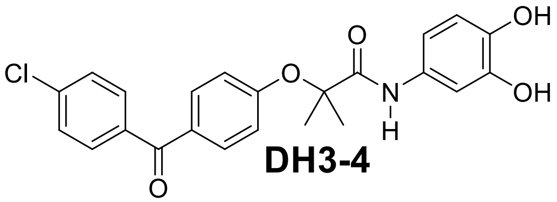

DH3-4

Calc.	Marvin 19.4		HLB	logS	PL	MPA	MSA	RF	HBA	LogBB
		T0	6.16	-6.15	43.90	47.01	571.54	115.43	5	-0.49
ClogP	5.19	0								
ClogD	5.16	0								
MW	425.87	0.53								
TPSA	95.86	0.8								
HBD	3	0.17								
pKa	9.11	0.45								
CNS MPO	1.95									

HLB = hydrophilic-lipophilic balance; logS = water solubility; PL = polarizability(Å^3); Minimal Projection Area (Å^2); MSA = molecular surface area; RF = Refractivity; HBA = hydrogen bond acceptors; LogBB = blood-brain distribution; ClogP = partitioning; ClogD = distribution at pKa; TPSA = polar surface area; HBD = hydrogen bond donors; CNS MPO = score for CNS penetration.

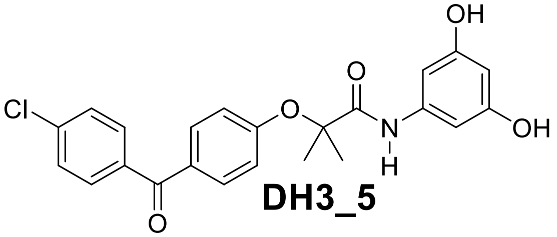

DH3_5

Calc.	Marvin 19.4		HLB	logS	PL	MPA	MSA	RF	HBA	LogBB
		T0	6.16	-6.15	43.90	49.43	572.97	115.43	5	-0.49
ClogP	5.19	0								
ClogD	5.16	0								
MW	425.87	0.53								
TPSA	95.86	0.8								
HBD	3	01.7								
pKa	8.97	0.51								
CNS MPO	2.01									

HLB = hydrophilic-lipophilic balance; logS = water solubility; PL = polarizability(Å^3); Minimal Projection Area (Å^2); MSA = molecular surface area; RF = Refractivity; HBA = hydrogen bond acceptors; LogBB = blood-brain distribution; ClogP = partitioning; ClogD = distribution at pKa; TPSA = polar surface area; HBD = hydrogen bond donors; CNS MPO = score for CNS penetration.

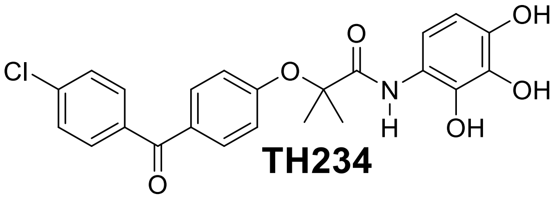

TH234

Calc.	Marvin 19.4		HLB	logS	PL	MPA	MSA	RF	HBA	LogBB
		T0	7.78	-5.65	44.54	44.24	580.70	117.41	6	-0.84
ClogP	4.88	0.06								
ClogD	4.82	0								
MW	441.86	0.42								
TPSA	116.09	0.13								
HBD	4	0								
pKa	8.59	0.71								
CNS MPO	1.32									

HLB = hydrophilic-lipophilic balance; logS = water solubility; PL = polarizability(Å^3); Minimal Projection Area (Å^2); MSA = molecular surface area; RF = Refractivity; HBA = hydrogen bond acceptors; LogBB = blood-brain distribution; ClogP = partitioning; ClogD = distribution at pKa; TPSA = polar surface area; HBD = hydrogen bond donors; CNS MPO = score for CNS penetration.

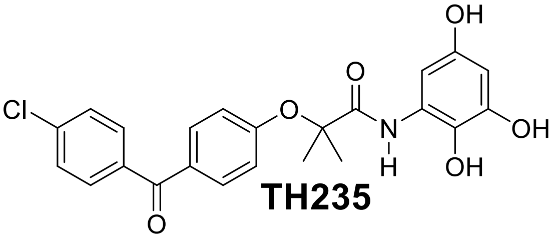

TH235

Calc.	Marvin 19.4		HLB	logS	PL	MPA	MSA	RF	HBA	LogBB
		T0	7.78	-5.69	44.54	47.22	583.06	117.41	6	-0.84
ClogP	4.88	0.06								
ClogD	4.86	0								
MW	441.86	0.42								
TPSA	116.09	0.13								
HBD	4	0								
pKa	9.02	0.49								
CNS MPO	1.10									

HLB = hydrophilic-lipophilic balance; logS = water solubility; PL = polarizability(Å^3); Minimal Projection Area (Å^2); MSA = molecular surface area; RF = Refractivity; HBA = hydrogen bond acceptors; LogBB = blood-brain distribution; ClogP = partitioning; ClogD = distribution at pKa; TPSA = polar surface area; HBD = hydrogen bond donors; CNS MPO = score for CNS penetration.

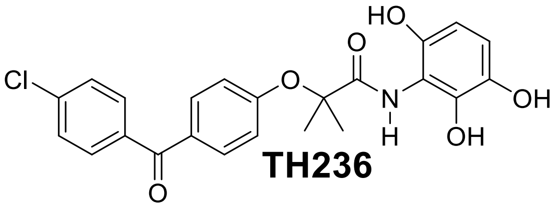

TH236

Calc.	Marvin 19.4		HLB	logS	PL	MPA	MSA	RF	HBA	LogBB
		T0	7.78	-5.66	44.54	50.93	580.70	117.41	6	-0.84
ClogP	4.88	0.06								
ClogD	4.85	0								
MW	441.86	0.42								
TPSA	116.09	0.13								
HBD	4	0								
pKa	8.82	0.49								
CNS MPO	1.10									

HLB = hydrophilic-lipophilic balance; logS = water solubility; PL = polarizability(Å^3); Minimal Projection Area (Å^2); MSA = molecular surface area; RF = Refractivity; HBA = hydrogen bond acceptors; LogBB = blood-brain distribution; ClogP = partitioning; ClogD = distribution at pKa; TPSA = polar surface area; HBD = hydrogen bond donors; CNS MPO = score for CNS penetration.

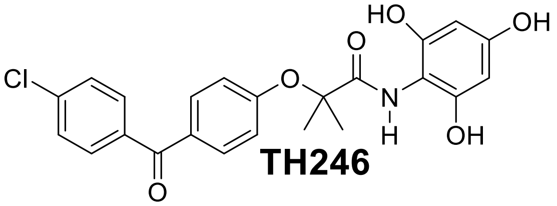

TH246

Calc.	Marvin 19.4		HLB	logS	PL	MPA	MSA	RF	HBA	LogBB
		T0	7.78	-5.64	44.54	48.27	581.00	117.41	6	-0.84
ClogP	4.88	0.06								
ClogD	4.81	0								
MW	441.86	0.42								
TPSA	116.09	0.13								
HBD	4	0								
pKa	8.54	0.73								
CNS MPO	1.34									

HLB = hydrophilic-lipophilic balance; logS = water solubility; PL = polarizability(Å^3); Minimal Projection Area (Å^2); MSA = molecular surface area; RF = Refractivity; HBA = hydrogen bond acceptors; LogBB = blood-brain distribution; ClogP = partitioning; ClogD = distribution at pKa; TPSA = polar surface area; HBD = hydrogen bond donors; CNS MPO = score for CNS penetration.

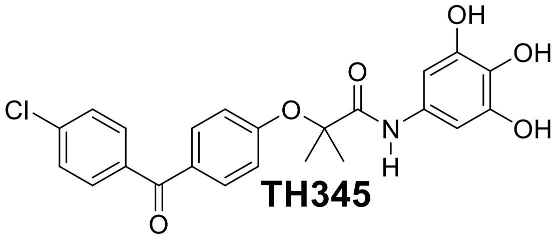

TH345

Calc.	Marvin 19.4		HLB	logS	PL	MPA	MSA	RF	HBA	LogBB
		T0	7.78	-5.68	44.54	48.64	581.66	117.41	6	-0.84
ClogP	4.88	0.06								
ClogD	4.85	0								
MW	441.86	0.42								
TPSA	116.09	0.13								
HBD	4	0								
pKa	8.77	0.62								
CNS MPO	1.23									

HLB = hydrophilic-lipophilic balance; logS = water solubility; PL = polarizability(Å^3); Minimal Projection Area (Å^2); MSA = molecular surface area; RF = Refractivity; HBA = hydrogen bond acceptors; LogBB = blood-brain distribution; ClogP = partitioning; ClogD = distribution at pKa; TPSA = polar surface area; HBD = hydrogen bond donors; CNS MPO = score for CNS penetration.

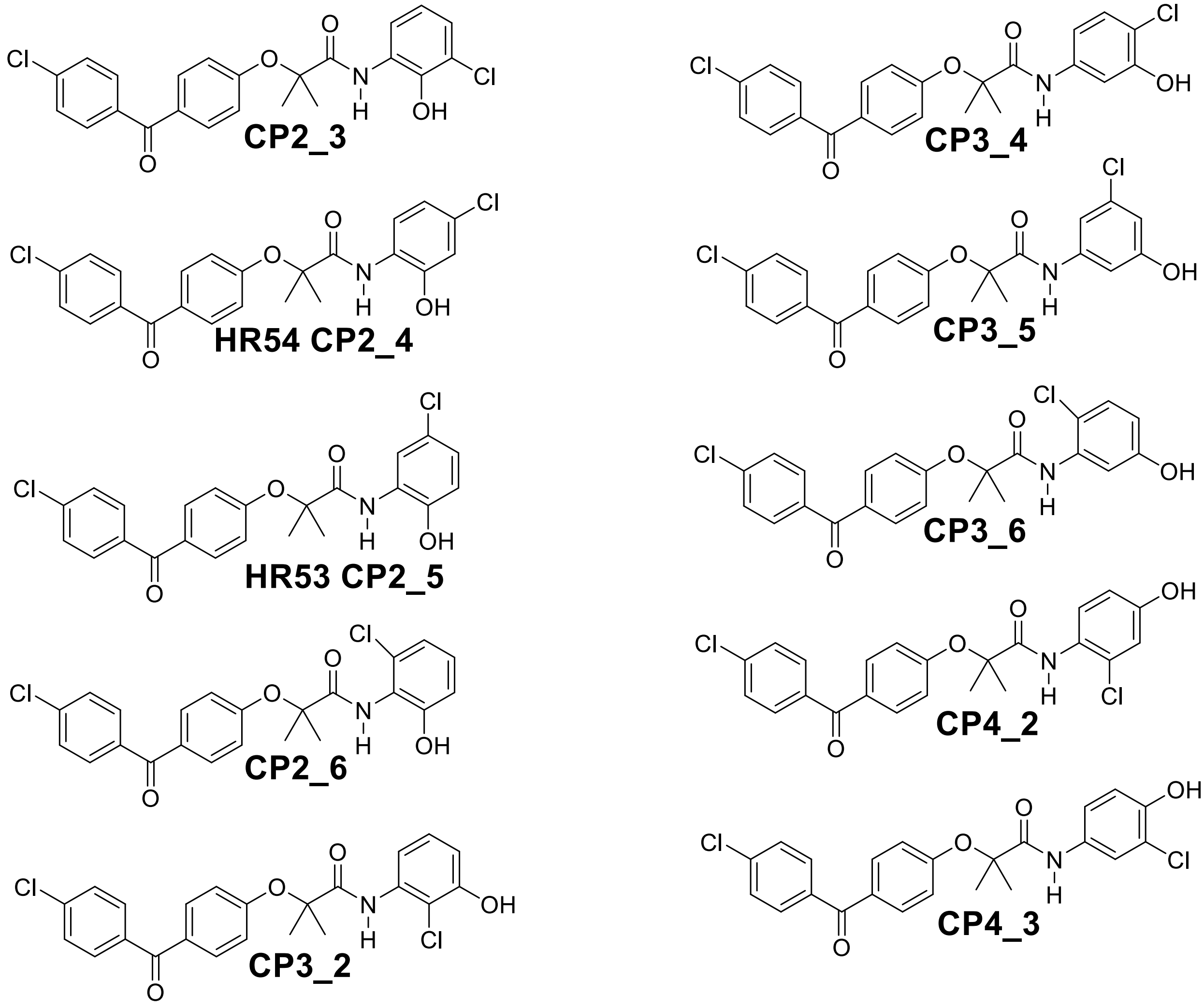

Scheme 2. Chloro hydroxyphenyl **BPA** derivatives (**CP** derivatives)

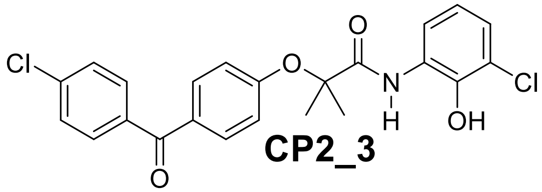

CP2_3

Calc.	Marvin 19.4		HLB	logS	PL	MPA	MSA	RF	HBA	LogBB
		T0	4.80	-6.85	45.19	45.66	575.52	118.26	4	-0.05
ClogP	6.09	0								
ClogD	5.30	0								
MW	444.31	0.4								
TPSA	75.63	1								
HBD	2	0.5								
pKa	7.26	1								
CNS MPO	2.90									

HLB = hydrophilic-lipophilic balance; logS = water solubility; PL = polarizability(Å^3); Minimal Projection Area (Å^2); MSA = molecular surface area; RF = Refractivity; HBA = hydrogen bond acceptors; LogBB = blood-brain distribution; ClogP = partitioning; ClogD = distribution at pKa; TPSA = polar surface area; HBD = hydrogen bond donors; CNS MPO = score for CNS penetration.

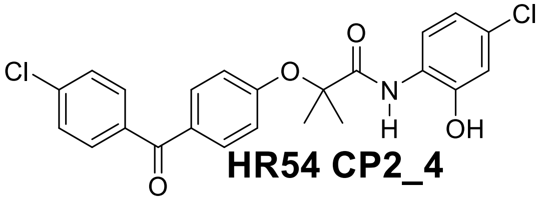

HR54 CP2_4

Calc.	Marvin 19.4		HLB	logS	PL	MPA	MSA	RF	HBA	LogBB
		T0	4.80	-7.08	45.17	46.41	576.77	118.26	4	-0.05
ClogP	6.09	0								
ClogD	5.95	0								
MW	444.31	0.4								
TPSA	75.63	1								
HBD	2	0.5								
pKa	8.01	1								
CNS MPO	2.9									

HLB = hydrophilic-lipophilic balance; logS = water solubility; PL = polarizability(Å^3); Minimal Projection Area (Å^2); MSA = molecular surface area; RF = Refractivity; HBA = hydrogen bond acceptors; LogBB = blood-brain distribution; ClogP = partitioning; ClogD = distribution at pKa; TPSA = polar surface area; HBD = hydrogen bond donors; CNS MPO = score for CNS penetration.

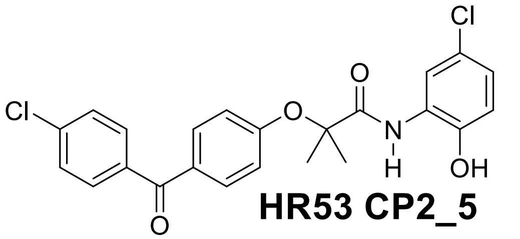

HR53 CP2_5

Calc.	Marvin 19.4		HLB	logS	PL	MPA	MSA	RF	HBA	LogBB
		T0	4.80	-7.15	45.17	49.87	577.82	118.26	4	-0.05
ClogP	6.09	0								
ClogD	5.95	0								
MW	444.31	0.4								
TPSA	75.63	1								
HBD	2	0.5								
pKa	8.22	0.89								
CNS MPO	2.79									

HLB = hydrophilic-lipophilic balance; logS = water solubility; PL = polarizability(Å^3); Minimal Projection Area (Å^2); MSA = molecular surface area; RF = Refractivity; HBA = hydrogen bond acceptors; LogBB = blood-brain distribution; ClogP = partitioning; ClogD = distribution at pKa; TPSA = polar surface area; HBD = hydrogen bond donors; CNS MPO = score for CNS penetration.

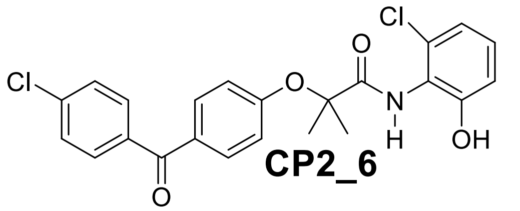

CP2_6

Calc.	Marvin 19.4		HLB	logS	PL	MPA	MSA	RF	HBA	LogBB
		T0	4.80	-6.71	45.19	47.21	575.65	118.26	4	-0.05
ClogP	6.09	0								
ClogD	5.95	0								
MW	444.31	0.4								
TPSA	75.63	1								
HBD	2	0.5								
pKa	8.11	0.95								
CNS MPO	2.85									

HLB = hydrophilic-lipophilic balance; logS = water solubility; PL = polarizability(Å^3); Minimal Projection Area (Å^2); MSA = molecular surface area; RF = Refractivity; HBA = hydrogen bond acceptors; LogBB = blood-brain distribution; ClogP = partitioning; ClogD = distribution at pKa; TPSA = polar surface area; HBD = hydrogen bond donors; CNS MPO = score for CNS penetration.

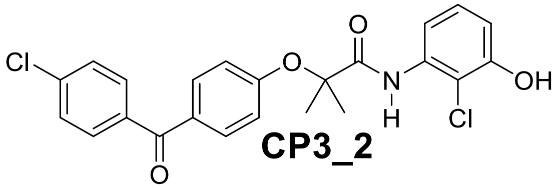

CP3_2

Calc.	Marvin 19.4		HLB	logS	PL	MPA	MSA	RF	HBA	LogBB
		T0	4.80	-7.01	45.20	48.06	574.64	118.26	4	-0.05
ClogP	6.09	0								
ClogD	5.85	0								
MW	444.31	0.4								
TPSA	75.63	1								
HBD	2	0.5								
pKa	7.76	1								
CNS MPO	2.90									

HLB = hydrophilic-lipophilic balance; logS = water solubility; PL = polarizability(Å^3); Minimal Projection Area (Å^2); MSA = molecular surface area; RF = Refractivity; HBA = hydrogen bond acceptors; LogBB = blood-brain distribution; ClogP = partitioning; ClogD = distribution at pKa; TPSA = polar surface area; HBD = hydrogen bond donors; CNS MPO = score for CNS penetration.

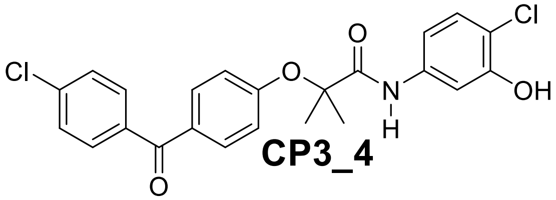

CP3_4

Calc.	Marvin 19.4		HLB	logS	PL	MPA	MSA	RF	HBA	LogBB
		T0	4.80	-6.98	45.19	44.91	577.63	118.26	4	-0.05
ClogP	6.09	0								
ClogD	5.78	0								
MW	444.31	0.4								
TPSA	75.63	1								
HBD	2	0.5								
pKa	7.65	1								
CNS MPO	2.90									

HLB = hydrophilic-lipophilic balance; logS = water solubility; PL = polarizability(Å^3); Minimal Projection Area (Å^2); MSA = molecular surface area; RF = Refractivity; HBA = hydrogen bond acceptors; LogBB = blood-brain distribution; ClogP = partitioning; ClogD = distribution at pKa; TPSA = polar surface area; HBD = hydrogen bond donors; CNS MPO = score for CNS penetration.

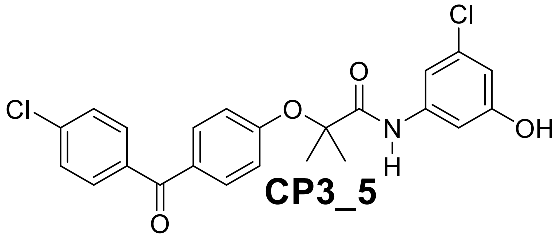

CP3_5

Calc.	Marvin 19.4		HLB	logS	PL	MPA	MSA	RF	HBA	LogBB
		T0	4.80	-7.21	45.17	50.31	578.04	118.26	4	-0.05
ClogP	6.09	0								
ClogD	6.01	0								
MW	444.31	0.4								
TPSA	75.63	1								
HBD	2	0.5								
pKa	8.50	0.75								
CNS MPO	2.65									

HLB = hydrophilic-lipophilic balance; logS = water solubility; PL = polarizability($Å^3$); Minimal Projection Area ($Å^2$); MSA = molecular surface area; RF = Refractivity; HBA = hydrogen bond acceptors; LogBB = blood-brain distribution; ClogP = partitioning; ClogD = distribution at pKa; TPSA = polar surface area; HBD = hydrogen bond donors; CNS MPO = score for CNS penetration.

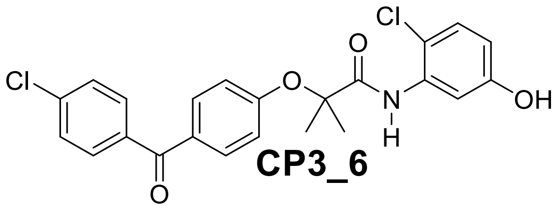

CP3_6

Calc.	Marvin 19.4		HLB	logS	PL	MPA	MSA	RF	HBA	LogBB
		T0	4.80	-7.21	45.18	50.35	576.47	118.26	4	-0.05
ClogP	6.09	0								
ClogD	6.05	0								
MW	444.31	0.4								
TPSA	75.63	1								
HBD	2	0.5								
pKa	8.75	0.63								
CNS MPO	2.53									

HLB = hydrophilic-lipophilic balance; logS = water solubility; PL = polarizability($Å^3$); Minimal Projection Area ($Å^2$); MSA = molecular surface area; RF = Refractivity; HBA = hydrogen bond acceptors; LogBB = blood-brain distribution; ClogP = partitioning; ClogD = distribution at pKa; TPSA = polar surface area; HBD = hydrogen bond donors; CNS MPO = score for CNS penetration.

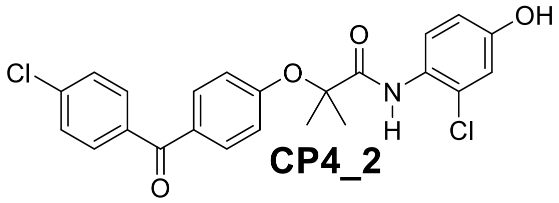

CP4_2

Calc.	Marvin 19.4		HLB	logS	PL	MPA	MSA	RF	HBA	LogBB
		T0	4.80	-7.23	45.19	47.29	576.66	118.26	4	-0.05
ClogP	6.09	0								
ClogD	6.06	0								
MW	444.31	0.4								
TPSA	75.63	1								
HBD	2	0.5								
pKa	8.80	0.6								
CNS MPO	2.50									

HLB = hydrophilic-lipophilic balance; logS = water solubility; PL = polarizability($Å^3$); Minimal Projection Area ($Å^2$); MSA = molecular surface area; RF = Refractivity; HBA = hydrogen bond acceptors; LogBB = blood-brain distribution; ClogP = partitioning; ClogD = distribution at pKa; TPSA = polar surface area; HBD = hydrogen bond donors; CNS MPO = score for CNS penetration.

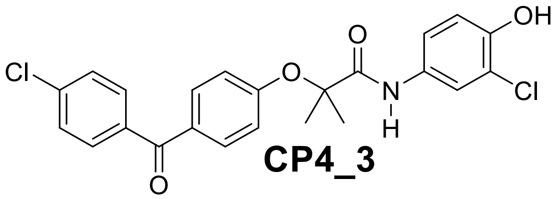

CP4_3

Calc.	Marvin 19.4		HLB	logS	PL	MPA	MSA	RF	HBA	LogBB
		T0	4.80	-7.06	45.13	46.04	576.24	118.26	4	-0.05
ClogP	6.09									
ClogD	5.89									
MW	444.31									
TPSA	75.63									
HBD	2									
pKa	7.90									
CNS MPO	2.90									

HLB = hydrophilic-lipophilic balance; logS = water solubility; PL = polarizability($Å^3$); Minimal Projection Area ($Å^2$); MSA = molecular surface area; RF = Refractivity; HBA = hydrogen bond acceptors; LogBB = blood-brain distribution; ClogP = partitioning; ClogD = distribution at pKa; TPSA = polar surface area; HBD = hydrogen bond donors; CNS MPO = score for CNS penetration.

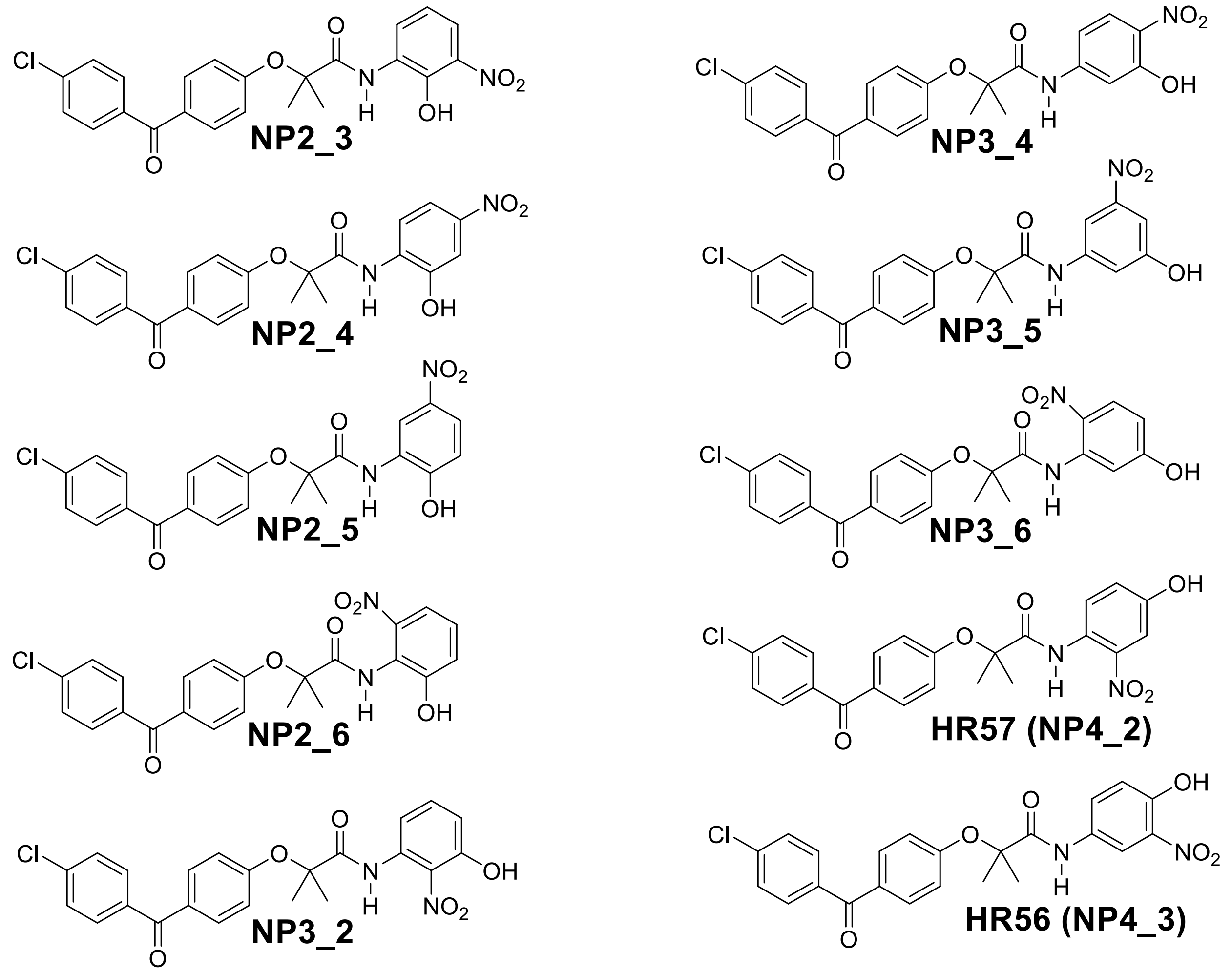

Scheme 3. Nitro hydroxyphenyl **BPA** derivatives (**NP** derivatives)

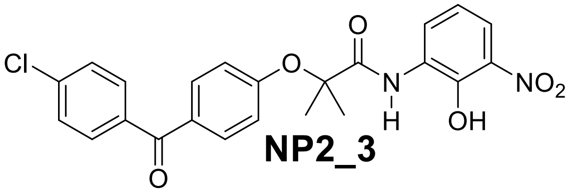

NP2_3

Calc.	Marvin 19.4		HLB	logS	PL	MPA	MSA	RF	HBA	LogBB
		T0	5.18	-5.86	45.20	47.20	596.25	119.77	6	-0.79
ClogP	5.43	0								
ClogD	4.22	0								
MW	454.86	0.32								
TPSA	118.77	0.04								
HBD	2									
pKa	6.18									
CNS MPO	1.86									

HLB = hydrophilic-lipophilic balance; logS = water solubility; PL = polarizability(Å^3); Minimal Projection Area (Å^2); MSA = molecular surface area; RF = Refractivity; HBA = hydrogen bond acceptors; LogBB = blood-brain distribution; ClogP = partitioning; ClogD = distribution at pKa; TPSA = polar surface area; HBD = hydrogen bond donors; CNS MPO = score for CNS penetration.

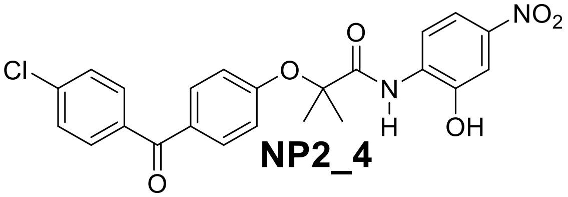

NP2_4

Calc.	Marvin 19.4		HLB	logS	PL	MPA	MSA	RF	HBA	LogBB
		T0	5.18	-6.61	45.20	44.52	597.86	119.77	6	-0.79
ClogP	5.43	0								
ClogD	4.76	0								
MW	454.86	0.32								
TPSA	118.77	0.04								
HBD	2	0.5								
pKa	7.19	1								
CNS MPO	1.86									

HLB = hydrophilic-lipophilic balance; logS = water solubility; PL = polarizability(Å^3); Minimal Projection Area (Å^2); MSA = molecular surface area; RF = Refractivity; HBA = hydrogen bond acceptors; LogBB = blood-brain distribution; ClogP = partitioning; ClogD = distribution at pKa; TPSA = polar surface area; HBD = hydrogen bond donors; CNS MPO = score for CNS penetration.

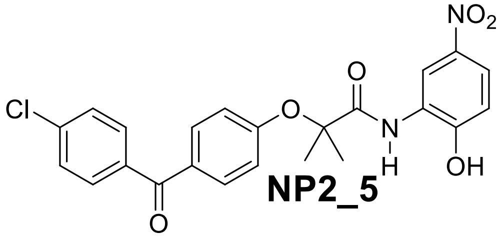

NP2_5

Calc.	Marvin 19.4		HLB	logS	PL	MPA	MSA	RF	HBA	LogBB
		T0	5.18	-6.01	45.20	49.60	597.95	119.77	6	-0.79
ClogP	5.43	0								
ClogD	4.31	0								
MW	454.86	0.32								
TPSA	118.77	0.04								
HBD	2	0.5								
pKa	6.38	1								
CNS MPO	1.86									

HLB = hydrophilic-lipophilic balance; logS = water solubility; PL = polarizability(Å^3); Minimal Projection Area (Å^2); MSA = molecular surface area; RF = Refractivity; HBA = hydrogen bond acceptors; LogBB = blood-brain distribution; ClogP = partitioning; ClogD = distribution at pKa; TPSA = polar surface area; HBD = hydrogen bond donors; CNS MPO = score for CNS penetration.

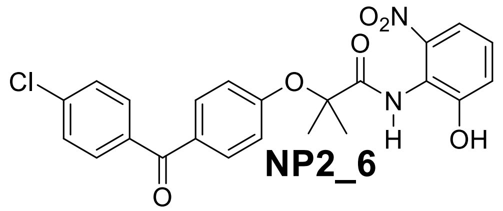

NP2_6

Calc.	Marvin 19.4		HLB	logS	PL	MPA	MSA	RF	HBA	LogBB
		T0	5.18	-6.75	45.20	48.43	596.62	119.77	6	-0.79
ClogP	5.43	0								
ClogD	5.01	0								
MW	454.86	0.32								
TPSA	118.77	0.04								
HBD	2	0.5								
pKa	7.34	1								
CNS MPO	1.86									

HLB = hydrophilic-lipophilic balance; logS = water solubility; PL = polarizability(Å^3); Minimal Projection Area (Å^2); MSA = molecular surface area; RF = Refractivity; HBA = hydrogen bond acceptors; LogBB = blood-brain distribution; ClogP = partitioning; ClogD = distribution at pKa; TPSA = polar surface area; HBD = hydrogen bond donors; CNS MPO = score for CNS penetration.

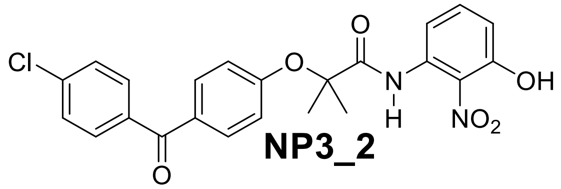

NP3_2

Calc.	Marvin 19.4		HLB	logS	PL	MPA	MSA	RF	HBA	LogBB
		T0	5.18	-6.14	45.21	53.29	597.56	119.77	6	-0.79
ClogP	5.43	0								
ClogD	4.49	0								
MW	454.86	0.32								
TPSA	118.77	0.04								
HBD	2	0.5								
pKa	6.59	1								
CNS MPO	1.86									

HLB = hydrophilic-lipophilic balance; logS = water solubility; PL = polarizability($Å^3$); Minimal Projection Area ($Å^2$); MSA = molecular surface area; RF = Refractivity; HBA = hydrogen bond acceptors; LogBB = blood-brain distribution; ClogP = partitioning; ClogD = distribution at pKa; TPSA = polar surface area; HBD = hydrogen bond donors; CNS MPO = score for CNS penetration.

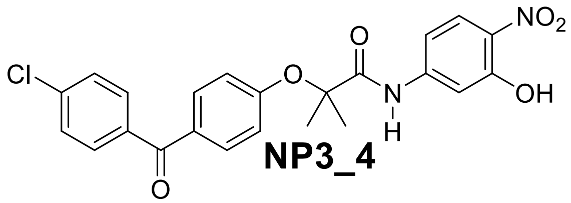

NP3_4

Calc.	Marvin 19.4		HLB	logS	PL	MPA	MSA	RF	HBA	LogBB
		T0	5.18	-6.15	45.20	45.24	599.21	119.77	6	-0.79
ClogP	5.43	0								
ClogD	4.45	0								
MW	454.86	0.32								
TPSA	118.77	0.04								
HBD	2	0.5								
pKa	6.47	1								
CNS MPO	1.86									

HLB = hydrophilic-lipophilic balance; logS = water solubility; PL = polarizability($Å^3$); Minimal Projection Area ($Å^2$); MSA = molecular surface area; RF = Refractivity; HBA = hydrogen bond acceptors; LogBB = blood-brain distribution; ClogP = partitioning; ClogD = distribution at pKa; TPSA = polar surface area; HBD = hydrogen bond donors; CNS MPO = score for CNS penetration.

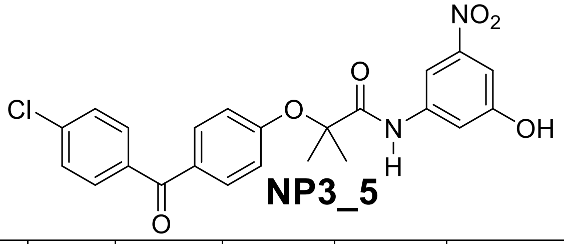

NP3_5

Calc.	Marvin 19.4		HLB	logS	PL	MPA	MSA	RF	HBA	LogBB
		T0	5.18	-6.91	45.20	52.76	599.89	119.77	6	-0.79
ClogP	5.43	0								
ClogD	5.11	0								
MW	454.86	0.32								
TPSA	118.77	0.04								
HBD	2	0.5								
pKa	7.60	1								
CNS MPO	1.86									

HLB = hydrophilic-lipophilic balance; logS = water solubility; PL = polarizability(Å^3); Minimal Projection Area (Å^2); MSA = molecular surface area; RF = Refractivity; HBA = hydrogen bond acceptors; LogBB = blood-brain distribution; ClogP = partitioning; ClogD = distribution at pKa; TPSA = polar surface area; HBD = hydrogen bond donors; CNS MPO = score for CNS penetration.

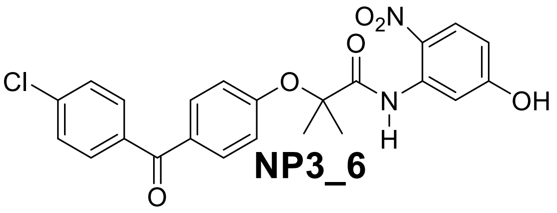

NP3_6

Calc.	Marvin 19.4		HLB	logS	PL	MPA	MSA	RF	HBA	LogBB
		T0	5.18	-6.39	45.20	50.56	598.68	119.77	6	-0.79
ClogP	5.43	0								
ClogD	4.68	0								
MW	454.86	0.32								
TPSA	118.77	0.04								
HBD	2	0.5								
pKa	6.86	1								
CNS MPO	1.86									

HLB = hydrophilic-lipophilic balance; logS = water solubility; PL = polarizability(Å^3); Minimal Projection Area (Å^2); MSA = molecular surface area; RF = Refractivity; HBA = hydrogen bond acceptors; LogBB = blood-brain distribution; ClogP = partitioning; ClogD = distribution at pKa; TPSA = polar surface area; HBD = hydrogen bond donors; CNS MPO = score for CNS penetration.

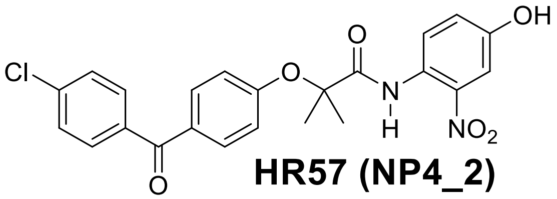

HR57 (NP4_2)

Calc.	Marvin 19.4		HLB	logS	PL	MPA	MSA	RF	HBA	LogBB
		T0	5.18	-7.06	45.20	47.50	598.83	119.77	6	-0.79
ClogP	5.43	0								
ClogD	5.27	0								
MW	454.86	0.32								
TPSA	118.77	0.04								
HBD	2	0.5								
pKa	8.0	1								
CNS MPO	1.86									

HLB = hydrophilic-lipophilic balance; logS = water solubility; PL = polarizability(Å^3); Minimal Projection Area (Å^2); MSA = molecular surface area; RF = Refractivity; HBA = hydrogen bond acceptors; LogBB = blood-brain distribution; ClogP = partitioning; ClogD = distribution at pKa; TPSA = polar surface area; HBD = hydrogen bond donors; CNS MPO = score for CNS penetration.

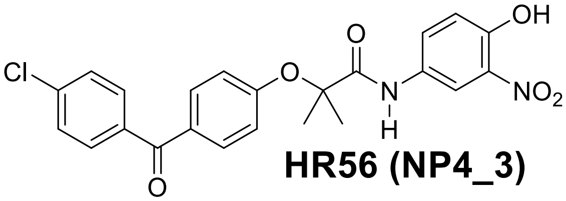

HR56 (NP4_3)

Calc.	Marvin 19.4		HLB	logS	PL	MPA	MSA	RF	HBA	LogBB
		T0							6	-0.79
ClogP	5.43	0								
ClogD	3.94	0.03								
MW	454.86	0.32								
TPSA	118.77	0.04								
HBD	2	0.5								
pKa	6.72	1								
CNS MPO	1.89									

HLB = hydrophilic-lipophilic balance; logS = water solubility; PL = polarizability(Å^3); Minimal Projection Area (Å^2); MSA = molecular surface area; RF = Refractivity; HBA = hydrogen bond acceptors; LogBB = blood-brain distribution; ClogP = partitioning; ClogD = distribution at pKa; TPSA = polar surface area; HBD = hydrogen bond donors; CNS MPO = score for CNS penetration.

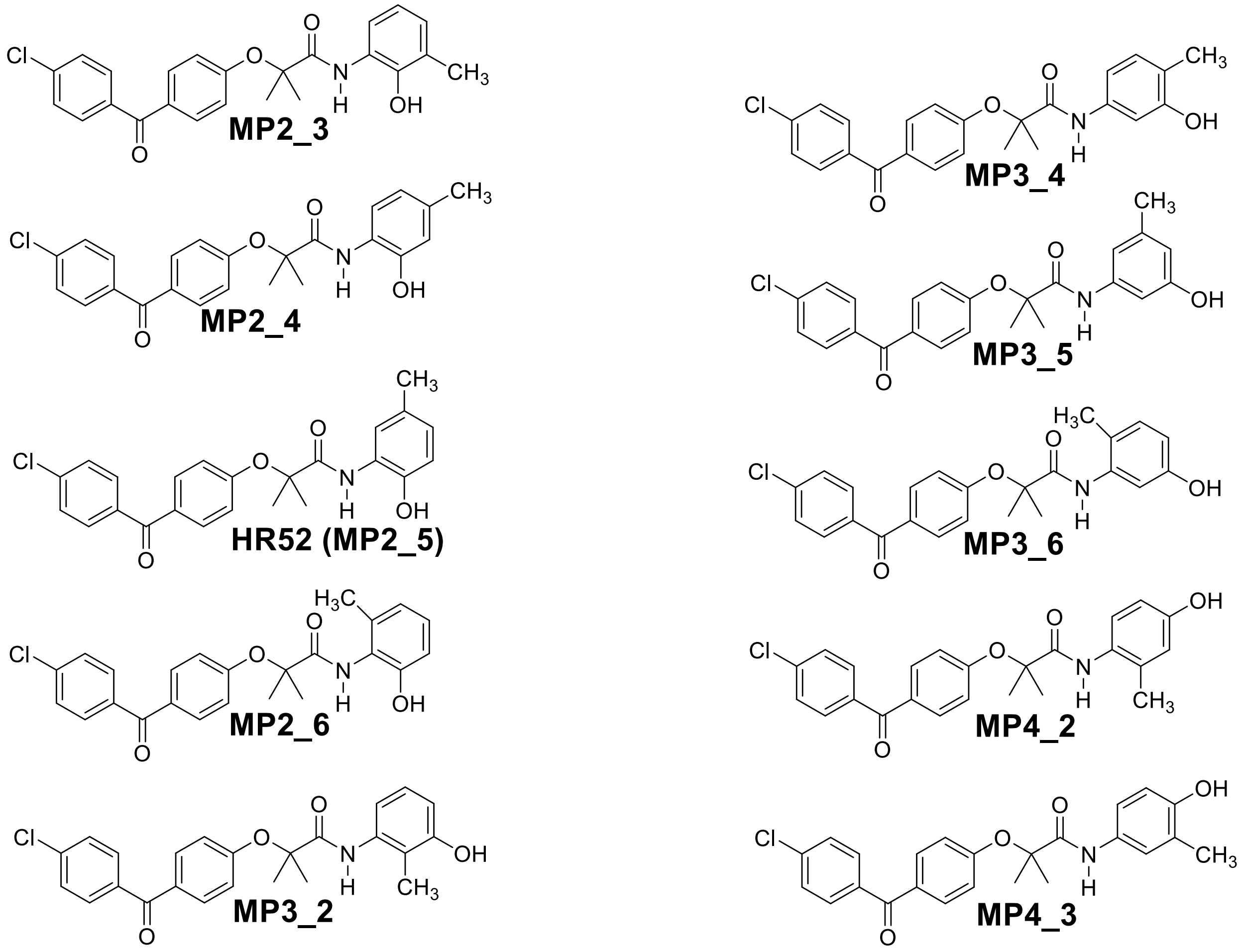

Scheme 4. Methyl hydroxyphenyl **BPA** derivatives (**MP** derivatives)

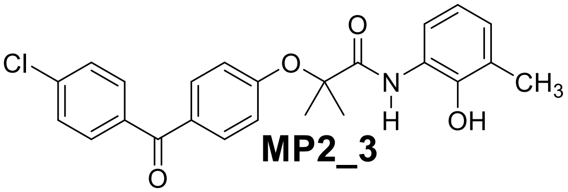

MP2_3

Calc.	Marvin 19.20		HLB	logS	PL	MPA	MSA	RF	HBA	LogBB
		T0	4.15	-6.85	45.05	45.48	592.48	118.49	4	-0.07
ClogP	6.00	0								
ClogD	5.99	0								
MW	423.89	0.54								
TPSA	75.63	1								
HBD	2	0.5								
pKa	9.13	0.43								
CNS MPO	2.47									

HLB = hydrophilic-lipophilic balance; logS = water solubility; PL = polarizability(Å^3); Minimal Projection Area (Å^2); MSA = molecular surface area; RF = Refractivity; HBA = hydrogen bond acceptors; LogBB = blood-brain distribution; ClogP = partitioning; ClogD = distribution at pKa; TPSA = polar surface area; HBD = hydrogen bond donors; CNS MPO = score for CNS penetration.

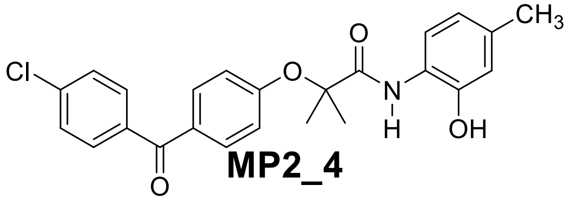

MP2_4

Calc.	Marvin 19.20		HLB	logS	PL	MPA	MSA	RF	HBA	LogBB
		T0	4.15	-5.05	45.05	44.92	592.12	118.49	4	-0.07
ClogP	6.00	0								
ClogD	5.99	0								
MW	423.89	0.54								
TPSA	75.63	1								
HBD	2	0.5								
pKa	8.86	0.57								
CNS MPO	2.61									

HLB = hydrophilic-lipophilic balance; logS = water solubility; PL = polarizability(Å^3); Minimal Projection Area (Å^2); MSA = molecular surface area; RF = Refractivity; HBA = hydrogen bond acceptors; LogBB = blood-brain distribution; ClogP = partitioning; ClogD = distribution at pKa; TPSA = polar surface area; HBD = hydrogen bond donors; CNS MPO = score for CNS penetration.

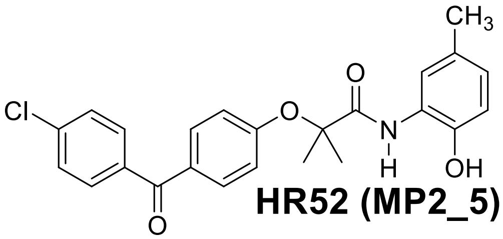

HR52 (MP2_5)

Calc.	Marvin 19.20		HLB	logS	PL	MPA	MSA	RF	HBA	LogBB
		T0	4.15	7.08	45.05	48.18	592.67	118.49	4	-0.07
ClogP	6.00	0								
ClogD	5.99	0								
MW	423.89	0.54								
TPSA	75.63	1								
HBD	2	0.5								
pKa	9.11	0.45								
CNS MPO	2.49									

HLB = hydrophilic-lipophilic balance; logS = water solubility; PL = polarizability(Å^3); Minimal Projection Area (Å^2); MSA = molecular surface area; RF = Refractivity; HBA = hydrogen bond acceptors; LogBB = blood-brain distribution; ClogP = partitioning; ClogD = distribution at pKa; TPSA = polar surface area; HBD = hydrogen bond donors; CNS MPO = score for CNS penetration.

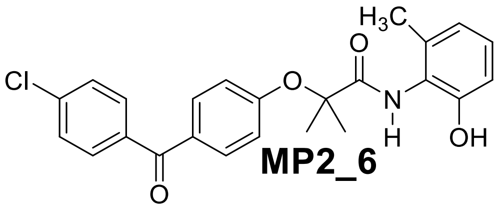

MP2_6

Calc.	Marvin 19.20		HLB	logS	PL	MPA	MSA	RF	HBA	LogBB
		T0	4.15	-6.83	45.05	50.64	591.08	118.49	4	-0.07
ClogP	6.00	0								
ClogD	5.97	0								
MW	423.89	0.54								
TPSA	75.63	1								
HBD	2	0.5								
pKa	8.89	0.55								
CNS MPO	2.59									

HLB = hydrophilic-lipophilic balance; logS = water solubility; PL = polarizability(Å^3); Minimal Projection Area (Å^2); MSA = molecular surface area; RF = Refractivity; HBA = hydrogen bond acceptors; LogBB = blood-brain distribution; ClogP = partitioning; ClogD = distribution at pKa; TPSA = polar surface area; HBD = hydrogen bond donors; CNS MPO = score for CNS penetration.

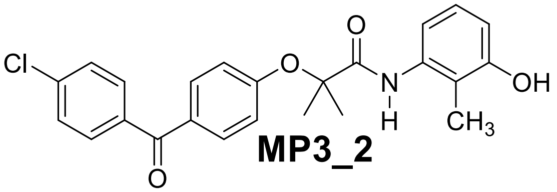

MP3_2

Calc.	Marvin 19.20		HLB	logS	PL	MPA	MSA	RF	HBA	LogBB
		T0	4.15	-6.85	45.05	48.76	592.13	118.49	4	-0.07
ClogP	6.00	0								
ClogD	5.99	0								
MW	423.89	0.54								
TPSA	75.63	1								
HBD	2	0.5								
pKa	9.64	0.18								
CNS MPO	2.22									

HLB = hydrophilic-lipophilic balance; logS = water solubility; PL = polarizability(Å^3); Minimal Projection Area (Å^2); MSA = molecular surface area; RF = Refractivity; HBA = hydrogen bond acceptors; LogBB = blood-brain distribution; ClogP = partitioning; ClogD = distribution at pKa; TPSA = polar surface area; HBD = hydrogen bond donors; CNS MPO = score for CNS penetration.

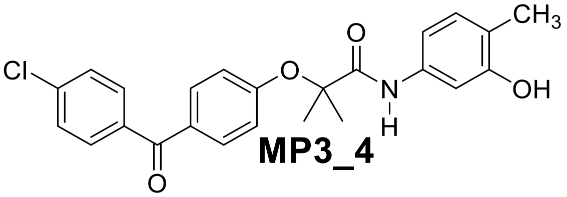

MP3_4

Calc.	Marvin 19.20		HLB	logS	PL	MPA	MSA	RF	HBA	LogBB
		T0	4.15	-6.85	45.05	47.17	593.65	118.49	4	-0.07
ClogP	6.00	0								
ClogD	5.99	0								
MW	423.89	0.54								
TPSA	75.63	1								
HBD	2	0.5								
pKa	9.56	0.22								
CNS MPO	2.26									

HLB = hydrophilic-lipophilic balance; logS = water solubility; PL = polarizability(Å^3); Minimal Projection Area (Å^2); MSA = molecular surface area; RF = Refractivity; HBA = hydrogen bond acceptors; LogBB = blood-brain distribution; ClogP = partitioning; ClogD = distribution at pKa; TPSA = polar surface area; HBD = hydrogen bond donors; CNS MPO = score for CNS penetration.

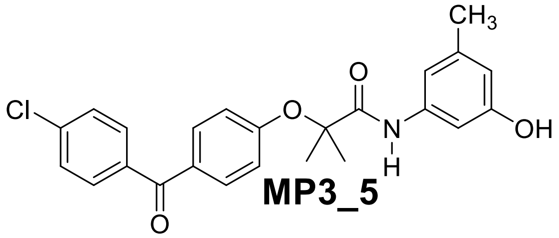

MP3_5

Calc.	Marvin 19.20		HLB	logS	PL	MPA	MSA	RF	HBA	LogBB
		T0	4.15	-7.01	45.05	50.36	594.29	118.49	4	-0.07
ClogP	6.00	0								
ClogD	5.99	0								
MW	423.89	0.54								
TPSA	75.63	1								
HBD	2	0.5								
pKa	9.35	0.33								
CNS MPO	2.37									

HLB = hydrophilic-lipophilic balance; logS = water solubility; PL = polarizability(Å^3); Minimal Projection Area (Å^2); MSA = molecular surface area; RF = Refractivity; HBA = hydrogen bond acceptors; LogBB = blood-brain distribution; ClogP = partitioning; ClogD = distribution at pKa; TPSA = polar surface area; HBD = hydrogen bond donors; CNS MPO = score for CNS penetration.

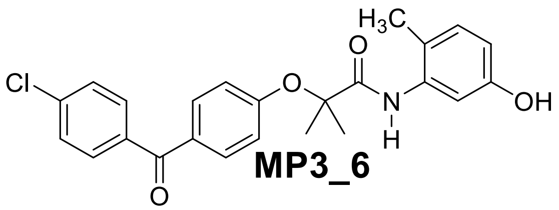

MP3_6

Calc.	Marvin 19.20		HLB	logS	PL	MPA	MSA	RF	HBA	LogBB
		T0	4.15	-6.85	45.05	46.59	593.14	118.49	4	-0.07
ClogP	6.00	0								
ClogD	5.99	0								
MW	423.89	0.54								
TPSA	75.63	1								
HBD	2	0.5								
pKa	9.63	0.18								
CNS MPO	2.22									

HLB = hydrophilic-lipophilic balance; logS = water solubility; PL = polarizability(Å^3); Minimal Projection Area (Å^2); MSA = molecular surface area; RF = Refractivity; HBA = hydrogen bond acceptors; LogBB = blood-brain distribution; ClogP = partitioning; ClogD = distribution at pKa; TPSA = polar surface area; HBD = hydrogen bond donors; CNS MPO = score for CNS penetration.

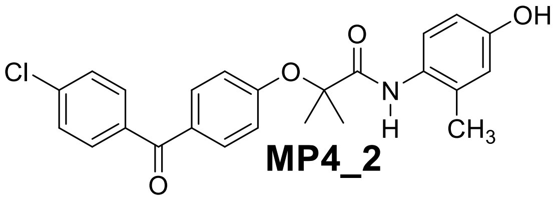

MP4_2

Calc.	Marvin 19.20		HLB	logS	PL	MPA	MSA	RF	HBA	LogBB
		T0	4.15	-6.85	45.05	50.75	593.60	118.49	4	-0.07
ClogP	6.00	0								
ClogD	5.99	0								
MW	423.89	0.54								
TPSA	75.63	1								
HBD	2	0.5								
pKa	9.55	0.22								
CNS MPO	2.26									

HLB = hydrophilic-lipophilic balance; logS = water solubility; PL = polarizability($Å^3$); Minimal Projection Area ($Å^2$); MSA = molecular surface area; RF = Refractivity; HBA = hydrogen bond acceptors; LogBB = blood-brain distribution; ClogP = partitioning; ClogD = distribution at pKa; TPSA = polar surface area; HBD = hydrogen bond donors; CNS MPO = score for CNS penetration.

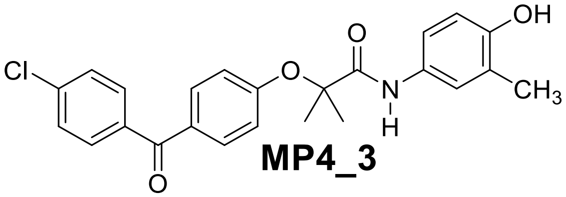

MP4_3

Calc.	Marvin 19.20		HLB	logS	PL	MPA	MSA	RF	HBA	LogBB
		T0	4.15	-6.86	45.05	47.40	593.41	118.49	4	-0.07
ClogP	6.00	0								
ClogD	6.00	0								
MW	423.89	0.54								
TPSA	75.63	1								
HBD	2	0.5								
pKa	9.79	0.11								
CNS MPO	2.15									

HLB = hydrophilic-lipophilic balance; logS = water solubility; PL = polarizability($Å^3$); Minimal Projection Area ($Å^2$); MSA = molecular surface area; RF = Refractivity; HBA = hydrogen bond acceptors; LogBB = blood-brain distribution; ClogP = partitioning; ClogD = distribution at pKa; TPSA = polar surface area; HBD = hydrogen bond donors; CNS MPO = score for CNS penetration.

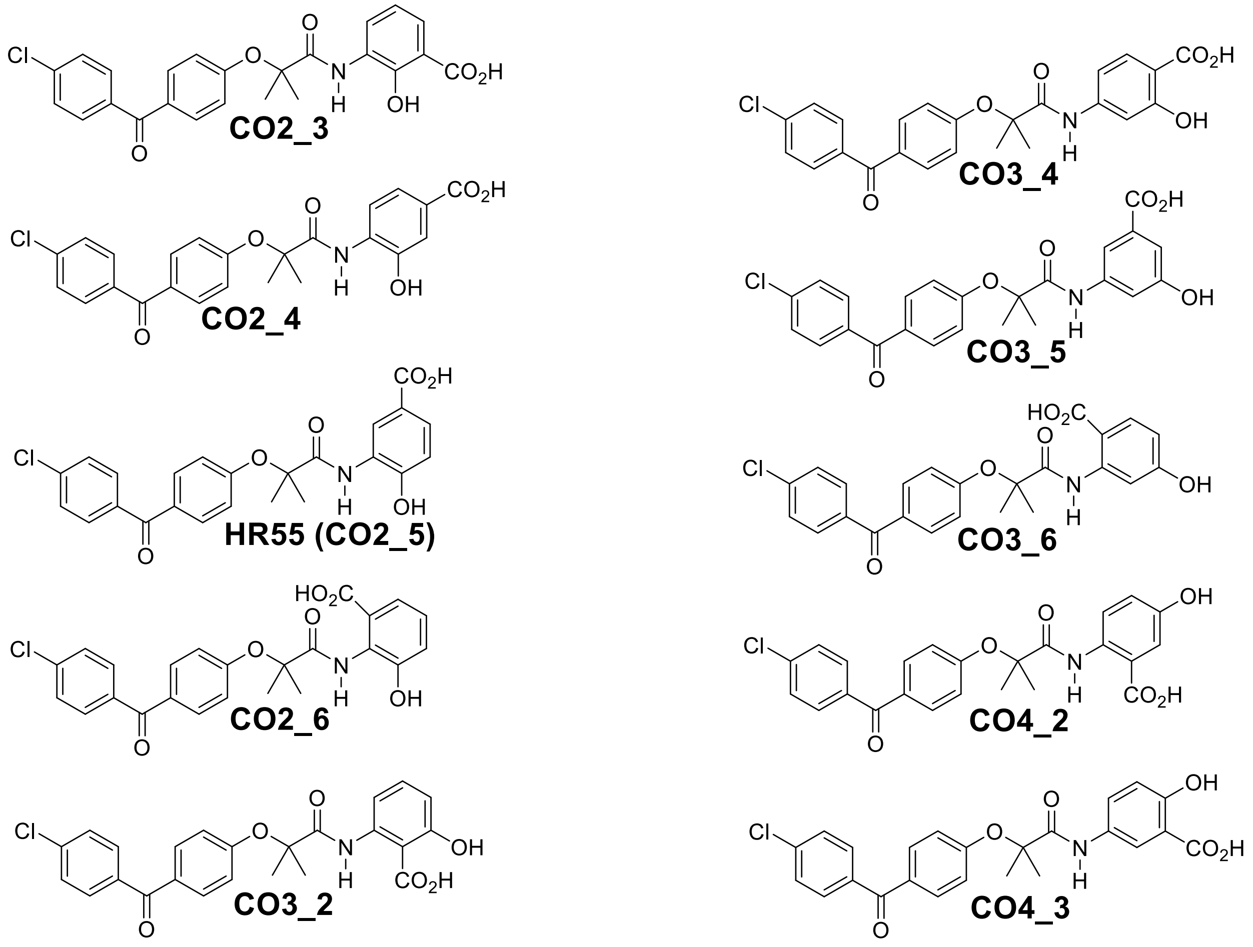

Scheme 5. Carboxylic acid hydroxyphenyl **BPA** derivatives (**CO** derivatives)

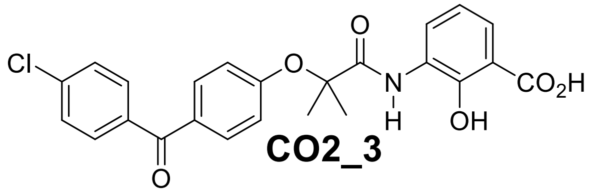

CO2_3

Calc.	Marvin 19.20		HLB	logS	PL	MPA	MSA	RF	HBA	LogBB
		T0	6.29	-2.61	45.66	45.77	595.56	120.71	6	-0.65
ClogP	5.80	0								
ClogD	2.31	0.85								
MW	453.88	0.33								
TPSA	112.93	0.24								
HBD	3	0.17								
pKa	2.64	1								
CNS MPO	2.59									

HLB = hydrophilic-lipophilic balance; logS = water solubility; PL = polarizability(Å^3); Minimal Projection Area (Å^2); MSA = molecular surface area; RF = Refractivity; HBA = hydrogen bond acceptors; LogBB = blood-brain distribution; ClogP = partitioning; ClogD = distribution at pKa; TPSA = polar surface area; HBD = hydrogen bond donors; CNS MPO = score for CNS penetration.

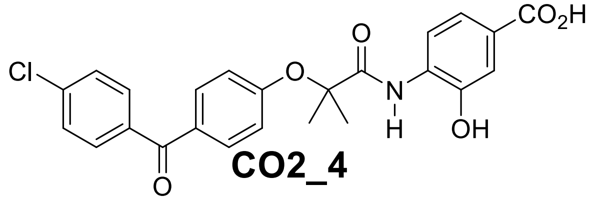

CO2_4

Calc.	Marvin 19.20		HLB	logS	PL	MPA	MSA	RF	HBA	LogBB
		T0	6.29	-2.98	45.65	46.63	596.93	120.71	6	-0.75
ClogP	5.15									
ClogD	1.93									
MW	453.88									
TPSA	112.93									
HBD	3									
pKa	3.95									
CNS MPO	2.74									

HLB = hydrophilic-lipophilic balance; logS = water solubility; PL = polarizability(Å^3); Minimal Projection Area (Å^2); MSA = molecular surface area; RF = Refractivity; HBA = hydrogen bond acceptors; LogBB = blood-brain distribution; ClogP = partitioning; ClogD = distribution at pKa; TPSA = polar surface area; HBD = hydrogen bond donors; CNS MPO = score for CNS penetration.

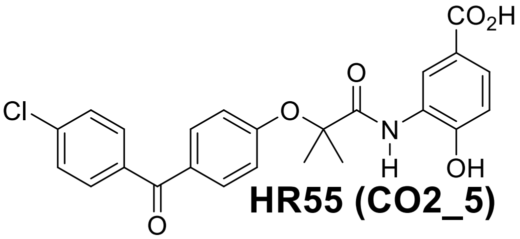

HR55 (CO2_5)

Calc.	Marvin 19.20		HLB	logS	PL	MPA	MSA	RF	HBA	LogBB
		T0	6.29	-3.26	45.65	52.16	597.96	120.71	6	-0.75
ClogP	5.15	0								
ClogD	2.04	0.98								
MW	453.88	0.33								
TPSA	112.93	0.24								
HBD	3	0.17								
pKa	4.23	1								
CNS MPO	2.72									

HLB = hydrophilic-lipophilic balance; logS = water solubility; PL = polarizability(Å^3); Minimal Projection Area (Å^2); MSA = molecular surface area; RF = Refractivity; HBA = hydrogen bond acceptors; LogBB = blood-brain distribution; ClogP = partitioning; ClogD = distribution at pKa; TPSA = polar surface area; HBD = hydrogen bond donors; CNS MPO = score for CNS penetration.

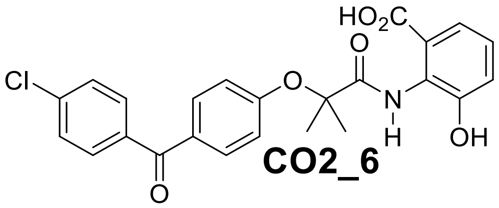

CO2_6

Calc.	Marvin 19.20		HLB	logS	PL	MPA	MSA	RF	HBA	LogBB
		T0	6.29	-2.43	45.65	53.49	595.63	120.71	6	-0.65
ClogP	5.80	0								
ClogD	2.41	0.79								
MW	453.88	0.33								
TPSA	112.93	0.24								
HBD	3	0.17								
pKa	3.34	1								
CNS MPO	2.53									

HLB = hydrophilic-lipophilic balance; logS = water solubility; PL = polarizability(Å^3); Minimal Projection Area (Å^2); MSA = molecular surface area; RF = Refractivity; HBA = hydrogen bond acceptors; LogBB = blood-brain distribution; ClogP = partitioning; ClogD = distribution at pKa; TPSA = polar surface area; HBD = hydrogen bond donors; CNS MPO = score for CNS penetration.

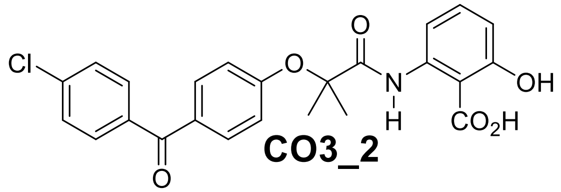

CO3_2

Calc.	Marvin 19.20		HLB	logS	PL	MPA	MSA	RF	HBA	LogBB
		T0	6.29	-1.25	45.66	54.17	596.61	120.71	6	-0.55
ClogP	6.45	0								
ClogD	2.93	0.53								
MW	453.88	0.33								
TPSA	112.93	0.24								
HBD	3	0.17								
pKa	2.27	1								
CNS MPO	2.27									

HLB = hydrophilic-lipophilic balance; logS = water solubility; PL = polarizability(Å^3); Minimal Projection Area (Å^2); MSA = molecular surface area; RF = Refractivity; HBA = hydrogen bond acceptors; LogBB = blood-brain distribution; ClogP = partitioning; ClogD = distribution at pKa; TPSA = polar surface area; HBD = hydrogen bond donors; CNS MPO = score for CNS penetration.

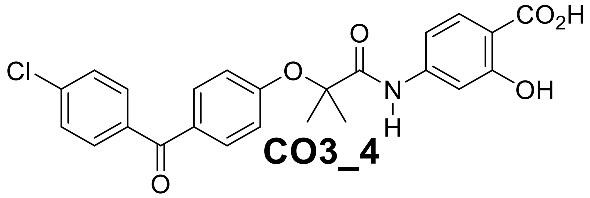

CO3_4

Calc.	Marvin 19.20		HLB	logS	PL	MPA	MSA	RF	HBA	LogBB
		T0	6.29	-1.98	45.65	44.91	597.97	120.71	6	-0.65
ClogP	5.80	0								
ClogD	2.32	0.84								
MW	453.88	0.33								
TPSA	112.93	0.24								
HBD	3	0.17								
pKa	2.87	1								
CNS MPO	2.58									

HLB = hydrophilic-lipophilic balance; logS = water solubility; PL = polarizability(Å^3); Minimal Projection Area (Å^2); MSA = molecular surface area; RF = Refractivity; HBA = hydrogen bond acceptors; LogBB = blood-brain distribution; ClogP = partitioning; ClogD = distribution at pKa; TPSA = polar surface area; HBD = hydrogen bond donors; CNS MPO = score for CNS penetration.

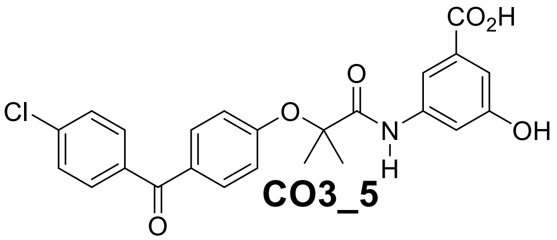

CO3_5

Calc.	Marvin 19.20		HLB	logS	PL	MPA	MSA	RF	HBA	LogBB
		T0	6.29	-3.78	45.65	51.61	599.40	120.71	6	-0.75
ClogP	5.15	0								
ClogD	1.88	1								
MW	453.88	0.33								
TPSA	112.93	0.24								
HBD	3	0.17								
pKa	3.69	1								
CNS MPO	2.74									

HLB = hydrophilic-lipophilic balance; logS = water solubility; PL = polarizability(Å^3); Minimal Projection Area (Å^2); MSA = molecular surface area; RF = Refractivity; HBA = hydrogen bond acceptors; LogBB = blood-brain distribution; ClogP = partitioning; ClogD = distribution at pKa; TPSA = polar surface area; HBD = hydrogen bond donors; CNS MPO = score for CNS penetration.

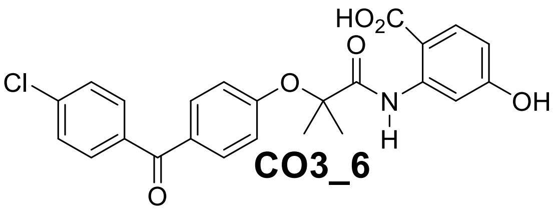

CO3_6

Calc.	Marvin 19.20		HLB	logS	PL	MPA	MSA	RF	HBA	LogBB
		T0	6.29	-2.96	45.65	51.97	597.32	120.71	6	-0.65
ClogP	5.80	0								
ClogD	3.59	0.21								
MW	453.88	0.33								
TPSA	112.93	0.24								
HBD	3	0.17								
pKa	3.87	1								
CNS MPO	1.95									

HLB = hydrophilic-lipophilic balance; logS = water solubility; PL = polarizability(Å^3); Minimal Projection Area (Å^2); MSA = molecular surface area; RF = Refractivity; HBA = hydrogen bond acceptors; LogBB = blood-brain distribution; ClogP = partitioning; ClogD = distribution at pKa; TPSA = polar surface area; HBD = hydrogen bond donors; CNS MPO = score for CNS penetration.

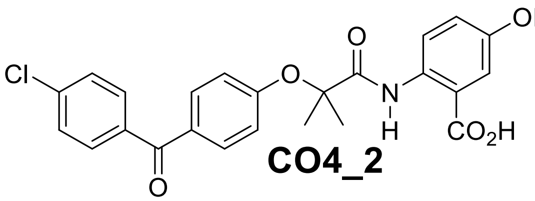

CO4_2

Calc.	Marvin 19.20		HLB	logS	PL	MPA	MSA	RF	HBA	LogBB
		T0	6.29	-2.38	45.65	54.80	597.78	120.71	6	-0.65
ClogP	5.80	0								
ClogD	2.38	0.81								
MW	453.88	0.33								
TPSA	112.93	0.24								
HBD	3	0.17								
pKa	3.31	1								
CNS MPO	2.55									

HLB = hydrophilic-lipophilic balance; logS = water solubility; PL = polarizability(Å^3); Minimal Projection Area (Å^2); MSA = molecular surface area; RF = Refractivity; HBA = hydrogen bond acceptors; LogBB = blood-brain distribution; ClogP = partitioning; ClogD = distribution at pKa; TPSA = polar surface area; HBD = hydrogen bond donors; CNS MPO = score for CNS penetration.

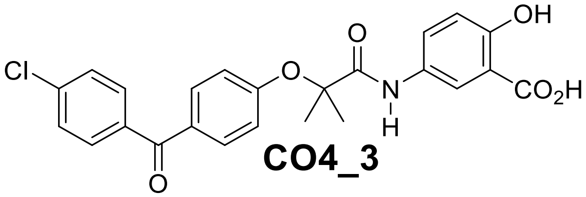

CO4_3

Calc.	Marvin 19.20		HLB	logS	PL	MPA	MSA	RF	HBA	LogBB
		T0	6.29	-1.71	45.65	51.80	597.59	120.71	6	-0.65
ClogP	5.80	0								
ClogD	2.29	0.86								
MW	453.88	0.33								
TPSA	112.93	0.24								
HBD	3	0.17								
pKa	2.61	1								
CNS MPO	2.60									

HLB = hydrophilic-lipophilic balance; logS = water solubility; PL = polarizability(Å^3); Minimal Projection Area (Å^2); MSA = molecular surface area; RF = Refractivity; HBA = hydrogen bond acceptors; LogBB = blood-brain distribution; ClogP = partitioning; ClogD = distribution at pKa; TPSA = polar surface area; HBD = hydrogen bond donors; CNS MPO = score for CNS penetration.

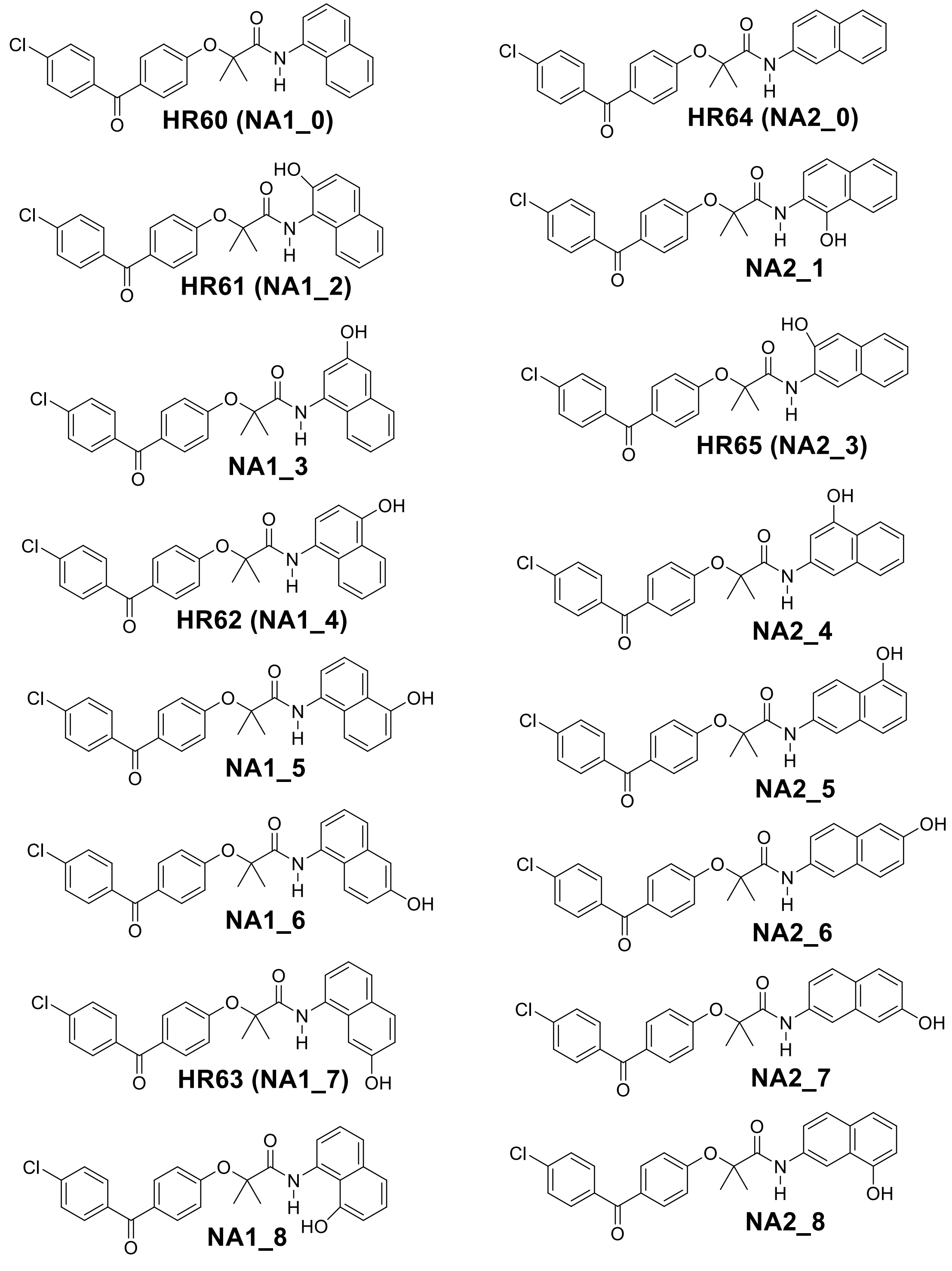

Scheme 6. Hydroxynaphthalene **BPA** derivatives (**NA** derivatives)

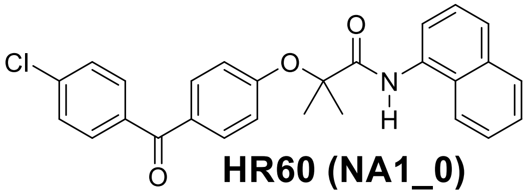

HR60 (NA1_0)

Calc.	Marvin 19.4		HLB	logS	PL	MPA	MSA	RF	HBA	LogBB
		T0	3.04	-8.79	50.20	50.10	612.55	127.92	3	0.35
ClogP	6.78	0								
ClogD	6.78	0								
MW	443.92	0.4								
TPSA	55.40	1								
HBD	1	0.83								
pKa	12.22	0								
CNS MPO	2.23									

HLB = hydrophilic-lipophilic balance; logS = water solubility; PL = polarizability(Å^3); Minimal Projection Area (Å^2); MSA = molecular surface area; RF = Refractivity; HBA = hydrogen bond acceptors; LogBB = blood-brain distribution; ClogP = partitioning; ClogD = distribution at pKa; TPSA = polar surface area; HBD = hydrogen bond donors; CNS MPO = score for CNS penetration.

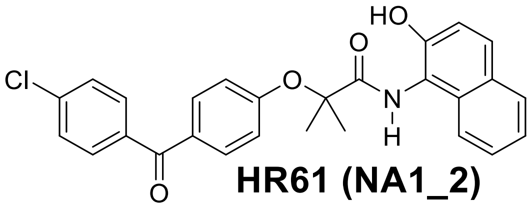

HR61 (NA1_2)

Calc.	Marvin 19.4		HLB	logS	PL	MPA	MSA	RF	HBA	LogBB
		T0	3.07	-8.28	50.79	48.22	622.51	129.90	4	0.01
ClogP	6.48	0								
ClogD	6.41	0								
MW	459.92	0.29								
TPSA	75.63	1								
HBD	2	0.5								
pKa	8.56	0.72								
CNS MPO	2.51									

HLB = hydrophilic-lipophilic balance; PL = polarizability(Å^3); Minimal Projection Area (Å^2); RF = Refractivity

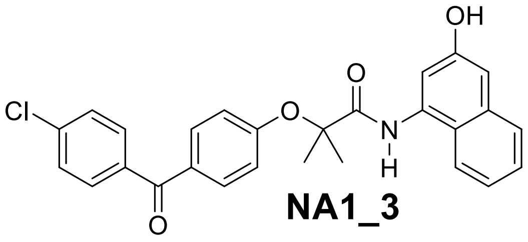

NA1_3

Calc.	Marvin 19.20		HLB	logS	PL	MPA	MSA	RF	HBA	LogBB
		T0	3.07	-8.33	50.79	53.01	623.53	129.90	4	0.004
ClogP	6.48	0								
ClogD	6.48	0								
MW	459.92	0.29								
PSA	75.63	1								
HBD	2	0.5								
pKa	9.01	1								
CNS MPO	2.29									

ClogP = calculated partitioning; HLB = hydrophilic-lipophilic balance; logS = water solubility; PL = polarizability(Å^3); RF = Refractivity; HBD = hydrogen bond donor at pH = 7; HBA = hydrogen bond acceptor at pH =7; logBB = calculated blood-brain partition; PSA = Polar surface area (Å^2); MPA = Minimal projection area (Å^2); LogS = Aqueous solubility (mg/ml); MPO = Central nervous system multiparameter optimization (CNS MPO).

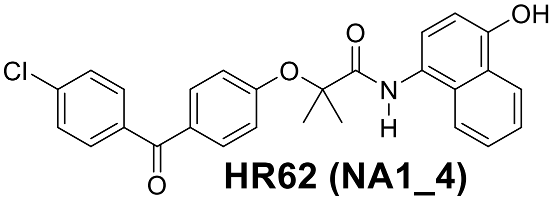

HR62 (NA1_4)

Calc.	Marvin 19.20		HLB	logS	PL	MPA	MSA	RF	HBA	LogBB
		T0	3.07	-8.33	50.79	50.50	623.01	129.90	4	0.004
ClogP	6.48	0								
ClogD	6.48	0								
MW	459.92	0.29								
PSA	75.63	1								
HBD	2	0.5								
pKa	9.06	0.47								
CNS MPO	2.26									

ClogP = calculated partitioning; HLB = hydrophilic-lipophilic balance; logS = water solubility; PL = polarizability(Å^3); RF = Refractivity; HBD = hydrogen bond donor at pH = 7; HBA = hydrogen bond acceptor at pH =7; logBB = calculated blood-brain partition; PSA = Polar surface area (Å^2); MPA = Minimal projection area (Å^2); LogS = Aqueous solubility (mg/ml); MPO = Central nervous system multiparameter optimization (CNS MPO).

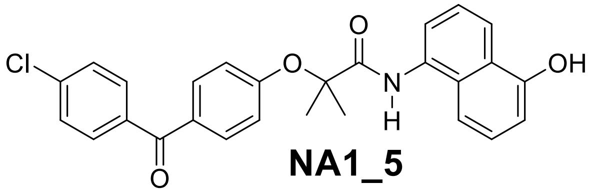

NA1_5

Calc.	Marvin 19.20		HLB	logS	PL	MPA	MSA	RF	HBA	LogBB
		T0	3.07	-8.33	50.79	50.07	621.47	129.90	4	0.004
ClogP	6.48	0								
ClogD	6.48	0								
MW	459.92	0.29								
PSA	75.63	1								
HBD	2	0.5								
pKa	9.54	0.23								
CNS MPO	2.02									

ClogP = calculated partitioning; HLB = hydrophilic-lipophilic balance; logS = water solubility; PL = polarizability(Å^3); RF = Refractivity; HBD = hydrogen bond donor at pH = 7; HBA = hydrogen bond acceptor at pH =7; logBB = calculated blood-brain partition; PSA = Polar surface area (Å^2); MPA = Minimal projection area (Å^2); LogS = Aqueous solubility (mg/ml); MPO = Central nervous system multiparameter optimization (CNS MPO).

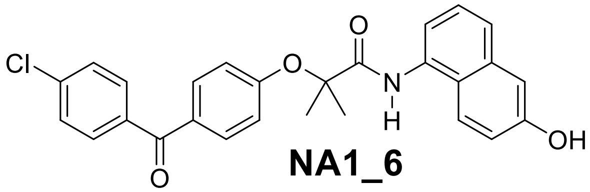

NA1_6

Calc.	Marvin 19.20		HLB	logS	PL	MPA	MSA	RF	HBA	LogBB
		T0	3.07	-8.30	50.78	51.67	622.43	129.90	4	0.004
ClogP	6.48	0								
ClogD	6.48	0								
MW	459.92	0.29								
PSA	75.63	1								
HBD	2	0.5								
pKa	9.76	0.23								
CNS MPO	1.91									

ClogP = calculated partitioning; HLB = hydrophilic-lipophilic balance; logS = water solubility; PL = polarizability(Å^3); RF = Refractivity; HBD = hydrogen bond donor at pH = 7; HBA = hydrogen bond acceptor at pH =7; logBB = calculated blood-brain partition; PSA = Polar surface area (Å^2); MPA = Minimal projection area (Å^2); LogS = Aqueous solubility (mg/ml); MPO = Central nervous system multiparameter optimization (CNS MPO).

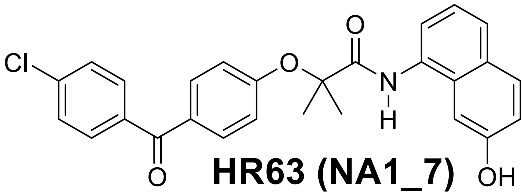

HR63 (NA1_7)

Calc.	Marvin 19.20		HLB	logS	PL	MPA	MSA	RF	HBA	LogBB
		T0	3.07	-8.30	50.78	53.68	623.34	129.901.9	4	0.004
ClogP	6.48	0								
ClogD	6.48	0								
MW	459.92	0.29								
PSA	75.63	1								
HBD	2	0.5								
pKa	9.71	0.14								
CNS MPO	1.93									

ClogP = calculated partitioning; HLB = hydrophilic-lipophilic balance; logS = water solubility; PL = polarizability(Å^3); RF = Refractivity; HBD = hydrogen bond donor at pH = 7; HBA = hydrogen bond acceptor at pH =7; logBB = calculated blood-brain partition; PSA = Polar surface area (Å^2); MPA = Minimal projection area (Å^2); LogS = Aqueous solubility (mg/ml); MPO = Central nervous system multiparameter optimization (CNS MPO).

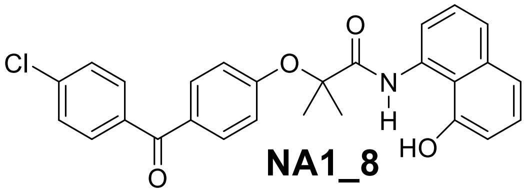

NA1_8

Calc.	Marvin 19.20		HLB	logS	PL	MPA	MSA	RF	HBA	LogBB
		T0	3.07	-8.30	50.79	50.57	621.76	129.90	4	0.004
ClogP	6.48	0								
ClogD	6.48	0								
MW	459.92	0.29								
PSA	75.63	1								
HBD	2	0.5								
pKa	9.31	0.34								
CNS MPO	2.13									

ClogP = calculated partitioning; HLB = hydrophilic-lipophilic balance; logS = water solubility; PL = polarizability(Å^3); RF = Refractivity; HBD = hydrogen bond donor at pH = 7; HBA = hydrogen bond acceptor at pH =7; logBB = calculated blood-brain partition; PSA = Polar surface area (Å^2); MPA = Minimal projection area (Å^2); LogS = Aqueous solubility (mg/ml); MPO = Central nervous system multiparameter optimization (CNS MPO).

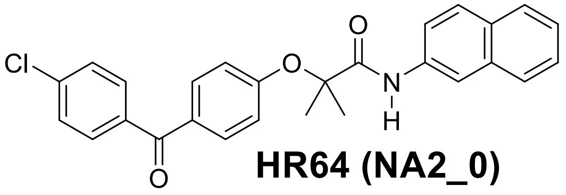

HR64 (NA2_0)

Calc.	Marvin 19.20		HLB	logS	PL	MPA	MSA	RF	HBA	LogBB
		T0	3.04	-8.79	50.20	50.72	613.44	127.92	3	0.35
ClogP	6.78	0								
ClogD	6.78	0								
MW	443.93	0.4								
PSA	55.40	1								
HBD	1	0.83								
pKa	12.64	0								
CNS MPO	2.23									

ClogP = calculated partitioning; HLB = hydrophilic-lipophilic balance; logS = water solubility; PL = polarizability(Å^3); RF = Refractivity; HBD = hydrogen bond donor at pH = 7; HBA = hydrogen bond acceptor at pH =7; logBB = calculated blood-brain partition; PSA = Polar surface area (Å^2); MPA = Minimal projection area (Å^2); LogS = Aqueous solubility (mg/ml); MPO = Central nervous system multiparameter optimization (CNS MPO).

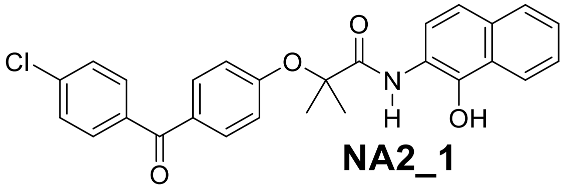

NA2_1

Calc.	Marvin 19.20		HLB	logS	PL	MPA	MSA	RF	HBA	LogBB
		T0	3.07	-8.21	50.79	48.37	622.91	129.90	4	0.004
ClogP	6.48	0								
ClogD	6.48	0								
MW	459.92	0.29								
PSA	75.63	1								
HBD	2	0.5								
pKa	8.37	0.82								
CNS MPO	2.61									

ClogP = calculated partitioning; HLB = hydrophilic-lipophilic balance; logS = water solubility; PL = polarizability(Å^3); RF = Refractivity; HBD = hydrogen bond donor at pH = 7; HBA = hydrogen bond acceptor at pH =7; logBB = calculated blood-brain partition; PSA = Polar surface area (Å^2); MPA = Minimal projection area (Å^2); LogS = Aqueous solubility (mg/ml); MPO = Central nervous system multiparameter optimization (CNS MPO).

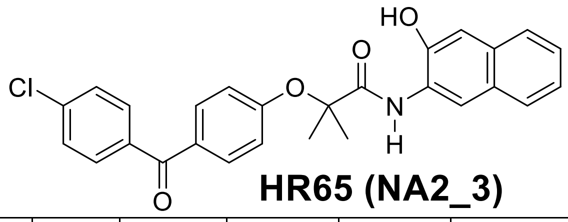

HR65 (NA2_3)

Calc.	Marvin 19.20		HLB	logS	PL	MPA	MSA	RF	HBA	LogBB
		T0	3.07	-8.27	50.78	50.52	623.20	129.90	4	0.004
ClogP	6.48	0								
ClogD	6.42	0								
MW	459.92	0.29								
PSA	75.63	1								
HBD	2	0.5								
pKa	8.53	0.74								
MPO	2.53									

ClogP = calculated partitioning; HLB = hydrophilic-lipophilic balance; logS = water solubility; PL = polarizability(Å^3); RF = Refractivity; HBD = hydrogen bond donor at pH = 7; HBA = hydrogen bond acceptor at pH =7; logBB = calculated blood-brain partition; PSA = Polar surface area (Å^2); MPA = Minimal projection area (Å^2); LogS = Aqueous solubility (mg/ml); MPO = Central nervous system multiparameter optimization (CNS MPO).

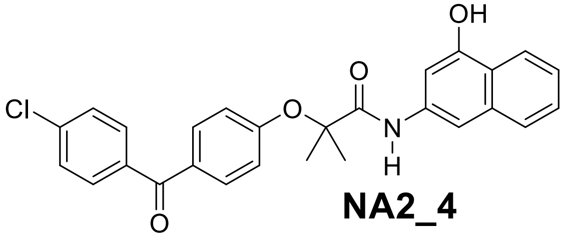

NA2_4

Calc.	Marvin 19.20		HLB	logS	PL	MPA	MSA	RF	HBA	LogBB
		T0	3.07	-8.05	50.79	51.20	624.70	129.90	4	0.004
ClogP	6.48	0								
ClogD	6.48	0								
MW	459.92	0.29								
PSA	75.63	1								
HBD	2	0.5								
pKa	8.82	0.59								
CNS MPO	2.38									

ClogP = calculated partitioning; HLB = hydrophilic-lipophilic balance; logS = water solubility; PL = polarizability(Å^3); RF = Refractivity; HBD = hydrogen bond donor at pH = 7; HBA = hydrogen bond acceptor at pH =7; logBB = calculated blood-brain partition; PSA = Polar surface area (Å^2); MPA = Minimal projection area (Å^2); LogS = Aqueous solubility (mg/ml); MPO = Central nervous system multiparameter optimization (CNS MPO).

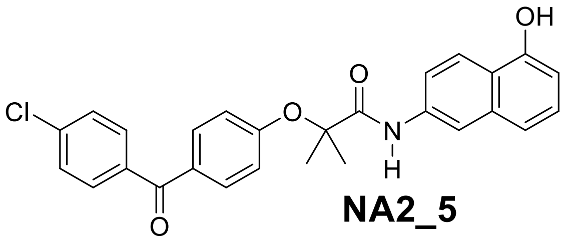

NA2_5

Calc.	Marvin 19.20		HLB	logS	PL	MPA	MSA	RF	HBA	LogBB
		T0	3.07	-8.28	50.79	50.10	624.00	129.90	4	0.004
ClogP	6.48	0								
ClogD	6.48	0								
MW	459.92	0.29								
PSA	75.63	1								
HBD	2	0.5								
pKa	9.63	0.18								
CNS MPO	1.97									

ClogP = calculated partitioning; HLB = hydrophilic-lipophilic balance; logS = water solubility; PL = polarizability(Å^3); RF = Refractivity; HBD = hydrogen bond donor at pH = 7; HBA = hydrogen bond acceptor at pH =7; logBB = calculated blood-brain partition; PSA = Polar surface area (Å^2); MPA = Minimal projection area (Å^2); LogS = Aqueous solubility (mg/ml); MPO = Central nervous system multiparameter optimization (CNS MPO).

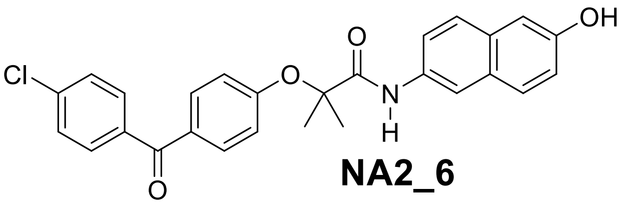

NA2_6

Calc.	Marvin 19.20		HLB	logS	PL	MPA	MSA	RF	HBA	LogBB
		T0	3.07	-8.10	50.78	49.47	625.21	129.90	4	0.004
ClogP	6.48	0								
ClogD	6.48	0								
MW	459.92	0.29								
PSA	75.63	1								
HBD	2	0.5								
pKa	9.79	0.11								
CNS MPO	1.90									

ClogP = calculated partitioning; HLB = hydrophilic-lipophilic balance; logS = water solubility; PL = polarizability(Å^3); RF = Refractivity; HBD = hydrogen bond donor at pH = 7; HBA = hydrogen bond acceptor at pH =7; logBB = calculated blood-brain partition; PSA = Polar surface area (Å^2); MPA = Minimal projection area (Å^2); LogS = Aqueous solubility (mg/ml); MPO = Central nervous system multiparameter optimization (CNS MPO).

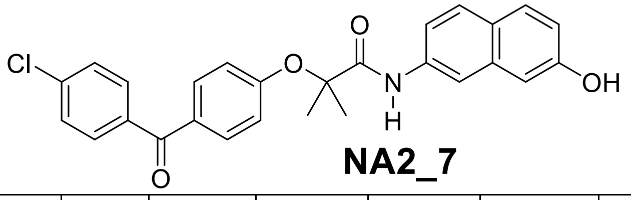

NA2_7

Calc.	Marvin 19.20		HLB	logS	PL	MPA	MSA	RF	HBA	LogBB
		T0	3.07	-8.11	50.78	52.36	625.42	129.90	4	0.004
ClogP	6.48	0								
ClogD	6.48	0								
MW	459.92	0.29								
PSA	75.63	1								
HBD	2	0.5								
pKa	9.77	0.12								
CNS MPO	1.91									

ClogP = calculated partitioning; HLB = hydrophilic-lipophilic balance; logS = water solubility; PL = polarizability(Å^3); RF = Refractivity; HBD = hydrogen bond donor at pH = 7; HBA = hydrogen bond acceptor at pH =7; logBB = calculated blood-brain partition; PSA = Polar surface area (Å^2); MPA = Minimal projection area (Å^2); LogS = Aqueous solubility (mg/ml); MPO = Central nervous system multiparameter optimization (CNS MPO).

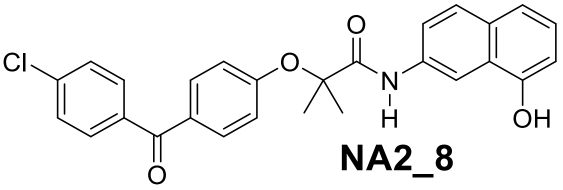

NA2_8

Calc.	Marvin 19.20		HLB	logS	PL	MPA	MSA	RF	HBA	LogBB
		T0	3.07	-8.33	50.79	49.87	623.98	129.90	4	0.004
ClogP	6.48	0								
ClogD	6.48	0								
MW	459.92	0.29								
PSA	75.63	1								
HBD	2	0.5								
pKa	9.54	0.23								
CNS MPO	2.02									

ClogP = calculated partitioning; HLB = hydrophilic-lipophilic balance; logS = water solubility; PL = polarizability(Å^3); RF = Refractivity; HBD = hydrogen bond donor at pH = 7; HBA = hydrogen bond acceptor at pH =7; logBB = calculated blood-brain partition; PSA = Polar surface area (Å^2); MPA = Minimal projection area (Å^2); LogS = Aqueous solubility (mg/ml); MPO = Central nervous system multiparameter optimization (CNS MPO).

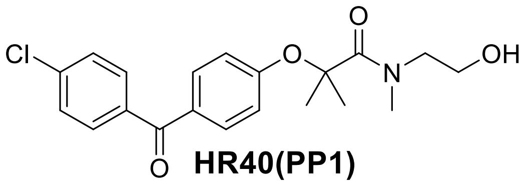

HR40(PP1)

Calc.	Marvin 19.20		HLB	logS	PL	MPA	MSA	RF	HBA	LogBB
			7.54	-5.09	39.25	51.69	551.21	101.10	4	-0.35
ClogP	3.31	0.85								
ClogD	3.31	0.35								
MW	375.85	0.89								
PSA	66.84	1								
HBD	1	0.83								
pKa	15.57	0								
CNS MPO	3.92									

ClogP = calculated partitioning; HLB = hydrophilic-lipophilic balance; logS = water solubility; PL = polarizability(Å^3); RF = Refractivity; HBD = hydrogen bond donor at pH = 7; HBA = hydrogen bond acceptor at pH =7; logBB = calculated blood-brain partition; PSA = Polar surface area (Å^2); MPA = Minimal projection area (Å^2); LogS = Aqueous solubility (mg/ml); MPO = Central nervous system multiparameter optimization (CNS MPO).

Known drugs use for ClogBB calibration

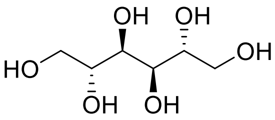

Mannitol
LogBB = - 2.51
ClogBB = - 2.22

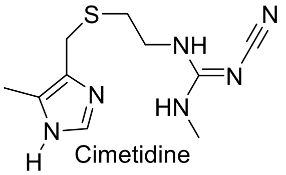

Cimetidine
LogBB = - 1.42
ClogBB = - 1.19

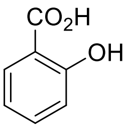

Salicylate
LogBB = - 1.10
ClogBB = - 0.61

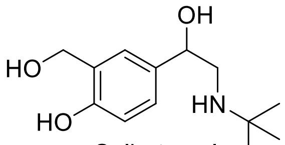

Salbutamol
LogBB = - 1.03
ClogBB = - 0.80

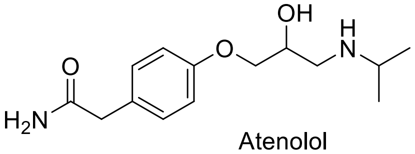

Atenolol
LogBB = - 0.87
ClogBB = - 1.05

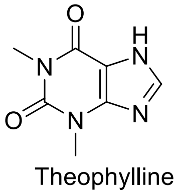

Theophylline
LogBB = - 0.29
ClogBB = - 1.00

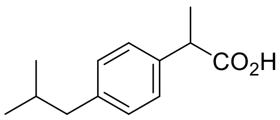

Ibuprofen
LogBB = - 0.18
ClogBB = 0.17

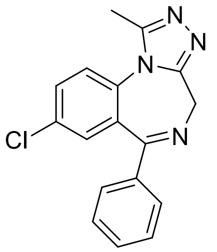

Alprazolam
LogBB = 0.02
ClogBB = -0.04

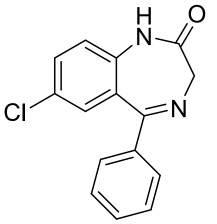

Nordazepam
LogBB = 0.50
ClogBB =0.01

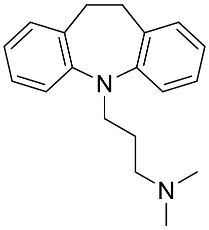

Imipramine
LogBB = 0.83
ClogBB = 0.69

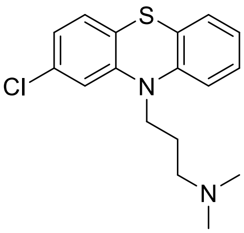

Chloropromazine
LogBB = 1.06
ClogBB = 0.73

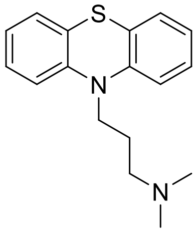

Promazine
LogBB = 1.23
ClogBB = 0.64

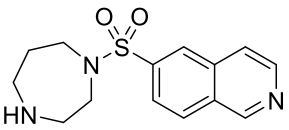

Fasudil
ClogBB =-0.73

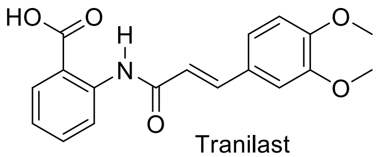

Tranilast
ClogBB =-0.57

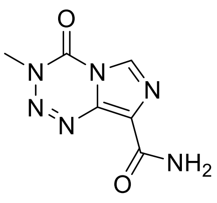

Temozolomine
ClogBB =-1.47

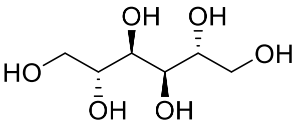

Mannitol
LogBB = - 2.51
ClogBB = - 2.22

Calc.	Marvin 19.20		HLB	logS	PL	MPA	MSA	RF	HBA	LogBB
			15.52	1.16	15.78	31.17	264.97	38.40	6	-2.22
ClogP	-3.73	1								
ClogD	-3.73	1								
MW	182.17	1								
TPSA	121.38	0								
HBD	6	0								
pKa	12.59	0								
CNS MPO	3.00									

HLB = hydrophilic-lipophilic balance; logS = water solubility; PL = polarizability(Å^3); Minimal Projection Area (Å^2); MSA = molecular surface area; RF = Refractivity; HBA = hydrogen bond acceptors; LogBB = blood-brain distribution; ClogP = partitioning; ClogD = distribution at pKa; TPSA = polar surface area; HBD = hydrogen bond donors; CNS MPO = score for CNS penetration.

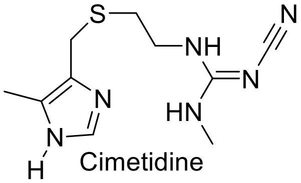

Cimetidine
LogBB = - 1.42
ClogBB = - 1.19

Calc.	Marvin 19.20		HLB	logS	PL	MPA	MSA	RF	HBA	LogBB
			17.86	-1.62	25.89	40.62	372.97	70.32	5	-1.19
ClogP	-0.11	1								
ClogD	-0.24	1								
MW	252.34	1								
TPSA	88.89	0.67								
HBD	3	0.17								
pKa	14.16	0								
CNS MPO	3.84									

HLB = hydrophilic-lipophilic balance; logS = water solubility; PL = polarizability(Å^3); Minimal Projection Area (Å^2); MSA = molecular surface area; RF = Refractivity; HBA = hydrogen bond acceptors; LogBB = blood-brain distribution; ClogP = partitioning; ClogD = distribution at pKa; TPSA = polar surface area; HBD = hydrogen bond donors; CNS MPO = score for CNS penetration.

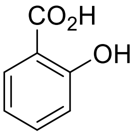

Salicylate
LogBB = - 1.10
ClogBB = - 0.61

Calc.	Marvin 19.20		HLB	logS	PL	MPA	MSA	RF	HBA	LogBB
			8.77	0.93	13.30	23.52	182.25	35.30	3	-0.61
ClogP	0.65	1								
ClogD	-1.51	1								
MW	138.12	1								
TPSA	57.53	1								
HBD	2	0.5								
pKa	2.79	1								
CNS MPO	5.5									

HLB = hydrophilic-lipophilic balance; logS = water solubility; PL = polarizability($Å^3$); Minimal Projection Area ($Å^2$); MSA = molecular surface area; RF = Refractivity; HBA = hydrogen bond acceptors; LogBB = blood-brain distribution; ClogP = partitioning; ClogD = distribution at pKa; TPSA = polar surface area; HBD = hydrogen bond donors; CNS MPO = score for CNS penetration.

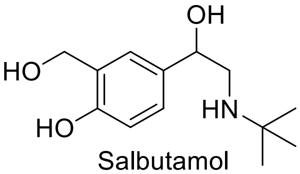

Salbutamol
LogBB = - 1.03
ClogBB = - 0.80

Calc.	Marvin 19.20		HLB	logS	PL	MPA	MSA	RF	HBA	LogBB
			14.06	0.93	26.58	44.36	406.23	67.87	4	-0.80
ClogP	0.88	1								
ClogD	-1.08	1								
MW	239.32	1								
TPSA	72.72	1								
HBD	4	0								
pKa	10.12	0								
CNS MPO	4.0									

HLB = hydrophilic-lipophilic balance; logS = water solubility; PL = polarizability($Å^3$); Minimal Projection Area ($Å^2$); MSA = molecular surface area; RF = Refractivity; HBA = hydrogen bond acceptors; LogBB = blood-brain distribution; ClogP = partitioning; ClogD = distribution at pKa; TPSA = polar surface area; HBD = hydrogen bond donors; CNS MPO = score for CNS penetration.

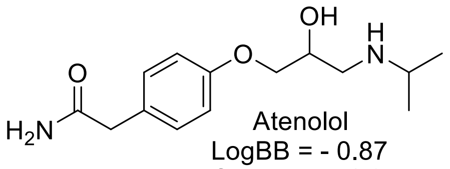

Atenolol
LogBB = - 0.87
ClogBB = - 1.05

Calc.	Marvin 19.20		HLB	logS	PL	MPA	MSA	RF	HBA	LogBB
			15.10	0.31	29.09	42.93	440.22	73.51	4	-1.05
ClogP	0.43	1								
ClogD	-1.81	1								
MW	266.34	1								
TPSA	84.58	1								
HBD	3	0.17								
pKa	14.08	0								
CNS MPO	4.17									

HLB = hydrophilic-lipophilic balance; logS = water solubility; PL = polarizability(Å^3); Minimal Projection Area (Å^2); MSA = molecular surface area; RF = Refractivity; HBA = hydrogen bond acceptors; LogBB = blood-brain distribution; ClogP = partitioning; ClogD = distribution at pKa; TPSA = polar surface area; HBD = hydrogen bond donors; CNS MPO = score for CNS penetration.

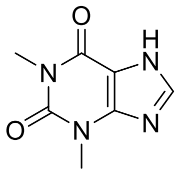

Theophylline
LogBB = - 0.29
ClogBB = - 1.00

Calc.	Marvin 19.20		HLB	logS	PL	MPA	MSA	RF	HBA	LogBB
			13.46	-0.80	16.13	28.19	235.19	44.93	3	-1.00
ClogP	-0.77	1								
ClogD	-0.90	1								
MW	180.17	1								
TPSA	69.30	1								
HBD	1	0.83								
pKa	7.82	1								
CNS MPO	5.82									

HLB = hydrophilic-lipophilic balance; logS = water solubility; PL = polarizability(Å^3); Minimal Projection Area (Å^2); MSA = molecular surface area; RF = Refractivity; HBA = hydrogen bond acceptors; LogBB = blood-brain distribution; ClogP = partitioning; ClogD = distribution at pKa; TPSA = polar surface area; HBD = hydrogen bond donors; CNS MPO = score for CNS penetration.

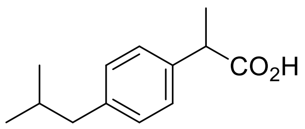

Ibuprofen
LogBB = - 0.18
ClogBB = 0.17

Calc.	Marvin 19.20		HLB	logS	PL	MPA	MSA	RF	HBA	LogBB
			3.79	-1.91	23.65	37.63	356.40	60.73	2	0.17
ClogP	3.84	0.58								
ClogD	1.25	1								
MW	206.28	1								
TPSA	37.30	0.86								
HBD	1	0.83								
pKa	4.85	1								
CNS MPO	5.27									

HLB = hydrophilic-lipophilic balance; logS = water solubility; PL = polarizability($Å^3$); Minimal Projection Area ($Å^2$); MSA = molecular surface area; RF = Refractivity; HBA = hydrogen bond acceptors; LogBB = blood-brain distribution; ClogP = partitioning; ClogD = distribution at pKa; TPSA = polar surface area; HBD = hydrogen bond donors; CNS MPO = score for CNS penetration.

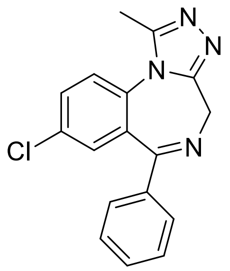

Alprazolam
LogBB = 0.02
ClogBB = -0.04

Calc.	Marvin 19.20		HLB	logS	PL	MPA	MSA	RF	HBA	LogBB
			4.44	-4.11	33.40	50.10	395.11	98.88	3	-0.04
ClogP	3.02	0.99								
ClogD	2.99	0.5								
MW	308.77	1								
TPSA	43.07	1								
HBD	0	1								
pKa	~35	0								
CNS MPO	4.49									

HLB = hydrophilic-lipophilic balance; logS = water solubility; PL = polarizability($Å^3$); Minimal Projection Area ($Å^2$); MSA = molecular surface area; RF = Refractivity; HBA = hydrogen bond acceptors; LogBB = blood-brain distribution; ClogP = partitioning; ClogD = distribution at pKa; TPSA = polar surface area; HBD = hydrogen bond donors; CNS MPO = score for CNS penetration.

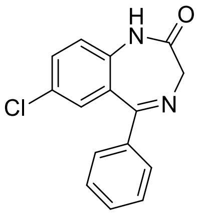

Nordazepam
LogBB = 0.50
ClogBB =0.01

Calc.	Marvin 19.20		HLB	logS	PL	MPA	MSA	RF	HBA	LogBB
			4.52	-4.35	28.48	45.72	339.68	76.70	2	0.01
ClogP	3.21	0.9								
ClogD	3.21	0.4								
MW	270.72	1								
TPSA	41.46	1								
HBD	1	0.83								
pKa	12.30	0								
CNS MPO	4.13									

HLB = hydrophilic-lipophilic balance; logS = water solubility; PL = polarizability(Å^3); Minimal Projection Area (Å^2); MSA = molecular surface area; RF = Refractivity; HBA = hydrogen bond acceptors; LogBB = blood-brain distribution; ClogP = partitioning; ClogD = distribution at pKa; TPSA = polar surface area; HBD = hydrogen bond donors; CNS MPO = score for CNS penetration.

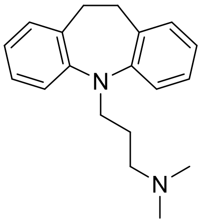

Imipramine
LogBB = 0.83
ClogBB = 0.69

Calc.	Marvin 19.20		HLB	logS	PL	MPA	MSA	RF	HBA	LogBB
			9.28	-2.02	34.67	53.16	477.91	90.61	2	0.69
ClogP	4.28	0.36								
ClogD	2.61	0.70								
MW	280.42	1								
TPSA	6.48	0								
HBD	0	1								
pKa	35	0								
CNS MPO	3.06									

HLB = hydrophilic-lipophilic balance; logS = water solubility; PL = polarizability(Å^3); Minimal Projection Area (Å^2); MSA = molecular surface area; RF = Refractivity; HBA = hydrogen bond acceptors; LogBB = blood-brain distribution; ClogP = partitioning; ClogD = distribution at pKa; TPSA = polar surface area; HBD = hydrogen bond donors; CNS MPO = score for CNS penetration.

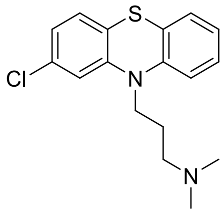

Chloropromazine
LogBB = 1.06
ClogBB = 0.73

Calc.	Marvin 19.20		HLB	logS	PL	MPA	MSA	RF	HBA	LogBB
			11.43	-2.91	35.97	54.40	456.66	93.76	2	0.73
ClogP	4.54	0.23								
ClogD	2.81	0.6								
MW	318.86	1								
TPSA	6.48	0								
HBD	0	1								
pKa	~35	0								
CNS MPO	2.83									

HLB = hydrophilic-lipophilic balance; logS = water solubility; PL = polarizability(Å^3); Minimal Projection Area (Å^2); MSA = molecular surface area; RF = Refractivity; HBA = hydrogen bond acceptors; LogBB = blood-brain distribution; ClogP = partitioning; ClogD = distribution at pKa; TPSA = polar surface area; HBD = hydrogen bond donors; CNS MPO = score for CNS penetration.

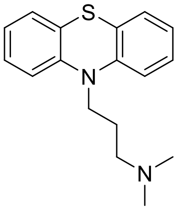

Promazine
LogBB = 1.23
ClogBB = 0.64

Calc.	Marvin 19.20		HLB	logS	PL	MPA	MSA	RF	HBA	LogBB
			11.28	-2.41	34.15	52.56	440.43	88.95	2	0.64
ClogP	3.93	0.53								
ClogD	2.19	0.91								
MW	284.42	1								
TPSA	6.48	0								
HBD	0	1								
pKa	~35	0								
CNS MPO	3.44									

HLB = hydrophilic-lipophilic balance; logS = water solubility; PL = polarizability(Å^3); Minimal Projection Area (Å^2); MSA = molecular surface area; RF = Refractivity; HBA = hydrogen bond acceptors; LogBB = blood-brain distribution; ClogP = partitioning; ClogD = distribution at pKa; TPSA = polar surface area; HBD = hydrogen bond donors; CNS MPO = score for CNS penetration.

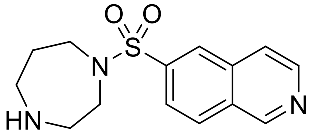

Fasudil

Calc.	Marvin 19.20		HLB	logS	PL	MPA	MSA	RF	HBA	LogBB
			14.63	-1.63	32.22	49.55	422.39	77.92	4	-0.73
ClogP	0.32	1								
ClogD	-0.41	1								
MW	291.37	1								
TPSA	62.30	1								
HBD	1	0.83								
pKa	35	0								
CNS MPO	4.83									

HLB = hydrophilic-lipophilic balance; logS = water solubility; PL = polarizability(Å^3); Minimal Projection Area (Å^2); MSA = molecular surface area; RF = Refractivity; HBA = hydrogen bond acceptors; LogBB = blood-brain distribution; ClogP = partitioning; ClogD = distribution at pKa; TPSA = polar surface area; HBD = hydrogen bond donors; CNS MPO = score for CNS penetration.

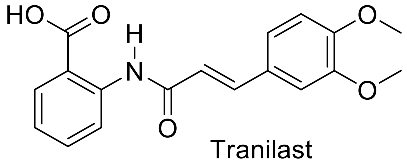

Tranilast

Calc.	Marvin 19.20		HLB	logS	PL	MPA	MSA	RF	HBA	LogBB
			8.43	-0.90	33.85	49.46	453.02	91.52	5	-0.57
ClogP	3.56	0.72								
ClogD	0.21	1								
MW	327.34	1								
TPSA	84.86	1								
HBD	2	0.5								
pKa	3.53	1								
CNS MPO	5.22									

HLB = hydrophilic-lipophilic balance; logS = water solubility; PL = polarizability(Å^3); Minimal Projection Area (Å^2); MSA = molecular surface area; RF = Refractivity; HBA = hydrogen bond acceptors; LogBB = blood-brain distribution; ClogP = partitioning; ClogD = distribution at pKa; TPSA = polar surface area; HBD = hydrogen bond donors; CNS MPO = score for CNS penetration.

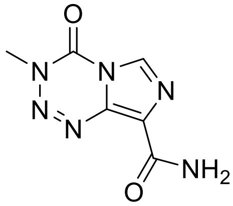

Temozolomine

Calc.	Marvin 19.20		HLB	logS	PL	MPA	MSA	RF	HBA	LogBB
			14.72	-0.89	16.8	26.87	232.02	47.86	5	-1.47
ClogP	-0.28	1								
ClogD	-0.28	1								
MW	194.15	1								
TPSA	105.94	0.47								
HBD	2	0.5								
pKa	10.46	0								
CNS MPO	3.97									

HLB = hydrophilic-lipophilic balance; logS = water solubility; PL = polarizability(Å^3); Minimal Projection Area (Å^2); MSA = molecular surface area; RF = Refractivity; HBA = hydrogen bond acceptors; LogBB = blood-brain distribution; ClogP = partitioning; ClogD = distribution at pKa; TPSA = polar surface area; HBD = hydrogen bond donors; CNS MPO = score for CNS penetration.

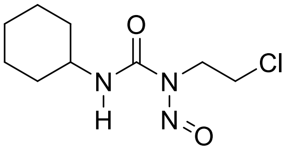

Lomustine

Calc.	Marvin 19.20		HLB	logS	PL	MPA	MSA	RF	HBA	LogBB
			8.42	-3.28	21.92	33.91	351.15	58.65	2	-0.45
ClogP	2.16	1								
ClogD	2.16	0.92								
MW	233.70	1								
TPSA	61.77	1								
HBD	1	0.83								
pKa	14.05	0								
CNS MPO	4.75									

HLB = hydrophilic-lipophilic balance; logS = water solubility; PL = polarizability(Å^3); Minimal Projection Area (Å^2); MSA = molecular surface area; RF = Refractivity; HBA = hydrogen bond acceptors; LogBB = blood-brain distribution; ClogP = partitioning; ClogD = distribution at pKa; TPSA = polar surface area; HBD = hydrogen bond donors; CNS MPO = score for CNS penetration.

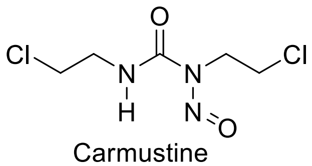

Carmustine

Calc.	Marvin 19.20		HLB	logS	PL	MPA	MSA	RF	HBA	LogBB
			10.81	-2.38	17.32	29.68	275.91	46.98	2	-0.62
ClogP	1.02									
ClogD	1.02									
MW	214.05									
TPSA	61.77									
HBD	1									
pKa	13.36									
CNS MPO	4.83									

HLB = hydrophilic-lipophilic balance; logS = water solubility; PL = polarizability($Å^3$); Minimal Projection Area ($Å^2$); MSA = molecular surface area; RF = Refractivity; HBA = hydrogen bond acceptors; LogBB = blood-brain distribution; ClogP = partitioning; ClogD = distribution at pKa; TPSA = polar surface area; HBD = hydrogen bond donors; CNS MPO = score for CNS penetration.

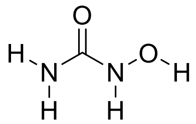

Hydroxycarbamide (hydroxyurea)

Calc.	Marvin 19.20		HLB	logS	PL	MPA	MSA	RF	HBA	LogBB
			14.60	0.74	5.76	16.00	102.14	14.91	2	-1.18
ClogP	-1.37	1								
ClogD	-1.37	1								
MW	76.06	1								
TPSA	75.35	1								
HBD	3	0.17								
pKa	10.14	0								
CNS MPO	4.17									

HLB = hydrophilic-lipophilic balance; logS = water solubility; PL = polarizability($Å^3$); Minimal Projection Area ($Å^2$); MSA = molecular surface area; RF = Refractivity; HBA = hydrogen bond acceptors; LogBB = blood-brain distribution; ClogP = partitioning; ClogD = distribution at pKa; TPSA = polar surface area; HBD = hydrogen bond donors; CNS MPO = score for CNS penetration.

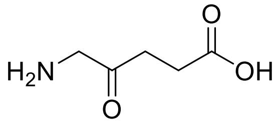

Gliolan (Aminolevulinic acid)

Calc.	Marvin 19.20		HLB	logS	PL	MPA	MSA	RF	HBA	LogBB
			16.82	0.75	12.15	24.44	203.06	30.45	4	-1.20
ClogP	-0.99	1								
ClogD	-3.41	1								
MW	131.13	1								
TPSA	80.39	1								
HBD	2	0.5								
pKa	4.05	1								
CNS MPO	5.5									

HLB = hydrophilic-lipophilic balance; logS = water solubility; PL = polarizability(Å^3); Minimal Projection Area (Å^2); MSA = molecular surface area; RF = Refractivity; HBA = hydrogen bond acceptors; LogBB = blood-brain distribution; ClogP = partitioning; ClogD = distribution at pKa; TPSA = polar surface area; HBD = hydrogen bond donors; CNS MPO = score for CNS penetration.

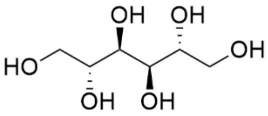

Mannitol
LogBB = - 2.51
ClogBB = - 2.22

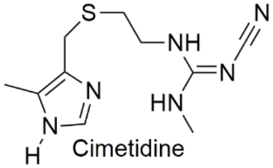

Cimetidine
LogBB = - 1.42
ClogBB = - 1.19

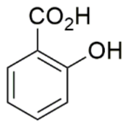

Salicylate
LogBB = - 1.10
ClogBB = - 0.61

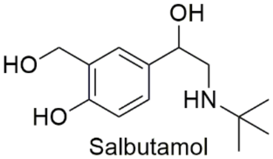

Salbutamol
LogBB = - 1.03
ClogBB = - 0.80

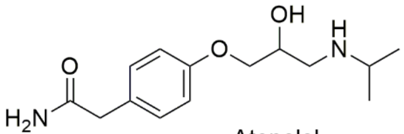

Atenolol
LogBB = - 0.87
ClogBB = - 1.05

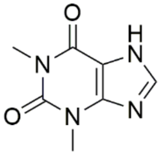

Theophylline
LogBB = - 0.29
ClogBB = - 1.00

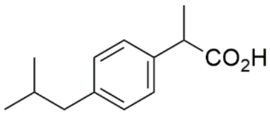

Ibuprofen
LogBB = - 0.18
ClogBB = 0.17

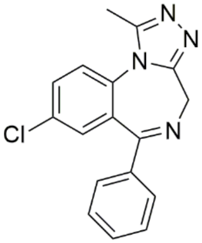

Alprazolam
LogBB = 0.02
ClogBB = - 0.04

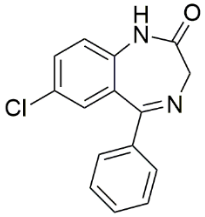

Nordazepam
LogBB = 0.50
ClogBB =0.01

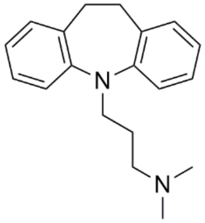

Imipramine
LogBB = 0.83
ClogBB = 0.69

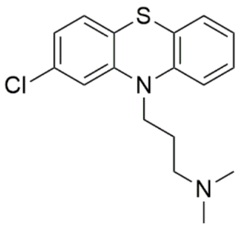

Chloropromazine
LogBB = 1.06
ClogBB = 0.73

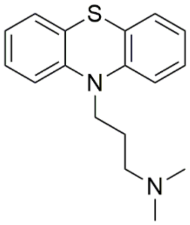

Promazine
LogBB = 1.23
ClogBB = 0.64

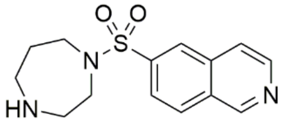

Fasudil
ClogBB = - 0.73

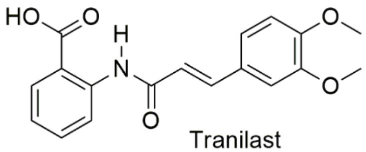

Tranilast
ClogBB = - 0.57

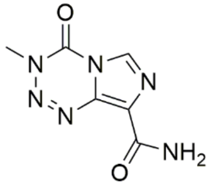

Temozolomine
ClogBB = - 1.47

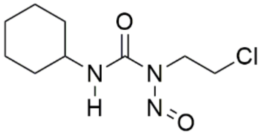

Lomustine
ClogBB = - 0.45

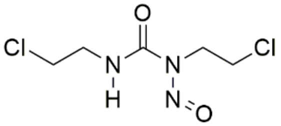

Carmustine
ClogBB = - 0.62

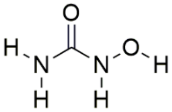

Hydroxycarbamide
ClogBB = - 1.18

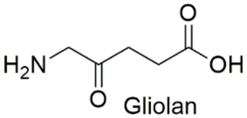

Gliolan
ClogBB = - 1.20

	Comp.	logP	PSA	A+D	[a]LogBB	[b]ClogBB	[c]RlogBB	[c]ALofBB	[d]BlogBB	[f]DlogBB	CNS
1	Mannitol	-3.73	121.38	12	-2.51	-2.23	-1.63	-1.40	-1.46	-9.27	3.00
2	Cimetidine	-0.11	88.89	8	-1.42	-1.19	-0.74	-0.60	-0.63	-5.12	3.84
3	Salicylate	0.65	57.53	5	-1.10	-0.61	-0.31	-0.24	-0.26	-2.22	5.50
4	Salbutamol	0.88	72.72	8	-1.03	-0.80	-0.43	-0.32	-0.34	-4.36	4.00
5	Atenolol	0.43	84.58	7	-0.87	-1.05	-0.62	-0.49	-0.51	-4.29	4.17
6	Theophylline	-0.77	69.30	4	-0.29	-1.00	-0.65	-0.54	-0.58	-2.36	5.82
7	Ibuprofen	3.84	37.30	3	-0.18	0.17	0.39	0.40	0.40	0.30	5.27
8	Alprazolam	3.02	43.07	3	0.02	-0.04	0.20	0.23	0.23	-0.07	4.44
9	Nordazepam	3.21	41.46	3	0.50	0.01	0.25	0.27	0.27	0.02	4.13
10	Imipramine	4.28	6.48	2	0.83	0.69	0.76	0.71	0.72	1.98	3.09
11	Chlorpromazine	4.54	6.48	2	1.06	0.74	0.80	0.75	0.76	2.04	2.83
12	Promazine	3.93	6.48	2	1.23	0.64	0.71	0.66	0.67	1.90	3.44
13	Fasudil	0.32	62.30	5		-0.74	-0.41	-0.32	-0.35	-2.45	4.83
14	Tranilast	3.56	84.86	7		-0.58	-0.13	-0.02	-0.02	-3.58	5.22
15	Temozolomine	-0.28	105.94	7		-1.47	-0.94	-0.76	-0.79	-5.15	3.97
16	Lomustine	2.16	61.77	3		-0.45	-0.12	-0.05	-0.05	-0.88	4.72
17	Carmustine	1.02	61.77	3		-0.62	-0.30	-0.22	-0.23	-1.14	4.83
18	Hydroxycarbamide	-1.37	75.35	5		-1.18	-0.80	-0.68	-0.72	-3.26	4.17
19	Gliolan	-0.99	80.39	6		-1.20	-0.79	-0.66	-0.70	-3.91	5.5

[a]Experimental LogBB: experimental values are taken from compile logBB in Carpenter, T. S.; Krishner, D. A.; Lau, E. Y.; Wong, S. E.; Nilmeier, J. P.; Lightstone "A Method to Predict Blood-Brain Barrier Permeability of Drug-Like Compounds Using Molecular Dynamics Simulations" *Biophysical Journal* **2014**, *107*, 630-641.

[b]Clark: ClogBB = 0.152 x logP - 0.0148 x PSA + 0.139.

[c]Rishton: RlogBB = 0.155 x logP - 0.01 x PSA +0.164.

[d]From first larger fitting set (371 compounds): AlogBB = 0.149 x logP – 0.008 x PSA + 0.127.

[e]From second smaller fitting set (from 274 compounds): BlogBB = 0.157 x logP – 0.008 x PSA + 0.1.

[f]DlogBB = 0.2289 × log P – 0.0326 × PSA – 0.5671 × (D + A) + 2.342.

Equations were generated from date obtained from studies performed in Chevalier, R. "Penetration Assessment od Dietary Supplements and Drugs through the Blood-Brain Barrier for Potential Treatment of Parkinson's Disease" bioRxiv, **doi:** https://doi.org/10.1101/362541, 2018.

Nine Compounds with excellent anti-glioblastoma activity (LN229) at 25 µM

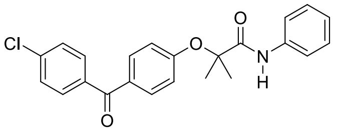

HR48 (PP98) CV = (3.19 ± 3.64) %;
MPA = 43.73 Å^2; PL = 42.67 Å^3;
RF = 111.47 m^3 mol^{-1}; logS = -7.07 mol/l

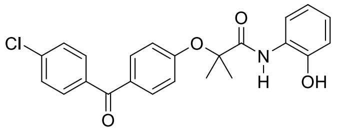

HR49 (PP99) CV = (0 ± 0.27) %;
MPA = 43.75 Å^2; PL = 43.28 Å^3;
RF = 113.45 m^3 mol^{-1}; logS = = - 6.61 mol/l

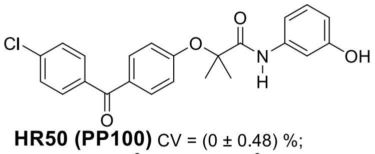

HR50 (PP100) CV = (0 ± 0.48) %;
MPA = 45.22 Å^2; PL = 43.28 Å^3;
RF = 113.45 m^3 mol^{-1}; LogS = - 6.61 mol/l

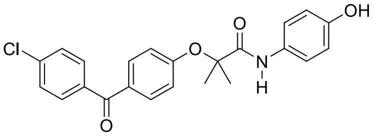

HR51 (PP12) CV = (1.1 ± 1.42) %;
MPA = 49.70 Å^2; PL = 43.28 Å^3;
RF = 113.45 m^3 mol^{-1}; logS = - 6.61 mol/l

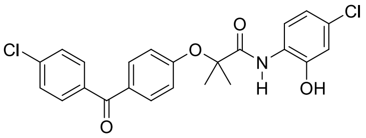

HR54 (PP184) CV = (1.89 ± 1.56) %;
MPA = 46.41 Å^2; PL = 45.17 Å^3;
RF = 118.26 m^3 mol^{-1}; logS = - 7.08 mol/l

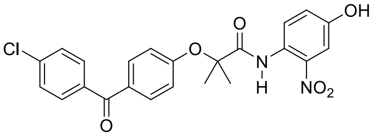

HR57 (PP182) CV = (1.29 ± 2.79) %;
MPA = 47.50 Å^2; PL = 45.20 Å^3;
RF = 119.77 m^3 mol^{-1}; logS = -7.05 mol/l

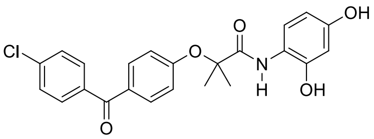

HR59 (PP171) CV = (0.88 ± 1.50) %;
MPA = 44.25 Å^2; PL = 43.90 Å^3;
RF = 115.43 m^3 mol^{-1}; logS = -6.11 mol/l

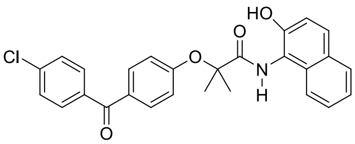

HR61 (PP201) CV = (2.87 ± 1.05) %;
MPA = 48.22 Å^2; PL = 50.79 Å^3;
RF = 129.90 m^3 mol$^{-1;}$ logS = -8.28 mol/l

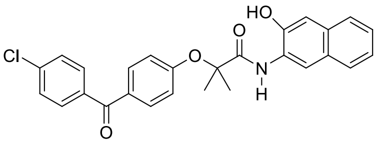

HR65 (PP197) CV = (1.49 ± 0.32) %;
MPA = 50.52 Å^2; PL = 50.78 Å^3;
RF = 129.90 m^3 mol^{-1}; LogS = -8.29 mol/l

DMSO

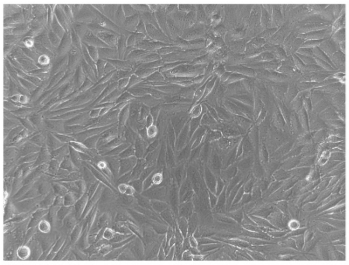

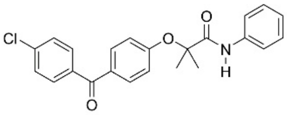

HR48 (PP98) CV = (3.19 ± 3.64) %;
MPA = 43.73 Å^2; PL = 42.67 Å^3;
RF = 111.47 m^3 mol^{-1}; logS = -7.07 mol/l

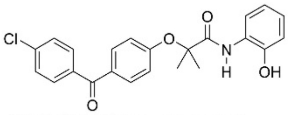

HR49 (PP99) CV = (0 ± 0.27) %;
MPA = 43.75 Å^2; PL = 43.28 Å^3;
RF = 113.45 m^3 mol^{-1}; logS = = - 6.61 mol/l

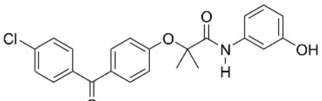

HR50 (PP100) CV = (0 ± 0.48) %;
MPA = 45.22 Å^2; PL = 43.28 Å^3;
RF = 113.45 m^3 mol^{-1}; LogS = - 6.61 mol/l

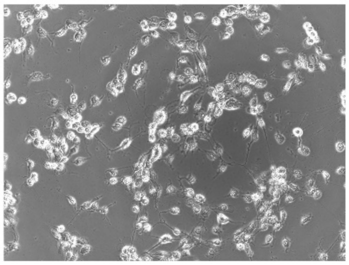

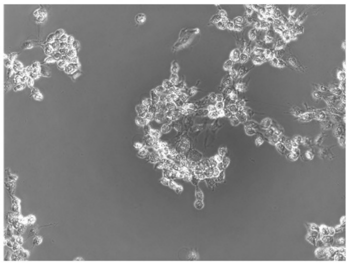

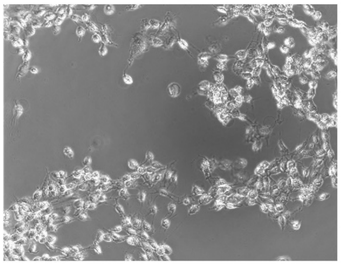

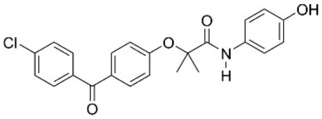

HR51 (PP12) CV = (1.1 ± 1.42) %;
MPA = 49.70 Å^2; PL = 43.28 Å^3;
RF = 113.45 m^3 mol^{-1}; logS = - 6.61 mol/l

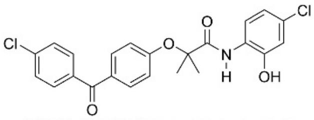

HR54 (PP184) CV = (1.89 ± 1.56) %;
MPA = 46.41 Å^2; PL = 45.17 Å^3;
RF = 118.26 m^3 mol^{-1}; logS = - 7.08 mol/l

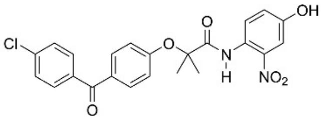

HR57 (PP182) CV = (1.29 ± 2.79) %;
MPA = 47.50 Å^2; PL = 45.20 Å^3;
RF = 119.77 m^3 mol^{-1}; logS = -7.05 mol/l

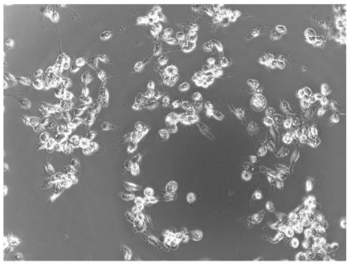

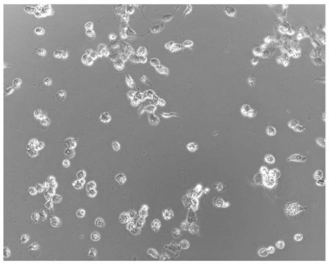

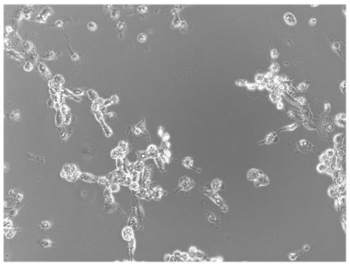

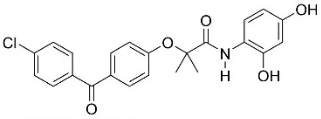

HR59 (PP171) CV = (0.88 ± 1.50) %;
MPA = 44.25 Å^2; PL = 43.90 Å^3;
RF = 115.43 m^3 mol^{-1}; logS = -6.11 mol/l

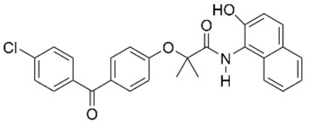

HR61 (PP201) CV = (2.87 ± 1.05) %;
MPA = 48.22 Å^2; PL = 50.79 Å^3;
RF = 129.90 m^3 mol$^{-1;}$ logS = -8.28 mol/l

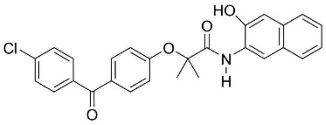

HR65 (PP197) CV = (1.49 ± 0.32) %;
MPA = 50.52 Å^2; PL = 50.78 Å^3;
RF = 129.90 m^3 mol^{-1}; LogS = -8.29 mol/l

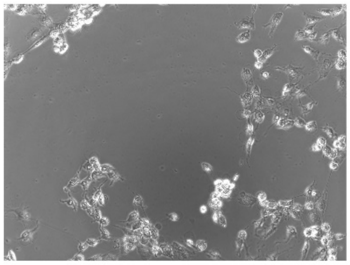

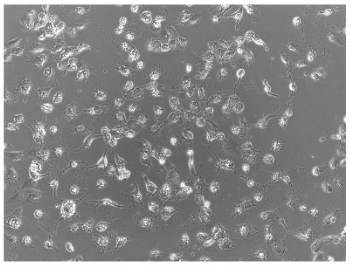

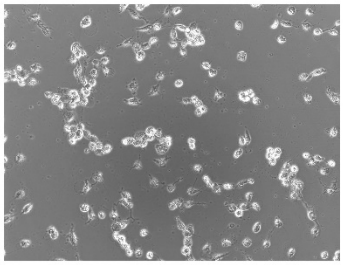

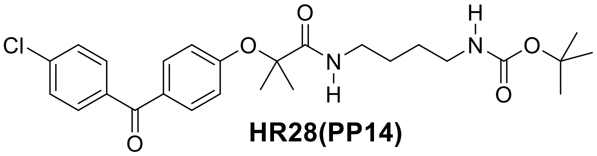

HR28(PP14)

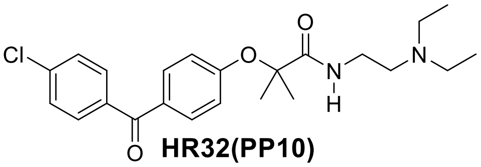

HR32(PP10)

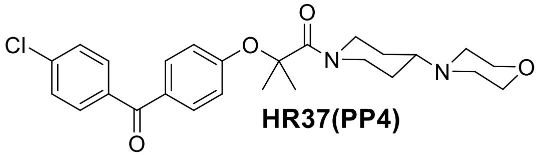

HR37(PP4)

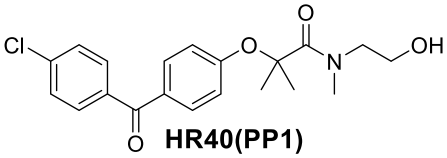

HR40(PP1)

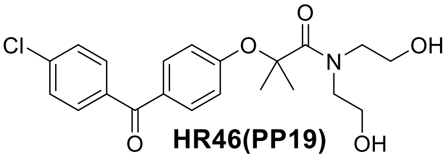

HR46(PP19)

Comp.	logP	PSA	D+A	M1LogBB	M2LogBB	M3LogBB	M4LogBB	MPO
**HR28**	5.05	93.73	6	-0.34	-0.48	0.01	-2.96	1.62
**HR32**	4.51	58.64	5	0.36	-0.04	0.28	-1.37	2.97
**HR37**	3.81	59.08	5	-0.02	-0.16	0.16	-1.55	3.06
**HR40**	3.31	66.84	5	-0.49	-0.35	0.01	-1.91	3.97
**HR46**	2.62	87.07	7	-1.41	-0.75	-0.30	-3.87	3.86

Computed new LogBB values for our previously reported active compounds.
ClogP = calculated partitioning; PSA = Polar surface area ($Å^2$); D = hydrogen bond donor at pH = 7; A = hydrogen bond acceptor at pH =7; MPO = Central nervous system multiparameter optimization (CNS MPO).
M1LogBB = 0.5159 × log P − 0.0277×PSA−0.3462. For log BB≥0.3 [Vilar, S.; Chakrabarti, M.; Costanzi, S. "Prediction of passive blood-brain partitioning: Straightforward and effective classification models based on silico derived physicochemical descriptors" *Journal of Molecular Graphics and Modelling* 2010, 28, 899-903.]
M2logBB = 0.152logP - 0.0148PSA + 0.139. (Clark's model) from: Clark, D. E. "Rapid Calculation of Polar Molecular Surface Area and Its Application to the Prediction of Transport Phenomena. 2. Prediction of Blood-Brain Barrier Prediction" *Journal of Pharmaceutical Sciences* 1999, 88, 815-821.
M3logBB = 0.155 x logP - 0.01 x PSA +0.164. (Rishton's model) from Rishton, G. M.; LaBonte, K.; Williams, A. J.; Kassam, K.; Kolovanov, E. "Computational approaches to the prediction of blood-brain barrier permeability: A comparative analysis of central nervous system drugs secretase inhibitors for Alzheimer's disease" *Curr. Opin. Drug. Discov. Devel.* 2006, 9, 303-313.
M4log BB = 0.2289 × log P − 0.0326 × PSA − 0.5671 × (D + A) + 2.3420. For log BB≥−1 [Vilar, S.; Chakrabarti, M.; Costanzi, S. "Prediction of passive blood-brain partitioning: Straightforward and effective classification models based on silico derived physicochemical descriptors" *Journal of Molecular Graphics and Modelling* 2010, 28, 899-903.]
These compounds are previously published in: Stalinska, J.; Houser, L.; Rak, M.; Colley, S. B.; Reiss, K.; Jursic, B. S. "Exploring anticancer activity of structurally modified benzylphenoxyacetamide (BPA); I: Synthesis strategies and computational analyses of substituted BPA variants with high anti-glioblastoma potential" *Nature: Scientific Reports* 2019.
